# A dynamic, ring-forming MucB / RseB-like protein influences spore shape in *Bacillus subtilis*

**DOI:** 10.1371/journal.pgen.1009246

**Published:** 2020-12-14

**Authors:** Johana Luhur, Helena Chan, Benson Kachappilly, Ahmed Mohamed, Cécile Morlot, Milena Awad, Dena Lyras, Najwa Taib, Simonetta Gribaldo, David Z. Rudner, Christopher D. A. Rodrigues

**Affiliations:** 1 The ithree Institute, University of Technology Sydney (UTS), Australia; 2 University Grenoble Alpes, CNRS, CEA, IBS, Grenoble, France; 3 Infection and Immunity Program, Monash Biomedicine Discovery Institute and Department of Microbiology, Monash University, Melbourne, Australia; 4 Department of Microbiology, Unit Evolutionary Biology of the Microbial Cell, Institut Pasteur, UMR 2001 CNRS, Paris, France; 5 Hub Bioinformatics and Biostatistics, Department of Computational Biology, Institut Pasteur, USR 3756 CNRS, Paris, France; 6 Department of Microbiology, Harvard Medical School, Boston, United States of America; Indiana University, UNITED STATES

## Abstract

How organisms develop into specific shapes is a central question in biology. The maintenance of bacterial shape is connected to the assembly and remodelling of the cell envelope. In endospore-forming bacteria, the pre-spore compartment (the forespore) undergoes morphological changes that result in a spore of defined shape, with a complex, multi-layered cell envelope. However, the mechanisms that govern spore shape remain poorly understood. Here, using a combination of fluorescence microscopy, quantitative image analysis, molecular genetics and transmission electron microscopy, we show that SsdC (formerly YdcC), a poorly-characterized new member of the MucB / RseB family of proteins that bind lipopolysaccharide in diderm bacteria, influences spore shape in the monoderm *Bacillus subtilis*. Sporulating cells lacking SsdC fail to adopt the typical oblong shape of wild-type forespores and are instead rounder. 2D and 3D-fluorescence microscopy suggest that SsdC forms a discontinuous, dynamic ring-like structure in the peripheral membrane of the mother cell, near the mother cell proximal pole of the forespore. A synthetic sporulation screen identified genetic relationships between *ssdC* and genes involved in the assembly of the spore coat. Phenotypic characterization of these mutants revealed that spore shape, and SsdC localization, depend on the coat basement layer proteins SpoVM and SpoIVA, the encasement protein SpoVID and the inner coat protein SafA. Importantly, we found that the Δ*ssdC* mutant produces spores with an abnormal-looking cortex, and abolishing cortex synthesis in the mutant largely suppresses its shape defects. Thus, SsdC appears to play a role in the proper assembly of the spore cortex, through connections to the spore coat. Collectively, our data suggest functional diversification of the MucB / RseB protein domain between diderm and monoderm bacteria and identify SsdC as an important factor in spore shape development.

## Author summary

Cell shape is an important cellular attribute linked to cellular function and environmental adaptation. Bacterial endospores are one of the toughest cell types on Earth, with a defined shape and complex, highly-resistant, multi-layered cell envelope. Although decades of research have focused on defining the composition and assembly of the multi-layered spore envelope, little is known about how these layers contribute to spore shape. Here, we identify SsdC, a poorly-characterized new member of the MucB / RseB family of proteins that bind lipopolysaccharide in diderm bacteria. We show that SsdC is an important factor in spore shape development in the monoderm, model organism *Bacillus subtilis*. Our data suggest that SsdC influences the assembly of the spore cortex, through connections to the spore coat, by forming an intriguing, dynamic ring-like structure adjacent to the developing spore. Furthermore, our identification of SsdC suggests evolutionary diversification of the MucB /RseB protein domain between diderm and monoderm bacteria.

## Introduction

Bacterial cell shape is an important characteristic that plays a fundamental role in survival [1,2]. In growing bacteria, a plethora of studies have shown that the cell envelope plays a critical role in shape, with the peptidoglycan cell wall being a major shape determinant (reviewed by [3–5]). However, little is known about how the multi-layered spore envelope influences spore shape. Furthermore, although spore formation is largely restricted to monoderm, Gram-positive bacteria, the spore contains a diderm cell envelope, similar to diderm, Gram-negative bacteria. Thus, spore development is an interesting model system for dissecting the similarities and differences of building the cell envelope, and its relationship to shape, in monoderm and diderm bacteria, with potential ramifications for understanding the evolution of the bacterial envelope [6–9]. Here we focus on the determinants of spore shape through the characterization of a poorly-characterized *Bacillus subtilis* protein called SsdC (formerly YdcC), an orthologue of the outer membrane stress response proteins, MucB / RseB found in Gram-negative bacteria [10].

Spore development is intimately tied to the morphogenesis of its envelope. In response to starvation, spore-forming bacteria divide asymmetrically, generating two genetically identical cells of different size: the smaller is the forespore (that later matures into the spore) and the larger is the mother cell (henceforth the mother) (reviewed by [11–12]). Each of these cells has a distinct but highly-coordinated developmental program governed by sequentially-activated, cell-specific sigma factors: σ^F^ and σ^G^ in the forespore and σ^E^ and σ^K^ in the mother cell. Initially, these two cells lie side-by-side. Shortly after, the mother membranes migrate around the forespore in a phagocytic-like process known as engulfment. Engulfment is an essential stage in spore envelope morphogenesis as it generates their characteristic double-membrane envelope: an inner membrane originating from the forespore itself and outer membrane derived from the mother cell, with a periplasmic-like space in between, containing a thin layer of peptidoglycan called the germ cell wall. Importantly, during engulfment, founder proteins are recruited to the outer spore membrane to orchestrate the assembly of the multi-layered spore coat (reviewed by [13–14]). Membrane fission at the end of engulfment releases the forespore into the mother cytoplasm, where it continues to mature by the addition of a thick layer of modified peptidoglycan called the cortex cell wall (built on top of the germ cell wall) [15]. When the spore has matured, the mother lyses and releases the dormant spore into the environment, where its complex envelope plays a vital role in stress resistance [16].

During spore maturation, the multi-layered coat is deposited on top of the outer spore membrane and consists of the basement layer, inner coat, outer coat, and crust [13–14]. The coat is built in an orderly manner from the innermost to the outermost layer [13–14]. The innermost layer of the coat is the basement layer that anchors the other layers of the coat to the spore outer membrane. It is formed by the morphogenetic proteins SpoVM and SpoIVA [13–14]. SpoVM is a small amphipathic α-helix peptide that binds to the forespore outer membrane via hydrophobic interactions [17]. SpoVM localizes to positive membrane curvature on the mother cell side of the forespore membrane at the initiation of engulfment and the polar side chain of the hydrophilic face of the SpoVM helix interacts and recruits SpoIVA to the outer forespore membrane [17]. SpoIVA binds and hydrolyses ATP to self-assemble and multimerise into cable-like structures around the spore that constitute the basement layer [18]. The localization of SpoIVA and SpoVM is interdependent and loss of either of these proteins affects the formation of the spore coat [17]. In the absence of SpoIVA, the coat is not attached to the spore, but forms clusters in the mother cell [19]. Moreover, in the absence of SpoIVA, the spore cortex layer does not form [19]. This suggests that SpoIVA is required for assembly of both the cortex and coat and that the two processes could be coordinated. Similarly, in the absence of SpoVM, the spore cortex is absent and the coat is partially attached and disorganised [20].

The assembly of the inner coat requires SpoVID and SafA, which depend on SpoIVA for their targeting to the forespore [14,21]. SpoVID and SafA also interact with each other to form a complex [22]. The outer coat is assembled by another morphogenetic protein, CotE, which depends on SpoIVA and SpoVID for its localization [23].

The assembly of coat layers requires two major steps: localization and encasement [11,14]. Most coat genes are under the control of early mother cell-specific σ^E^ and late mother cell-specific σ^K^ [24]. Production and localization of spore coat proteins starts immediately after asymmetric division, but coat proteins are added throughout sporulation [24]. Initially, the coat morphogenetic proteins localize as an organised scaffold cap on the mother cell proximal (MCP) pole of the forespore [24]. Following engulfment, the proteins eventually encircle the entire spore to form complete concentric rings in a process called encasement [21,25]. Encasement is driven by SpoVM and SpoVID [14].

Synthesis of the spore cortex requires two proteins, SpoVD and SpoVE [15,26]. SpoVD is a class B penicillin-binding protein (PBP) with transpeptidase activity and SpoVE belongs to the SEDS (Shape, Elongation, Division, Sporulation) family of proteins that is predicted to have glycosyltransferase activity [15]. Furthermore, although cortex peptidoglycan is initially similar to vegetative peptidoglycan, it is then modified: stem peptides are removed and muramic δ-lactam is introduced [15]. Muramic δ-lactam is not required for heat-resistance but functions as a specificity determinant for enzymatic cortex degradation during spore germination [15].

Although research has focused on defining the composition and assembly of the multi-layered spore envelope [11,14], little is known about how these layers contribute to spore shape. In this work, we show that SsdC (formerly YdcC), a new member of the MucB / RseB family of proteins contributes to spore shape through connections to the spore envelope. Our data suggest that SsdC has structural similarity to the MucB / RseB family of proteins found in Gram-negative bacteria and that sporulating cells lacking SsdC produce rounder spores that are defective in spore germination. We also demonstrate that SsdC localization is dynamic, transitioning through a ring-like intermediate near the MCP pole of the forespore that disassembles as the spore matures. Following a transposon-sequencing screen in the *ssdC* mutant, we further show that SsdC localization and spore shape, depends on various spore coat proteins. Finally, we provide data suggesting that cortex synthesis occurs aberrantly in the absence of SsdC, leading to spore shape defects. Collectively, our data suggest that SsdC is an important factor in spore shape development and suggest that spore shape is not only influenced by assembly of the cortex but also by the coat. Furthermore, our data suggest functional diversification of the MucB / RseB protein domain between diderm bacteria and monoderm Firmicutes and lends support to the idea of an endospore-forming diderm ancestor of the Firmicutes, with functional repurposing of MucB / RseB after the loss of the outer membrane in the Bacillales. Therefore, SsdC is likely vestigial evidence of an evolutionary past involving diderm biology that still exists in some Firmicutes today.

## Results

### SsdC has structural similarity to RseB

**Fig 1:**
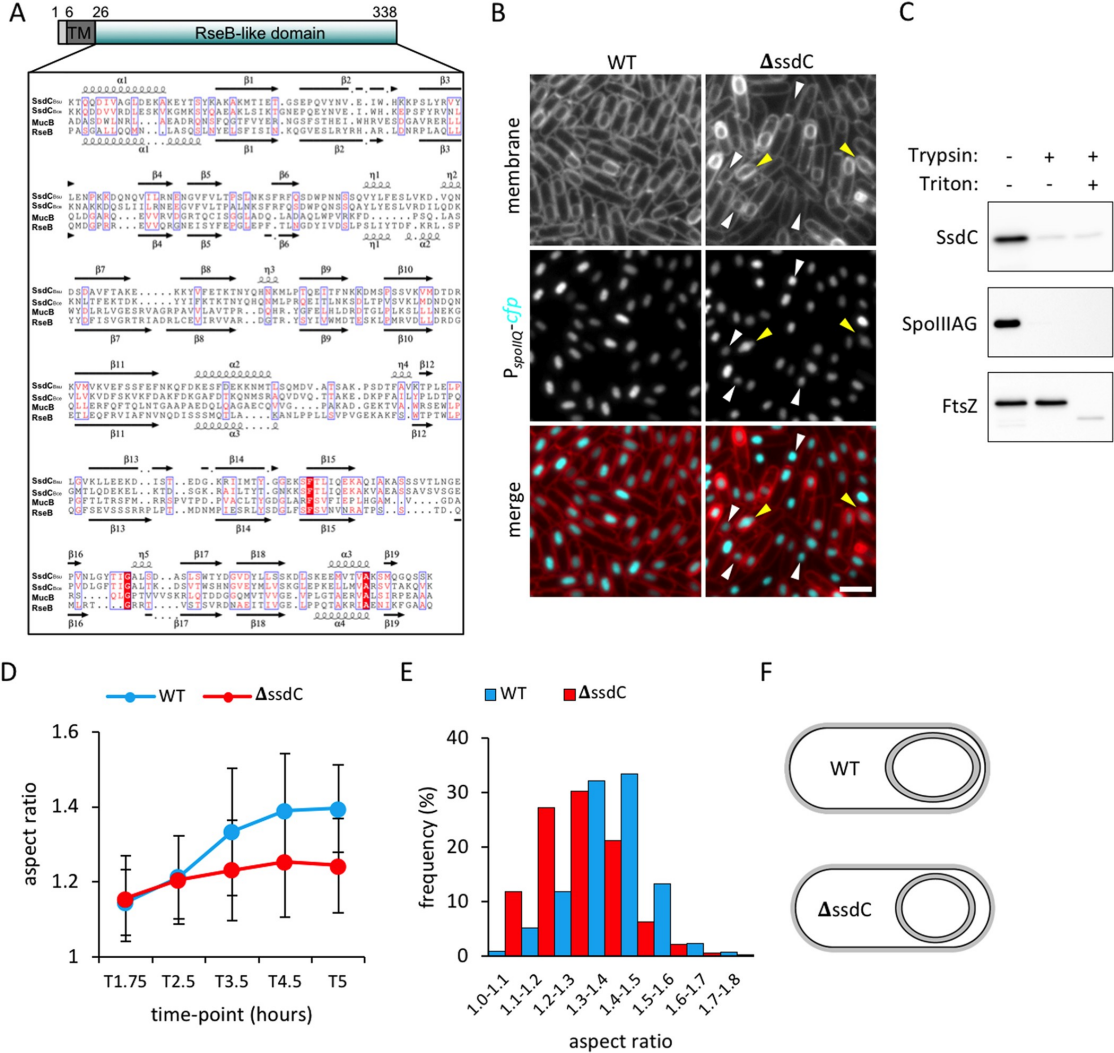
SsdC has structural similarity to RseB and its mutant produces rounder forespores. **(A)** Multiple sequence alignment of the RseB-like domain of *B*. *subtilis* SsdC (SsdC_Bsu_) (aa 26–338), *B*. *cereus* SsdC (SsdC_Bce_), *Pseudomonas aeruginosa* MucB (MucB) and *Escherichia coli* RseB (RseB). Predicted secondary structures are annotated above and below the alignment; coils indicate α-helices, arrows indicate β-sheets. Conserved residues are shown in red and boxed. Highly-conserved residues are shaded in red. The predicted N-terminal tail (aa 1–6) and transmembrane segment (aa 7–25, TM) of *B*. *subtilis* SsdC were not included in the alignment. **(B)** Forespore morphology of wild-type (WT, bBK17) and Δ*ssdC* (bBK18) strains at 3.5 h after onset of sporulation (T3.5). Forespore cytoplasm was visualised using a forespore reporter (P_*spoIIQ*_-*cfp*, false-coloured cyan in merged images). Cell membranes were visualised with TMA-DPH fluorescent membrane dye and are false-coloured red in merged images. White arrowheads point to round forespores and yellow arrowheads to irregularly-shaped forespores. Scale bar = 2 μm. **(C)** SsdC is surface exposed and thus accessible to trypsin digestion. Immunoblot analysis using anti-His antibodies of protoplasted sporulating cells (bHC70) treated with Trypsin in the presence and absence of TritonX-100. Consistent with the idea that SsdC is membrane-anchored, it remained cell-associated after the generation of protoplasts. As controls, the immunoblot was probed for a membrane protein with an extracellular domain (SpoIIIAG) and a cytoplasmic protein (FtsZ). **(D)** Average forespore aspect ratio (± STDEVP) of wild-type (WT, bBK17, blue) and Δ*ssdC* (bBK18, red) strains during a sporulation time-course. *n* > 500 per time-point, per strain. **(E)** Frequency distribution of forespore aspect ratios of wild-type (WT, bBK17, blue) and Δ*ssdC* (bBK18, red) strains at 5 h (T5) after onset of sporulation (T5). *n* > 1000 per strain. **(F)** Schematic representation of forespore shape in wild-type and the Δ*ssdC* mutant.

In the course of our analysis of new sporulation genes identified by transposon-sequencing [27], we examined a subset of previously identified sporulation mutants by fluorescence microscopy. One of these genes was *ydcC* (renamed *ssdC*, spore shape determinant C), a sporulation-induced gene identified by microarray analysis at the beginning of the millennium [28–29]. The *ssdC* gene is expressed in the mother-cell compartment under σ^E^ control [28–29] and encodes a 338-amino acid polypeptide, predicted to contain an N-terminal transmembrane segment and a large extracellular C-terminal domain (Fig 1A). Consistent with this prediction, we found that a functional SsdC-His6 fusion (S1C Fig) was membrane-associated and susceptible to trypsin cleavage in a protease accessibility assay (Fig 1C).

Interestingly, a search for remote homologues using the HHPRED server (https://toolkit.tuebingen.mpg.de/tools/hhpred)) [30] identified RseB, a protein involved in the negative regulation of RpoE (σ^E^) in Gram-negative bacteria (Fig 1A). Despite poor sequence identity between the two proteins (12%), SsdC regions displaying sequence similarity with RseB were modelled with acceptable confidence factors (mean value ± SD, 0.52 ± 0.08) using the Swiss Model Server (http://swissmodel.expasy.org//) [31]. Finally, in further support of a structural homology between SsdC and RseB, secondary structures predicted independently of RseB using the Jpred4 server (http://www.compbio.dundee.ac.uk/jpred4) [32] matched the length and position of helices and strands in the RseB-based model (Fig 1A). The predicted structural similarity between SsdC and RseB prompted our investigation into its role during sporulation.

### SsdC is required for the oblong shape of the forespore

We previously reported that after engulfment, cells lacking *ssdC* produce rounder-looking forespores that appear slightly offset from one pole of the mother cell [27] (Fig 1B and 1F). This is in contrast to wild-type forespores that are oblong and localize adjacent to one pole of the mother cell (Fig 1B and 1F). To more thoroughly characterize the morphological defect of the Δ*ssdC* mutant, we quantitatively examined the aspect ratio of individual forespores using MicrobeJ [33] (see Materials & Methods). To facilitate image analysis, we expressed a cyan fluorescent protein (CFP) in the forespore cytoplasm and analysed the fluorescent signal during a sporulation time-course. Consistent with previous observations, the round forespore phenotype of the Δ*ssdC* mutant only became apparent at 3.5 h of sporulation (T3.5), when most wild-type and Δ*ssdC* mutant cells had completed engulfment (Fig 1D). While the aspect ratio of wild-type forespores increased over time and plateaued at T4, the aspect ratio of the Δ*ssdC* mutant forespores failed to increase beyond T3.5 and remained at a similar level until T5. At T5, the smaller aspect ratio of the Δ*ssdC* mutant compared to the wild-type was most pronounced (Fig 1E). Importantly, a comparison (using the Kolmogorov-Smirnov test) of the distribution of forespore aspect ratios in wild-type and Δ*ssdC* mutant backgrounds indicates that the differences are statistically significant (p<0.001) at T3.5, T4.5, and T5, but not at T1.75 and T2.5. Thus, the Δ*ssdC* mutant produces rounder forespores. Interestingly, the Δ*ssdC* mutant had a second, albeit less penetrant, phenotype that we described as “irregular” forespores. These forespores appeared more elongated and distorted (Fig 1B) (see below).

Since SsdC was previously suggested to be involved in σ^G^ activity [29], we considered the possibility that the shape defect of the *ssdC* mutant could be related to the A-Q complex that spans the inner and outer forespore membranes and is required for spore shape and σ^G^ activity [34–35]. Closer examination of σ^G^ activity in the Δ*ssdC* mutant alone or in combination with an A-Q complex mutant (Δ*spoIIIAH)*, suggests that SsdC is not required for σ^G^ activity (S2 Fig). Furthermore, the forespore shape defect of A-Q complex mutants is different to that of the Δ*ssdC* mutant, in that the A-Q complex mutant forespores are distinctly smaller, and with a collapsed appearance, and all small forespores are defective in σ^G^ activity [34,36–37] (S2 Fig). These results, and others presented below, suggest that SsdC’s role in the formation of the oblong-shaped forespore is unrelated to the A-Q complex.

### SsdC forms foci in the mother cell membrane that localize toward the mother cell proximal pole of the forespore in engulfment-completed cells

**Fig 2:**
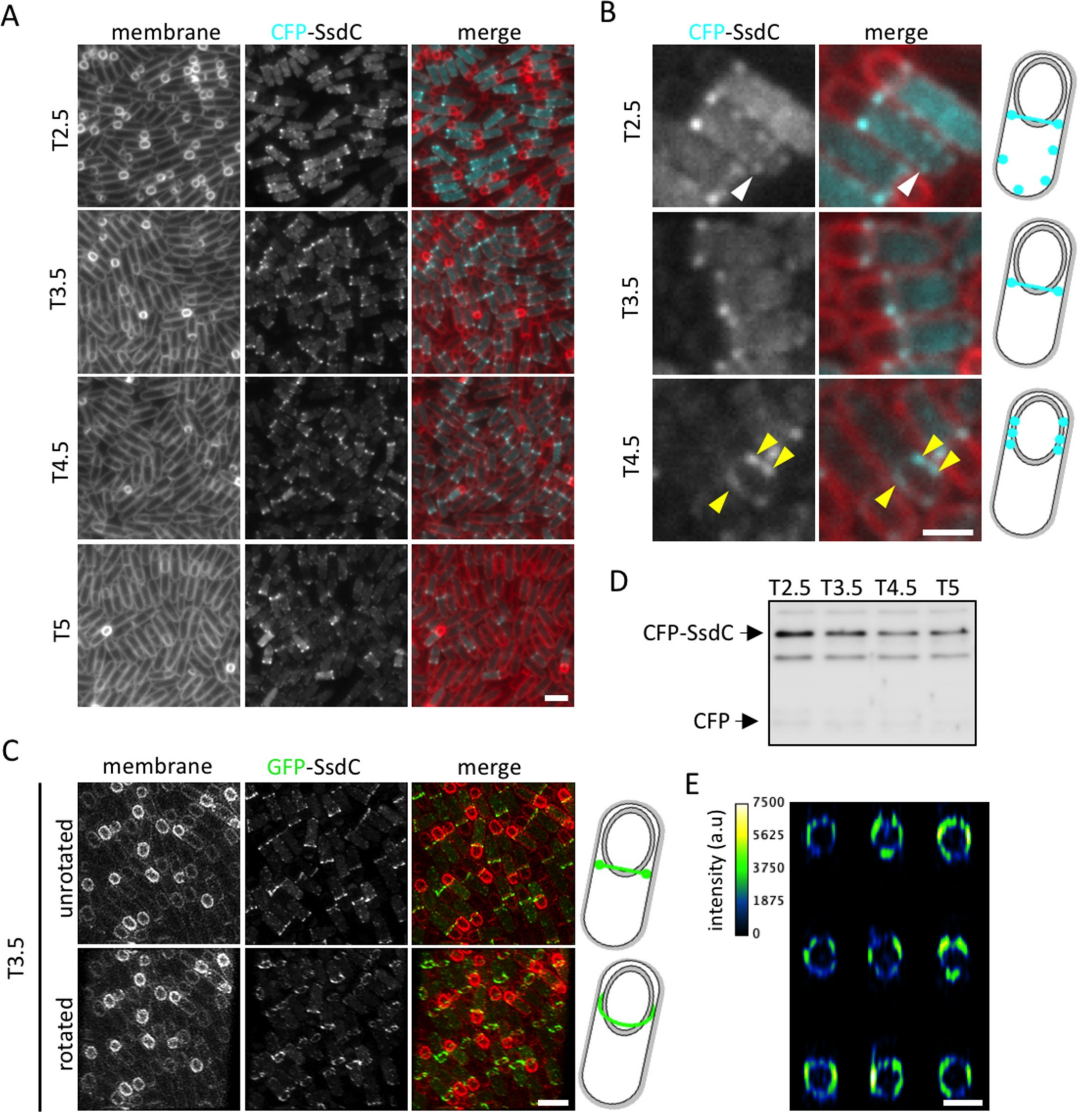
CFP-SsdC localization and ring-like structure in sporulating cells. **(A)** Fluorescence localization of CFP-SsdC (bBK20) during a sporulation time-course. CFP signal is false-coloured cyan in merged images. Cell membranes were visualised with TMA-DPH fluorescent membrane dye and are false-coloured red in merged images. Scale bar = 2 μm. **(B)** Close-up of CFP-SsdC localization at various timepoints during sporulation. Fluorescence signals are false-coloured as in (A). White arrowheads indicate CFP-SsdC foci along the mother cell membrane. Yellow arrowheads indicate CFP-SsdC foci that are dispersed around the forespore. Scale bar = 1 μm. **(C)** 3D-Structured Illumination Microscopy (3D-SIM) of GFP-SsdC (bBK21) localization at 3.5 h of sporulation (T3.5). Top panels: unrotated view; bottom panels: rotated 10–20° along z-axis. GFP signal is false-coloured green in merged images. Cell membranes were visualised with FM4-64 fluorescent membrane dye and are false-coloured red in merged images. Scale bar = 2 μm. **(D)** Immunoblot analysis of cell lysates containing CFP-SsdC (bBK20), collected during a sporulation time-course. CFP-SsdC was immunodetected using anti-GFP antibodies. The positions of CFP-SsdC and CFP are indicated (see also S1E Fig). **(E)** Fluorescence intensity plots of individual GFP-SsdC rings captured using 3D-SIM. Scale bar = 1 μm.

To investigate SsdC localization during spore morphogenesis, we generated a functional CFP-SsdC fluorescent-fusion (S1B Fig) and examined its localization during a sporulation time-course (Fig 2A and 2B). At an early stage of sporulation (T2.5), CFP-SsdC localized as foci along the mother cell membrane, or as a cytoplasmic haze. At T3.5, when most cells had completed engulfment, CFP-SsdC localized as two discrete foci, or a transverse band, at the MCP pole of the forespore. At later stages (T4.5 and T5), in most sporulating cells, the two bright foci of CFP-SsdC were less concentrated at the MCP pole of the forespore and in some cells, foci were observed around the forespore (Figs 2B and S1D). Intriguingly, unlike well-characterized mother cell membrane-associated proteins, the CFP-SsdC foci did not appear to be enriched in the engulfing membrane, but were located in the peripheral membrane. Immunoblot analysis confirmed that the majority of CFP-SsdC remained full-length and therefore reported on the localization of SsdC (Figs 2D and S1E). Importantly, unbiased analysis of CFP-SsdC localization using MicrobeJ (see Materials & Methods) recapitulated the observations described above (S1A Fig). Thus, SsdC has a dynamic localization: it initially localizes along the mother cell membrane, becomes concentrated toward the MCP pole of the forespore as bright foci, and then it disperses as foci in the peripheral mother cell membrane at later stages of development.

### SsdC foci at the mother cell proximal pole of the forespore form a ring-like structure composed of protein clusters

The striking foci formed by SsdC near the MCP pole of the forespore resembles the localization of the cell division protein FtsZ, which forms a ring-like structure that also appears as two bright foci or a band [38], but at the midcell position of vegetative cells. This specific localization of FtsZ has been shown to represent a ring-like structure at the division site [38]. Based on these similarities, we investigated if the bright foci of CFP-SsdC near the MCP pole of the forespore also represents a ring-like structure. To this end, we performed 3D-structured illumination microscopy (3D-SIM) on sporulating cells expressing a functional GFP-SsdC fluorescent-fusion (S1B Fig), which was less susceptible to photobleaching than CFP-SsdC and was thus more suitable for longer acquisition times.

GFP-SsdC had an identical localization pattern as CFP-SsdC (S1F Fig). 3D-SIM of sporulating cells at T3.5 revealed that the double foci or band localization of GFP-SsdC actually result from the projection of a ring-like pattern (Fig 2C). The GFP-SsdC ring-like structure had an average diameter of 0.87 μm *±* 0.11 μm (*±* STDEVP; n = 200), which is close in size to the width of *B*. *subtilis* cells [39]. Slicing through the radial *z*-axis of the GFP-SsdC ring-like structure showed patches of different signal intensity (Fig 2E), suggesting that the ring-like structure is composed of discontinuous clusters of SsdC. Furthermore, short time-lapse experiments suggest that SsdC clusters are dynamic, within and outside the ring-like structure (S3 Fig). Collectively, these data suggest that SsdC has a dynamic localization and forms a discontinuous ring-like structure near the mother cell proximal pole of the forespore.

### A synthetic lethal sporulation screen identifies a relationship between *ssdC* and genes involved in the spore coat

**Fig 3:**
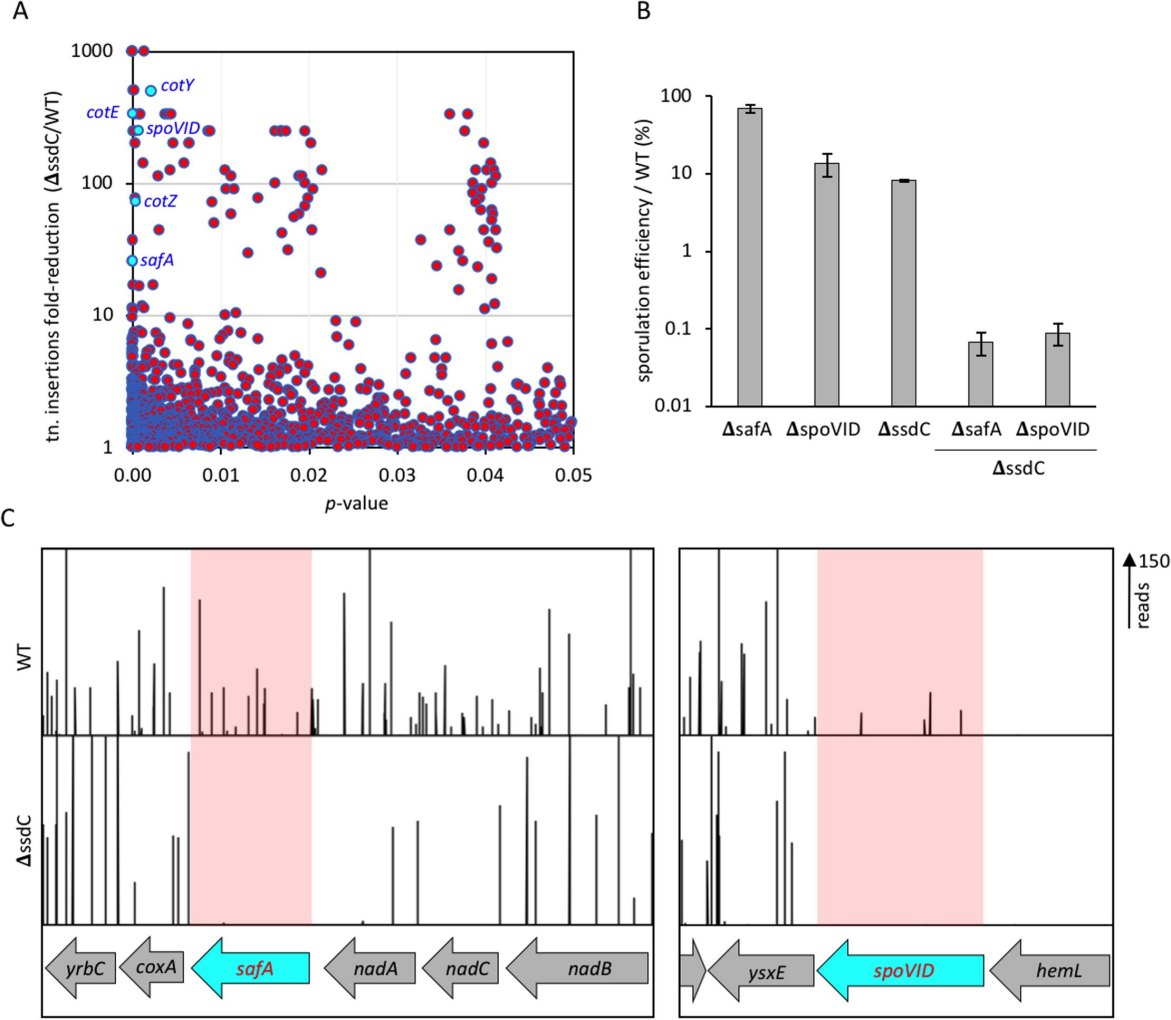
Tn-seq reveals coat genes that are important for sporulation in the absence of *ssdC*. **(A)** Scatterplot showing fold-reduction of transposon insertions in Δ*ssdC* (bCR1565) relative to wild-type (WT) cells (BDR2413), with corresponding *p*-values. Spore coat genes with high fold-reduction in Δ*ssdC* compared to WT cells and low *p*-value are labelled and coloured cyan. **(B)** Sporulation efficiency of mutant strains Δ*safA* (bBK33), Δ*spoVID* (bBK3), Δ*ssdC* (bBK28), Δ*ssdC* Δ*safA* (bBK48) and Δ*ssdC* Δ*spoVID* (bBK43) as a percentage of wild-type (BDR2413, WT). Error bars represent standard deviation of three biological replicates. **(C)** Tn-seq profiles at the *safA* and *spoVID* genomic loci of wild-type (BDR2413, WT) and Δ*ssdC* (bCR1565) cells, following 24 h of growth and sporulation in exhaustion medium. Height of vertical lines represents number of transposon-sequencing reads at each position. Shaded regions highlight the significant reduction in sequencing reads at *safA* and *spoVID* loci.

The fluorescence microscopy data indicate that SsdC is not enriched in the engulfing mother cell membrane and does not appear to become a part of the developing forespore. Yet, SsdC nonetheless affects forespore shape. To investigate the possibility that SsdC’s function might involve intermediate factors connected to the outer forespore membrane, we used transposon-sequencing (Tn-seq) [27] combined with heat-kills to screen for genes that become critical for sporulation in cells lacking SsdC. Saturating transposon libraries were constructed in the wild-type and in the Δ*ssdC* mutant (see Materials & Methods). Analysis of the transposon insertion profiles revealed a large set of genes in which insertions were underrepresented in the Δ*ssdC* mutant when compared to the wild-type (Fig 3A and S1 and S2 Tables). Surprisingly, many of the genes identified encode proteins with characterized roles in the assembly of the spore coat and crust (e.g: *safA* and *spoVID*, *cotE*, *cotZ*) (Fig 3A and 3C and S1 and S2 Tables) suggesting a genetic relationship between *ssdC* and the spore coat. Deleting most of these genes in combination with a Δ*ssdC* mutation only caused a moderate decrease in sporulation efficiency relative to the Δ*ssdC* mutant (S1 Table). By contrast, combining Δ*spoVID* or Δ*safA* with Δ*ssdC* resulted in sporulation efficiencies of 0.08% and 0.09%, respectively (Fig 3B). Since SpoVID and SafA are involved in the assembly of the inner spore coat and both mutants exhibit a strong sporulation defect when combined with the Δ*ssdC* mutant, we investigated these mutants further.

### *ssdC* and *spoVID* mutants produce irregular-shaped forespores

**Fig 4:**
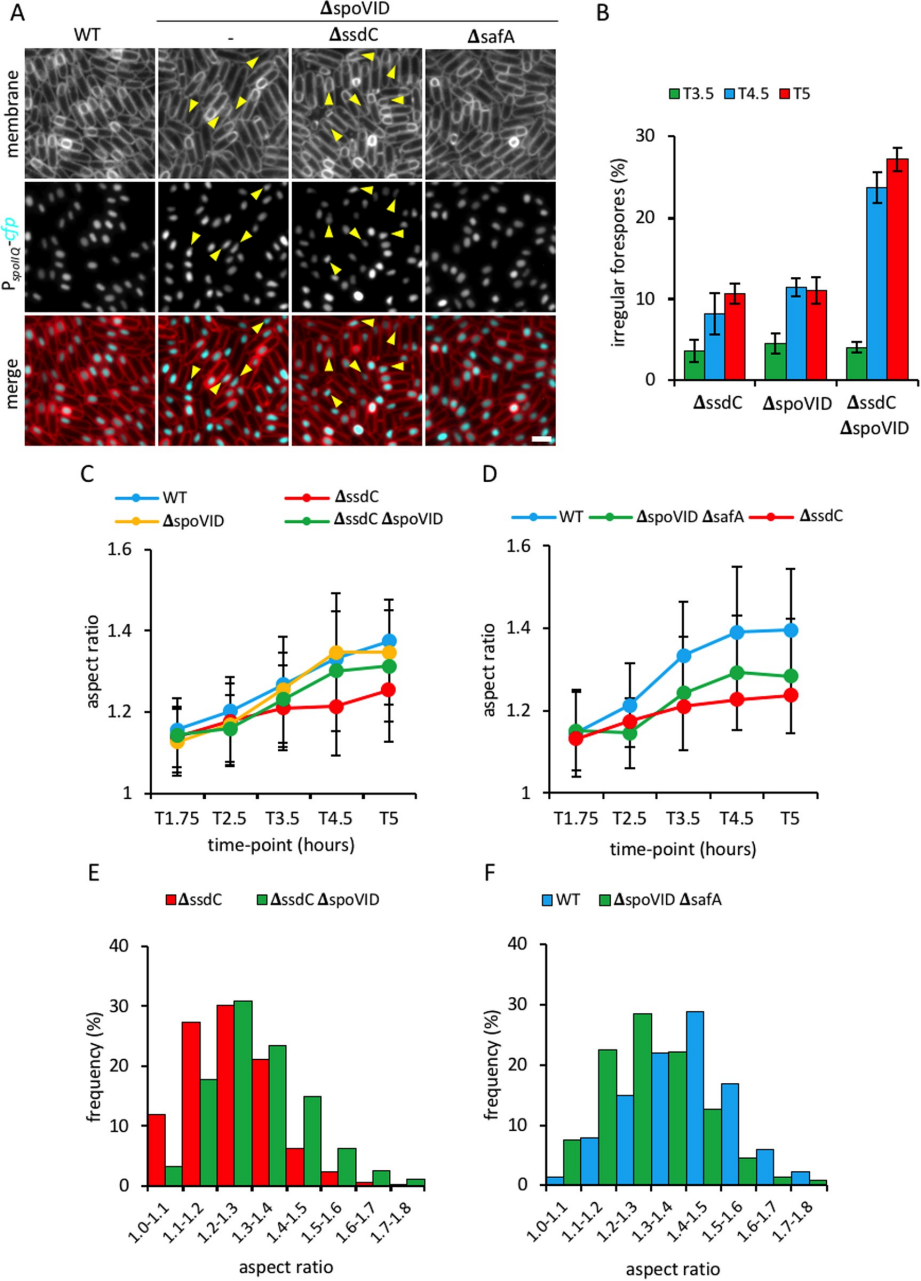
Forespore shape in *ssdC*, *spoVID* and *safA* mutants. **(A)** Forespore morphology of wild-type (WT, bBK17), Δ*spoVID* (bBK64), Δ*ssdC* Δ*spoVID* (bBK60), and Δ*spoVID* Δ*safA* (bJL196) strains at 4.5 h after onset of sporulation (T4.5). Forespore cytoplasm was visualised using a forespore reporter (P_*spoIIQ*_-*cfp*, false-coloured cyan in merged images). Cell membranes were visualised with TMA-DPH fluorescent membrane dye and are false-coloured red in merged images. Scale bar = 2 μm. **(B)** Frequency of irregularly-shaped forespores in Δ*ssdC* (bBK18), Δ*spoVID* (bBK64) and Δ*ssdC* Δ*spoVID* (bBK60) strains at T3.5 (green), T4.5 (blue) and T5 (red) of sporulation. Irregular forespores were defined as elongated and distorted in shape, as pointed out by yellow arrowheads. Error bars represent standard deviation of three biological replicates. *n* > 250 per replicate, per time-point, per strain. **(C)** Average forespore aspect ratio (± STDEVP) of wild-type (WT, bBK17, blue), Δ*ssdC* (bBK18, red), Δ*spoVID* (bBK64, yellow) and Δ*ssdC* Δ*spoVID* (bBK60, green) strains during a sporulation time-course. *n* > 500 per time-point, per strain. **(D)** Average forespore aspect ratio (± STDEVP) of wild-type (WT, bBK17, blue), Δ*ssdC* (bBK18, red) and Δ*spoVID* Δ*safA* (bJL196, green) strains during a sporulation time-course. *n* > 500 per time-point, per strain. **(E)** Frequency distribution of forespore aspect ratios of Δ*ssdC* (bBK18, red) and Δ*ssdC* Δ*spoVID* (bBK60, green) strains at 5 h after onset of sporulation (T5). *n* > 1000 per strain. **(F)** Frequency distribution of forespore aspect ratios of wild-type (WT, bBK17, blue) and Δ*spoVID* Δ*safA* (bJL196, green) strains at 5 h after onset of sporulation (T5). *n* > 1400 per strain.

To examine whether the Δ*ssdC* Δ*spoVID* and Δ*ssdC* Δ*safA* double mutants have a more pronounced forespore shape defect than the Δ*ssdC* mutant, we expressed the cyan fluorescent protein (CFP) in the forespore cytoplasm (P_*spoIIQ*_-*cfp*) and analysed the aspect ratio of individual forespores during a sporulation time-course. The average forespore aspect ratio in Δ*spoVID* and Δ*safA* single mutants increased over time in a manner that resembled the wild-type (Figs 4A, 4C, S4A and S4B). The Δ*ssdC* Δ*safA* double mutant forespores had similar aspect ratio over time to the Δ*ssdC* single mutant (S4B Fig). Surprisingly, the Δ*spoVID* mutation appeared to partially suppress the round forespore phenotype of the Δ*ssdC* mutant; the double mutant forespores developed significantly higher aspect ratios than the Δ*ssdC* mutant (*p*<0.001, Kolmogorov-Smirnov test) (Fig 4C and 4E), suggesting that the Δ*ssdC* Δ*spoVID* double mutant forespores become more elongated, rather than rounder.

Interestingly, closer inspection of the images in the Δ*spoVID* mutant revealed the presence of irregular, elongated forespores (Fig 4A) that were reminiscent of the irregular forespore phenotype observed in the Δ*ssdC* mutant (Figs 1B and 4A). The irregular forespores were separately confirmed in sporulating cells expressing a membrane bound GFP reporter (MalF^Tms^-GFP) localized in the inner forespore membrane (S4D Fig). The frequency of irregular forespores increased over time and was at least two-fold higher in the Δ*ssdC* Δ*spoVID* double mutant than the Δ*ssdC* or Δ*spoVID* mutants (Fig 4B). The Δ*ssdC* Δ*safA* double mutant had a similar frequency of irregular forespores as the Δ*ssdC* mutant (S4C Fig). The higher frequency of irregular, elongated forespores in the Δ*ssdC* Δ*spoVID* double mutant may explain its decreased sporulation efficiency. Interestingly, forespore shape in the Δ*spoVID* Δ*safA* double mutant was significantly rounder than wild-type (Fig 4A, 4D and 4F), suggesting that SpoVID and SafA are also required to maintain the shape of the developing forespore.

### SsdC requires multiple coat proteins for its localization

Both SpoVID and SafA localize to the engulfing mother cell membrane and over time surround the forespore in a process called encasement, with SpoVID being required for encasement by SafA [21]. Although SsdC does not become enriched in the engulfing membranes (Fig 2), we wondered if SsdC localization depends on SpoVID or SafA.

**Fig 5:**
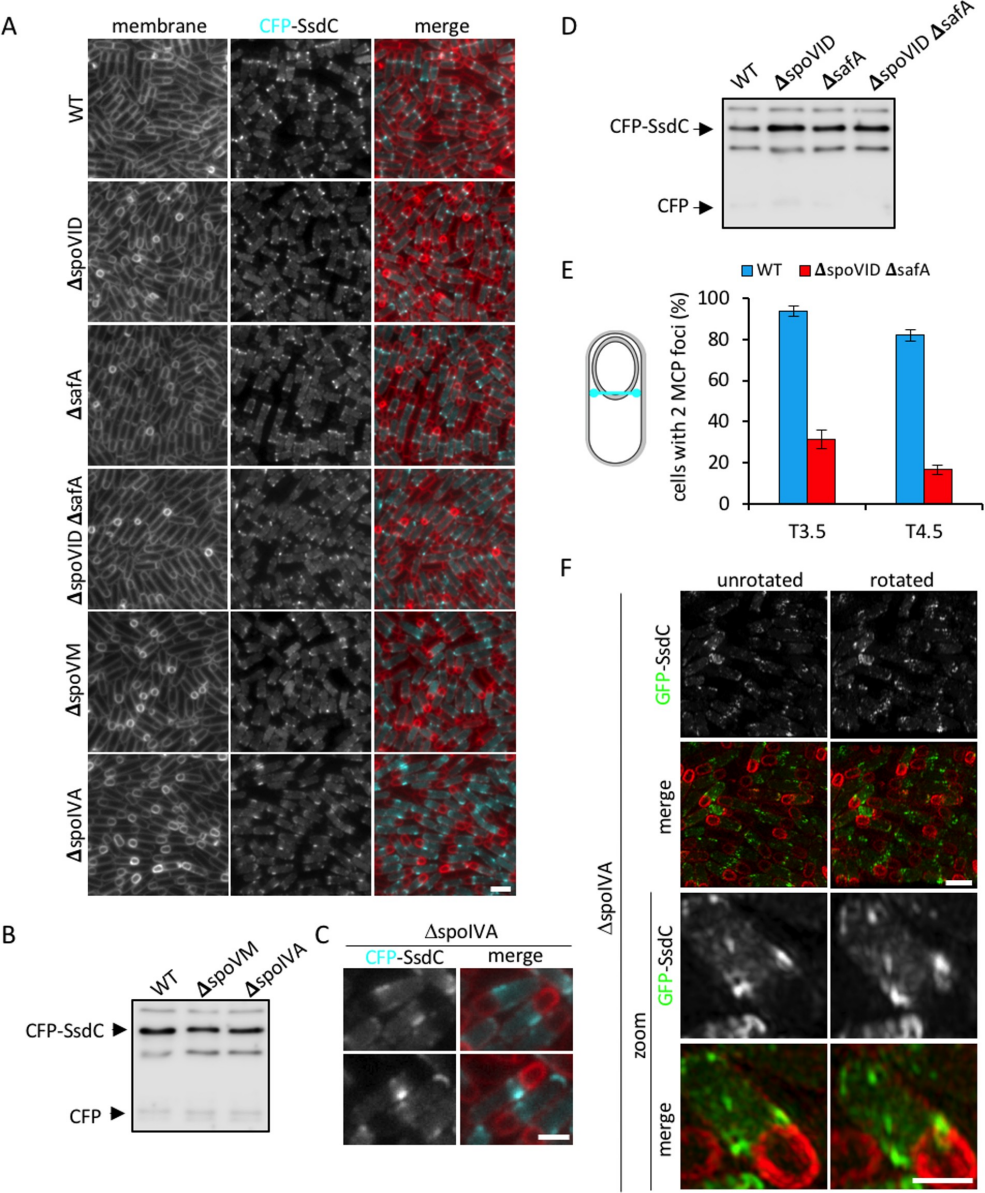
CFP-SsdC localization in coat mutants. **(A)** Fluorescence localization of CFP-SsdC in wild-type (bBK20, WT), Δ*spoVID* (bBK54), Δ*safA* (bBK56), Δ*spoVID* Δ*safA* (bJL190), Δ*spoVM* (bJL33) and Δ*spoIVA* (bJL34) mutant strains at 3.5 h after onset of sporulation (T3.5). CFP signal is false-coloured cyan in merged images. Cell membranes were visualised with TMA-DPH fluorescent membrane dye and are false-coloured red in merged images. Scale bar = 2 μm. **(B)** Immunoblot analysis of CFP-SsdC in cell lysates from wild-type (bBK20, WT), Δ*spoVM* (bJL33) and Δ*spoIVA* (bJL34) mutant strains collected 3.5 h after onset of sporulation (T3.5). CFP-SsdC was immunodetected using anti-GFP antibodies. The positions of CFP-SsdC and CFP are indicated. **(C)** Close-up of representative cells in (A), showing mislocalization of CFP-SsdC in Δ*spoIVA* mutant (bJL34). Fluorescence signals are false-coloured as in (A). Scale bars = 1 μm. **(D)** Immunoblot analysis of CFP-SsdC in cell lysates from wild-type (bBK20, WT), Δ*spoVID* (bBK54), Δ*safA* (bBK56) and Δ*spoVID* Δ*safA* (bJL190) mutant strains collected 3.5 h after onset of sporulation (T3.5). CFP-SsdC was immunodetected using anti-GFP antibodies (see also S1E Fig). The position of CFP-SsdC and CFP are indicated. **(E)** Histogram showing proportion of cells (% ± STDEV, 3 biological replicates) with two MCP CFP-SsdC foci in wild-type (bBK20, WT, blue) and Δ*spoVID* Δ*safA* (bJL190, red) cells at T3.5 and T4.5 of sporulation. *n* > 400 per replicate, per time-point, per strain. **(F)** 3D-Structured Illumination Microscopy (3D-SIM) of GFP-SsdC localization in Δ*spoIVA* (bJL40) mutant strains at 3.5 h after onset of sporulation (T3.5). An unrotated view (left panels) and rotated view 10–20° along z-axis (right panels) is shown, as well as zoom (bottom panel). Cell membranes were visualised with FM4-64 fluorescent membrane dye and are false-coloured red in merged images. Scale bars = 1 μm.

Examination of CFP-SsdC in strains lacking *spoVID* or *safA* revealed a localization pattern indistinguishable from wild-type (Fig 5A). However, in the Δ*spoVID* Δ*safA* double mutant, fewer sporulating cells had discrete CFP-SsdC foci adjacent to the MCP pole of the forespore (Fig 5E). Furthermore, MicrobeJ analysis revealed a more dispersed pattern of CFP-SsdC foci in the Δ*spoVID* Δ*safA* double mutant (S5 Fig).

Since the basement layer is required for the assembly of all coat proteins [14], including SafA and SpoVID, we investigated whether SpoIVA and SpoVM would be required for SsdC localization. Because these proteins are essential for sporulation [27], *spoIVA* and *spoVM* would not have been identified in our Δ*ssdC* Tn-seq screen. In the absence of SpoIVA, CFP-SsdC failed to form the two characteristic foci at the MCP pole of the forespore in virtually all cells (Fig 5A). Instead, CFP-SsdC resembled bands or clusters to one side of the MCP pole of the forespore, or at the forespore-free end of the sporangium (Fig 5A and 5C). Similar results were obtained using 3D-SIM (Fig 5F). In the absence of SpoVM, the frequency and intensity of CFP-SsdC foci at the MCP pole of the forespore was reduced compared to otherwise wild-type cells (Figs 5A and S6C). The mislocalization of CFP-SsdC in the Δ*spoVID* Δ*safA* double mutant and in the Δ*spoIVA* and Δ*spoVM* mutants was not due to proteolysis of CFP-SsdC, as it remained mostly full-length, with a very small degree of proteolysis, in all backgrounds examined (Fig 5B and 5D). Thus, SsdC localization depends on SpoVID and SafA, as well as SpoIVA and, to a lesser extent, SpoVM.

Reciprocally, we investigated whether SpoVID, SafA, SpoIVA and SpoVM localization depends on SsdC. For SpoVID and SpoVM, we used previously characterized SpoVID-GFP and SpoVM-GFP fluorescent fusions as the sole source of their respective proteins [21]. For SafA and SpoIVA, we constructed SafA-mYPET and mYPET-SpoIVA fluorescent fusions and analyzed them in merodiploid strains, which produced wild-type levels of spores (S6E Fig), since neither fluorescent fusion was fully functional. Localization of these coat proteins in the Δ*ssdC* mutant revealed localization patterns that were similar to wild-type sporulating cells (S6B, S6D and S6F Fig). Thus, SsdC is not required for the localization of SpoVID, SafA, SpoIVA and SpoVM, however, these basement layer and inner coat proteins play an important role in localizing SsdC.

### SsdC localization requires engulfment-induced membrane curvature and the SpoIIIAH-SpoIIQ interaction

SsdC localization as two bright foci near the MCP pole of the forespore occurs in cells that have completed engulfment (Fig 2). Thus, we investigated if this localization depends on engulfment completion. To this end, we localized CFP-SsdC in engulfment mutants (S7A Fig). Engulfment depends on the DMP complex [40–41], composed of SpoIID, SpoIIM and SpoIIP. SpoIID and SpoIIP degrade septal peptidoglycan and in their absence, the asymmetric septum remains flat [40–41]. In cells lacking either SpoIID or SpoIIP, septal PG degradation is incomplete resulting in septal membrane bulges that protrude into the mother cell cytoplasm [40–41]. In the Δ*spoIID* Δ*spoIIP* double mutant, CFP-SsdC rarely formed two foci but localized instead as puncta throughout the mother cell membrane. The localization of CFP-SsdC in the Δ*spoIID* Δ*spoIIP* double mutant was not due to proteolysis of the CFP-SsdC fusion (S7C Fig). The mislocalization of SsdC in this double mutant could be due to the mislocalization of SpoIVA and SpoVM, since localization of these proteins also depends on the engulfment-induced membrane curvature [42] (S8 Fig). Interestingly, in the Δ*spoIIP* and Δ*spoIID* single mutant, although fewer cells contained two bright CFP-SsdC foci, some cells formed two foci that were positioned either side of the membrane bulges (S7A and S7B Fig). These data suggest that SsdC localization as two foci does not require engulfment completion but requires the formation of a curved membrane that occurs when septal PG hydrolysis proceeds normally or when PG degradation is incomplete and membrane bulging occurs.

Next, we investigated whether the localization of SsdC depends on the highly-conserved SpoIIIAH-SpoIIQ protein interaction across the septal membrane, which functions as a localization hub for membrane proteins in the inner and outer forespore membranes during engulfment [35–36]. Localization of CFP-SsdC in the Δ*spoIIIAH* mutant resulted in a reduction in the number of cells with two bright foci at the MCP pole of the forespore (S7A and S7D Fig). A more dramatic reduction was observed in the Δ*spoIIQ* mutant, with even fewer cells producing two bright CFP-SsdC foci at the MCP pole of the forespore (S7A and S7D Fig). MicrobeJ analysis confirmed a more dispersed pattern of SsdC-foci localization in the Δ*spoIIIAH* and Δ*spoIIQ* mutants, compared to wild-type (S5 Fig). The localization pattern of CFP-SsdC in the Δ*spoIIIAH* or Δ*spoIIQ* mutant was not due to proteolysis of the CFP-SsdC fusion, as it remained mostly full-length, with a very small degree of proteolysis, in all strain backgrounds (S7C Fig). Notably, in the absence of SpoIIQ, both SpoVM-GFP and mYPET-SpoIVA were partially mislocalized: the fluorescent signal was no longer solely enriched around the forespore but also accumulated in the mother cell membrane (S8 Fig). Since both SpoVM and SpoIVA were partially mislocalized in the absence of SpoIIQ, we conclude that SsdC’s mislocalization in the absence SpoIIQ is due to the mislocalization of these basement layer proteins.

### The basement layer of the coat is required for forespore shape

**Fig 6:**
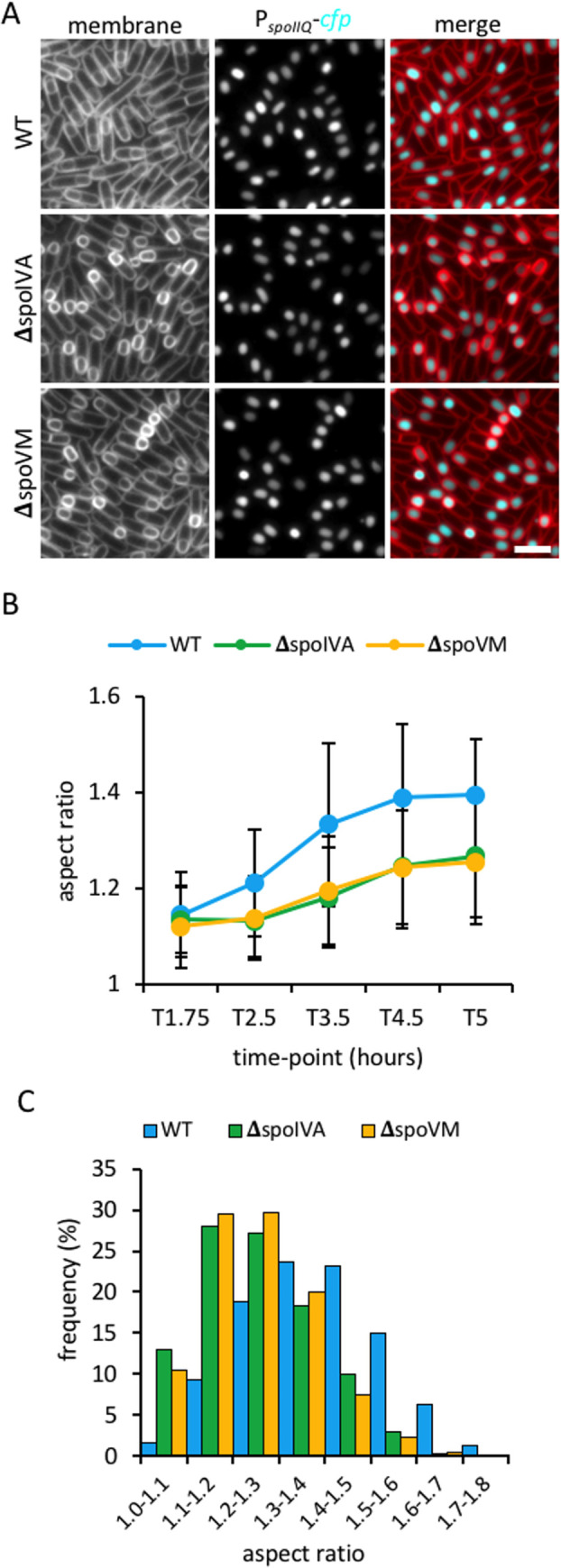
Forespore shape in *spoVM* and *spoIVA* mutants. **(A)** Forespore morphology of wild-type (WT, bBK15), Δ*spoIVA* (bJL43) and Δ*spoVM* (bJL39) strains at 4.5 h after onset of sporulation (T4.5). Forespore cytoplasm was visualised using a forespore reporter (P_*spoIIQ*_-*cfp*, false-coloured cyan in merged images). Cell membranes were visualised with TMA-DPH fluorescent membrane dye and are false-coloured red in merged images. Scale bar = 2 μm. **(B)** Average forespore aspect ratio (±STDEVP) of wild-type (WT, bBK15, blue), Δ*spoIVA* (bJL43, green) and Δ*spoVM* (bJL39, yellow) strains during a sporulation time-course. *n >* 200 per time-point, per strain. **(C)** Frequency distribution histogram of forespore aspect ratio of wild-type (WT, bBK15, blue), Δ*spoIVA* (bJL43, green) and Δ*spoVM* (bJL39, yellow) strains at T5, *n*>700.

Since SsdC localization depends on the basement layer proteins, SpoIVA and SpoVM, we wondered if basement layer mutants have a rounder forespore phenotype like the Δ*ssdC* mutant. We therefore examined the forespore aspect ratio of basement layer mutants during a sporulation time-course. This analysis revealed that Δ*spoIVA* and Δ*spoVM* forespores fail to attain the typical shape of wild-type (Fig 6A and 6B). Indeed, similar to Δ*ssdC*, the distribution of forespore aspect ratios in the Δ*spoIVA* and Δs*poVM* mutants at T5 shifted towards smaller aspect ratios (p<0.001, Kolmogorov-Smirnov test) (Fig 6C), compared to wild-type. Collectively, these results suggest that SpoIVA and SpoVM are also required for forespore oblong shape.

### SsdC influences assembly of the cortex

**Fig 7:**
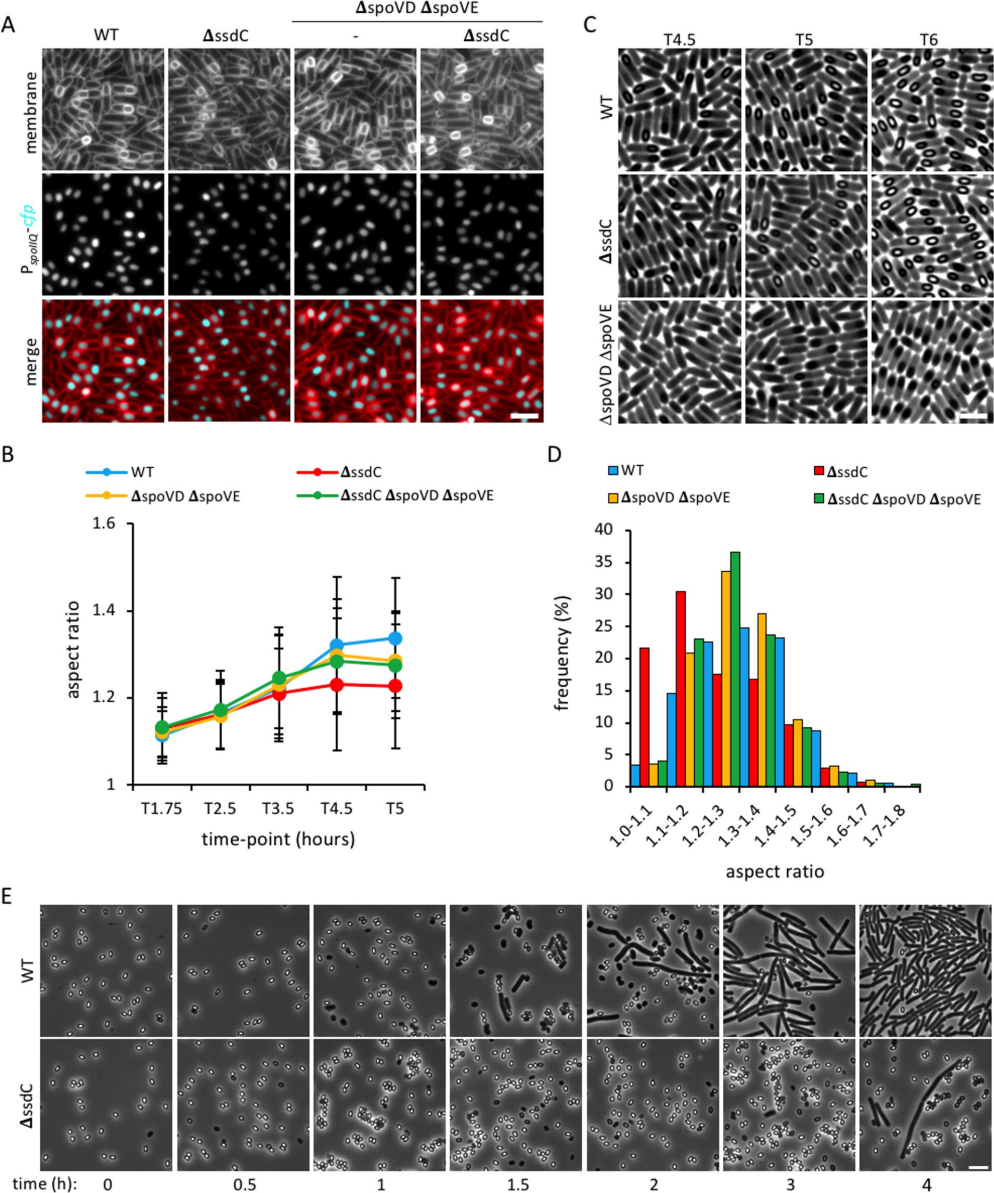
Forespore shape and germination in cortex mutants. **(A)** Forespore morphology of wild-type (WT, bBK15), Δ*ssdC* (bBK18), Δ*spoVD* Δ*spoVE* (bJL3) and Δ*ssdC* Δ*spoVD* Δ*spoVE* (bJL82) strains at 4.5 h after onset of sporulation (T4.5). Forespore cytoplasm was visualised using a forespore reporter (P_*spoIIQ*_-*cfp*, false-coloured cyan in merged images). Cell membranes were visualised with TMA-DPH fluorescent membrane dye and are false-coloured red in merged images. Scale bar = 2 μm. **(B)** Average forespore aspect ratio (± STDEVP) of wild-type (WT, bBK15, blue), Δ*ssdC* (bBK18, red), Δ*spoVD* Δ*spoVE* (bJL3, yellow) and Δ*ssdC* Δ*spoVD* Δ*spoVE* (bJL82, green) strains during a sporulation time-course. *n* > 200 per time-point, per strain. **(C)** Phase-contrast micrographs of wild-type (WT, bBK15), Δ*ssdC* (bBK18) and Δ*spoVD* Δ*spoVE* (bJL3) strains at T4.5, T5 and T6 of sporulation. Forespore cortex is visualised as phase-bright areas within the spore. Phase-dark spores in the Δ*spoVD* Δ*spoVE* (bJL3) mutant indicate absence of cortex. Scale bar = 2 μm. **(D)** Frequency distribution histogram of forespore aspect ratio of wild-type (WT, bBK15), Δ*ssdC* (bBK18), Δ*spoVD* Δ*spoVE* (bJL3) and Δ*ssdC* Δ*spoVD* Δ*spoVE* (bJL82) strains at 5 h after the onset of sporulation (T5), *n* >1000. **(E)** Phase-contrast micrographs of wild-type (bAT87, WT) and Δ*ssdC* (bJL56) spores during a germination and outgrowth time-course in nutrient-rich media (LB). Scale bar = 5 μm.

Since cells lacking SpoIVA or SpoVM do not assemble the cortex layer, we investigated if the mislocalization of SsdC, and forespore shape defect, in the Δs*poIVA* and Δs*poVM* mutant, was due to the absence of cortex. We therefore examined SsdC localization and forespore aspect ratio during a sporulation time-course in cells lacking the cortex synthases, SpoVD and SpoVE [43]. We observed no difference in the localization of CFP-SsdC in the Δ*spoVD* Δ*spoVE* double mutant compared to wild-type (S5 and S9 Figs), indicating that cortex assembly is not required for SsdC localization. Furthermore, Δ*spoVD* Δ*spoVE* mutant forespores had a similar average aspect ratio as the wild-type for most of the time-course (from T1.75 until T4.5) (Fig 7B), however, their aspect ratio decreased slightly at T5, suggesting a slight change towards a rounder shape. Therefore, the mislocalization of SsdC and rounder forespores in the absence of SpoIVA or SpoVM are likely due to deficiencies in spore coat assembly, rather than absence of cortex.

Next, we investigated whether SsdC is required for cortex assembly, which results in a change in forespore refractivity, with forespores becoming phase bright as they mature (Fig 7C) [44]. The Δ*ssdC* mutant produced refractile, phase-bright forespores that were comparable to the wild-type well into their maturation and release from the mother cell (Figs 7C and S10). Transmission electron microscopy (TEM) confirmed the presence of cortex in the Δ*ssdC* mutant (S11 Fig), however it appeared to be darker, more heterogeneous and less well defined than the wild-type. This observation led us to consider an alternate hypothesis, in which SsdC influences cortex assembly, and in its absence, SpoVE and SpoVD are improperly regulated. To investigate this, we compared the forespore shape of the Δ*ssdC* mutant in the presence and absence of cortex synthases. We found that a Δ*ssdC* Δ*spoVD* Δ*spoVE* triple mutant displayed similar forespore aspect ratios to the Δ*spoVD* Δ*spoVE* double mutant and were more oblong than the Δ*ssdC* single mutant (Fig 7A and 7B). The distribution of aspect ratios of the Δ*ssdC* Δ*spoVD* Δ*spoVE* triple mutant at T5 was significantly shifted towards larger aspect ratios compared to the Δ*ssdC* mutant (p<0.001, Kolmogorov-Smirnov test) (Fig 7D). Collectively, these results suggest that SsdC’s function in forespore shape is related to cortex synthesis and its absence results in an abnormal cortex that impacts spore shape development.

### SsdC is required for efficient spore germination

Since degradation of the cortex is a critical step in spore germination and outgrowth [15,45], we wondered whether the abnormal cortex observed in the Δ*ssdC* mutant would affect these processes. To investigate this, we incubated purified, mature spores in nutrient-rich media and monitored germination and outgrowth over time. Wild-type spores germinated within one hour, with outgrowth and exponential growth of vegetative cells evident after 1.5 hours (Fig 7E). Conversely, germination of Δ*ssdC* mutant spores was severely delayed, and exponential cell growth was not observed within 4 hours (Figs 7E and S12B). Similar results were obtained with heat-treated spores (S12A and S12B Fig), although the number of germinated colonies decreased slightly after Δ*ssdC* mutant spores were heat-treated (S12C Fig), suggesting that some spores are heat-sensitive. Our results therefore suggest that the abnormal cortex observed in the Δ*ssdC* mutant spores results in both a germination and heat-resistance defect.

## Discussion

Here, we have shown that SsdC, an orthologue of MucB / RseB from Gram-negative bacteria, functions in maintaining forespore shape in *B*. *subtilis*. In its absence, the forespore is defective in cortex assembly and fails to adopt the typical oblong shape, resulting in rounder forespores. Our data suggest that SsdC has a dynamic localization and forms an intriguing ring-like structure in the peripheral membranes of the mother cell, near the MCP pole of the forespore. The role of this structure in forespore shape is still unclear. Importantly, we identified genetic relationships between SsdC and the spore coat. Characterization of these relationships revealed that SsdC localization depends on spore coat assembly and that the spore coat also influences spore shape. Collectively, our work implicates SsdC in the development of spore shape and suggests that spore shape is dependent on proper assembly of both the cell wall cortex and spore coat. Furthermore, our data suggest functional diversification of the MucB / RseB protein domain between diderm and monoderm bacteria.

### Significance of SsdC’s localization

SsdC’s localization pattern (Fig 2) differs from well-characterized proteins expressed in the mother cell, including many coat proteins and the cortex synthesis enzymes, which become enriched in the forespore outer membrane during engulfment [24,46]. Well-known examples of this localization pattern include the membrane-anchored SpoIIIAH, SpoIVFA, and SpoIVFB proteins, which localize by diffusion-and-capture [46–48]. These proteins are inserted into the mother membrane and diffuse along the membrane until they are captured and enriched in the forespore outer membrane by other proteins. For example, in the case of SpoIIIAH, it is captured in the forespore outer membrane by SpoIIQ expressed in the forespore [48]. Our data suggest that SsdC does not become enriched in the forespore outer membrane but accumulates as a ring-like structure in the peripheral mother cell membrane towards the MCP pole of the forespore (Fig 2). How SsdC avoids enrichment in the outer forespore membrane during engulfment is unclear. One possibility is that it accumulates in the engulfing membrane but is then degraded. Alternatively, it may simply lack a localization determinant in the engulfing membrane. Nonetheless, our transposon-sequencing data and SsdC’s localization dependency on SpoIVA, SpoVM, SafA and SpoVID suggests an intimate connection between SsdC and the assembly of the spore coat (Figs 3 and 5). The complete mislocalization of SsdC in the absence of SpoIVA is not surprising (Fig 6), since SpoIVA assembles the basement layer of the spore coat and plays a fundamental role in the assembly of the coat and cortex [22]. The almost complete mislocalization of SsdC in the absence of both SpoVID and SafA (Fig 5), however, suggests that SsdC specifically requires encasement of the inner coat around the spore. Consistent with this idea, SsdC localization as a ring and its subsequent dispersal in the peripheral mother cell membrane that is adjacent to the spore envelope as it matures, coincides with the timing of the encasement of the inner coat [24]. It is tempting to speculate that the specific localization of SsdC near the MCP pole of the forespore, as opposed to the mother-cell distal pole, is related to encasement of the forespore by the inner coat, which is initiated from the MCP pole of the forespore [24].

The ring-like structure formed by SsdC is reminiscent of those formed by the cytoskeletal proteins, FtsZ and MreB [38, 49–50]. These proteins function as dynamic scaffolds in cell envelope remodeling during cell division and growth, by recruiting and/or regulating a number of proteins required for these processes [51]. Unlike FtsZ and MreB, which are cytosolic and hydrolyze GTP and ATP, respectively, to polymerize into dynamic filaments on the inner leaflet of the cytoplasmic membrane [51], SsdC is membrane anchored, with the MucB / RseB-like domain facing the extracellular space, and to our knowledge does not possess a GTPase or ATPase domain. Thus, we favor the hypothesis that the ring-like structure of SsdC reflects a distinct molecular feature of how the multi-layered spore envelope is assembled, rather than SsdC acting as a scaffold in spore shape determination. Interestingly, the localization of SsdC is somewhat reminiscent of the localization of the coat proteins, YheD and YutH. Both YheD and YutH were shown to form two foci (likely rings) at the MCP pole of the forespore and depend on SpoIVA for their localization [52–53]. However, unlike SsdC, YheD and YutH eventually encase the entire forespore to make a full shell of protein similar to other coat proteins [52]. Thus, although SsdC depends on coat assembly for its localization, it may not be a bona fide coat protein.

### Role of SsdC and the relationship between spore shape and its envelope

Our data suggest that spore shape depends on the basement layer (Fig 6), inner coat (Fig 4) and properly assembled cortex (Figs 7 and S11). How each layer contributes to shape is not yet clear, but likely depends on their composition, proteins in the coat, and peptidoglycan in the cortex. Interestingly, Atomic Force Microscopy (AFM) on spore coat mutants also suggests that the coat is important for shape [54]. It was shown that cells lacking SpoVID produce elongated, irregularly-shaped spores that are reminiscent of those we observed by fluorescence microscopy (Fig 4A) [54]. Future work combining AFM with nanoindentation approaches in spore envelope mutants may reveal the biomechanical properties that each spore envelope layer contributes to shape [55].

Whether, or not, the spore shape defects in the different coat mutants are the result of SsdC mislocalization, is difficult to discern. As a case in point, the Δ*spoVM* mutant produces significantly rounder spores (Fig 6), even though SsdC remains mostly localized (Fig 5). The *spoVM* mutant shape defect is presumably due to improper encasement by coat proteins in the absence of *spoVM* [24], rather than SsdC mislocalization. Thus, our data suggest that multiple pathways contribute to spore shape—one that is dependent on SsdC and proper cortex assembly, and another that is dependent on the spore coat, on which SsdC depends for localization.

**Fig 8:**
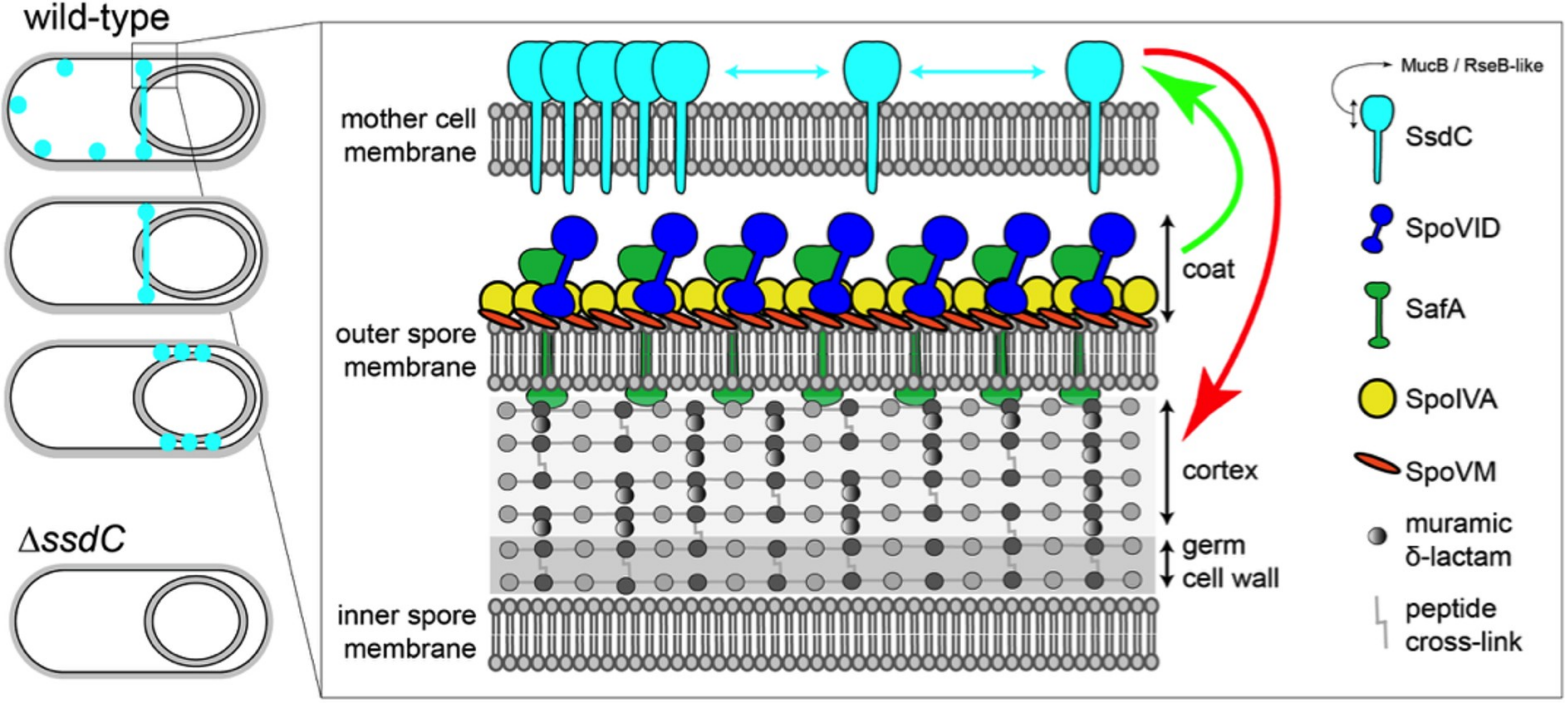
Model illustrating the relationship of SsdC with the spore coat and cortex. Schematic representation of SsdC localization and its relationship to the spore cortex and coat. The green arrow represents the localization relationship between SsdC and the coat, and the red arrow represents the shape relationship between SsdC and the cortex. We hypothesize that SsdC influences the assembly of the cortex through the spore coat and may act to coordinate the assembly of these two spore envelope layers. In this model, SafA crosses the spore outer membrane. Although recent data suggests that SafA can interact with cortex peptidoglycan in mature spores and efficient localization of SafA depends on cortex synthesis [56], it remains unclear if SafA actually transverses the membrane to do so, or if the forespore outer membrane becomes permeable, or disappears, as the spores mature.

An important question that remains unresolved is how the assembly of the coat and cortex are coordinated [14]. Some lines of evidence support the idea that they depend on each other. Mutagenesis studies on *spoIVA* suggest that its role in cortex and coat formation may not be genetically separable and that coat and cortex formation are interdependent [19]. Another piece of evidence relates to encasement by SafA, that not only depends on SpoVID but also on the cortex [56]. It has been shown that encasement by SafA is defective when its peptidoglycan-binding LysM domain is mutated, or when cortex synthesis is abolished [56]. We hypothesize that the function of SsdC in spore shape likely relates to the coordination of the assembly of coat and cortex (Fig 8). Several observations we made are consistent with this hypothesis. First, our Tn-seq data identify a relationship between *ssdC* and the coat genes (Fig 3 and S1 and S2 Tables). Second, SsdC localization depends on the basement layer and the inner coat (Fig 5). Third, we observed a higher proportion of irregularly shaped forespores in the *ssdC spoVID* double mutant, suggesting that compromised coat encasement in the absence of *ssdC* leads to a stronger effect on spore shape (Fig 4). Finally, we observed that the *ssdC* mutant shape defect is only apparent if the cortex is present (Fig 7). This suggests that assembling the cortex in the absence of SsdC causes a shape defect. That SsdC’s localization depends on the coat and its mutant shape defect depends on the cortex, suggests that it may function to coordinate assembly of the spore coat with the cortex (Fig 8).

Interestingly, our data suggest that even though the SsdC mutant spores still harbor the cortex, the cortex has a darker, less uniform appearance, suggesting a defect in cortex architecture (S11 Fig). Previous work suggests that a *cwlD* mutant also produces a darker cortex [57]. The cortex PG, although synthesized by SpoVD and SpoVE, is then subject to modification by CwlD and PdaA, required for the formation of muramic δ-lactam and the decrease in peptide cross-links [57–59]. Neither CwlD nor PdaA would have been represented in our Tn-seq screen since both genes are required for efficient germination in otherwise wild-type cells [57,59]. Loss of PdaA or CwlD does not affect heat resistance but results in reduced germination efficiency [15,57]. This is somewhat similar to what we observe with loss of SsdC, although some *ssdC* mutant spores are also defective in heat resistance (S12 Fig). Thus, perhaps SsdC coordinates the degree of cortex cross-linking, or modification, with the assembly of the coat. Future work examining cortex composition by muropeptide analysis in the absence of SsdC may reveal if the mutant is defective in cortex modification or degree of peptidoglycan cross-linking.

Another hypothesis for the role of SsdC, keeping in mind its structural similarity to MucB / RseB (Fig 1A), is that it may be part of a yet-to-be-described signaling cascade that monitors the assembly of the spore envelope in the peripheral mother cell membrane, and acts to modulate the assembly of the spore envelope using the mother cell membrane as a proxy. In the absence of SsdC, this signaling cascade may fail, leaving the spores without a shape proxy in the mother cell membrane. Given the long history of sporulation genetics, it seems unlikely that such a signaling cascade would be encoded entirely within the sporulation genes and remain unidentified. Whichever the mechanism by which SsdC controls spore shape, our data suggest that SsdC does so from a distance, anchored in the mother cell peripheral membrane. Thus, SsdC is likely part of a network of protein interactions that connect the mother cell to the spore envelope. Future work directed at identifying the SsdC protein interaction network may reveal the precise mechanism by which SsdC influences spore shape.

### Implications for understanding the evolution of the bacterial cell envelope

Phylogenetic analyses suggest that SsdC homologs are found mainly in the Bacillales and some Thermoanerobiales and Clostridiales (S13 Fig). However, the weak sequence identity but strong secondary structure similarity of the SsdC extracellular domain to MucB / RseB suggests functional diversification of this domain between diderm bacteria and monoderm Firmicutes (Fig 1A). Consistent with functional diversification of this domain, we failed to identify conserved residues between SsdC and RseB that contribute to SsdC function (six highly-conserved residues tested; S14 Fig). In diderm bacteria, MucB and RseB are core components of a signaling cascade that senses lipopolysaccharide (LPS) accumulation in the periplasm resulting from outer membrane damage, and activates the outer-membrane stress response [10,60]. Over the past decade, a debate surrounding the evolution of bacterial cell envelope has emerged from examining intriguing diderm bacteria that exist in the predominantly monoderm phylum of the Firmicutes. At the center of this debate are endospore-forming and non-endospore forming diderm Firmicutes that assemble LPS in their outer membrane [7–9]. It remains open to discussion whether diderm Firmicutes evolved from a monoderm endospore-forming ancestor by retention of the outer spore membrane during spore germination, or whether monoderm Firmicutes evolved from a diderm endospore-forming ancestor that accidentally and repeatedly lost the outer membrane [6–9]. The presence of SsdC, a MucB / RseB orthologue in monoderm endospore-formers, raises questions about the shared evolutionary past of monoderm and diderm bacteria and how proteins evolve to execute different functions in different organisms. Under the hypothesis of a sporulating diderm ancestor of the Firmicutes, SsdC homologs in Bacillales might be remnants of MucB / RseB with altered function after loss of the outer membrane. Furthermore, while future structural studies could reveal the extent of the similarity between MucB / RseB and SsdC, the weak sequence identity indicates the importance of the evolutionary events that MucB / RseB had undergone to adapt to the new function.

## Materials and methods

### General methods

All *B*. *subtilis* strains were derived from the prototrophic strain 168 [61]. Sporulation was induced by resuspension at 37°C according to the method of Sterlini-Mandelstam [62] or by exhaustion in supplemented Difco Sporulation Medium (DSM) [63] [8 g/L bacto nutrient broth (Difco), 0.1% (w/v) KCl, 1 mM MgSO_4_, 0.5 mM NaOH, 1 mM Ca(NO_3_)_2_, 0.01 mM MnCl_2_, 0.001 mM FeSO_4_] [29]. Sporulation efficiency was determined in 24 to 30-h cultures as the total number of heat-resistant (80°C for 20 min) CFUs compared with wild-type heat-resistant CFUs. Mutants for the validation of Tn-seq hits were obtained from the *B*. *subtilis* Single Gene Deletion Library (Addgene) [64]. Tables of strains (S3 Table), plasmids (S4 Table) and oligonucleotide primers (S5 Table) and descriptions of plasmid construction can be found in S1 Methods.

### Transposon-sequencing

Transposon insertion sequencing (Tn-seq) was performed on wild-type (BDR2413) and Δ*ssdC* libraries (BCR1565) as described previously [27]. Approximately, 750,000 transformants were pooled, aliquoted, and frozen. An aliquot was thawed, washed in DSM, and diluted into 50 ml DSM at an OD_600_ of 0.05. Samples were harvested 24 h later (T24). The T24 samples were incubated at 80°C for 20 min, and plated on LB agar. ~750,000 colonies from germinated spores from each sample were pooled. Genomic DNA was extracted from both samples and digested with *Mme*I, followed by adapter ligation. Transposon-chromosome junctions were amplified in 16 PCR cycles. PCR products were gel-purified and sequenced on the Illumina HiSeq platform using TruSeq reagents (Tufts University TUCF Genomics facility). Raw sequence reads are available in the Sequence Read Archive (Accession: SRP066259). Reads were mapped to the *B*. *subtilis* 168 genome (NCBI NC_000964.3), tallied at each TA site, and genes in which reads were statistically underrepresented were identified using the Mann Whitney U test. Visual inspection of transposon insertion profiles was performed with the Sanger Artemis Genome Browser and Annotation tool.

### Immunoblot analysis

Whole-cell lysates from sporulating cells were prepared as previously described [36]. Samples were heated for 15 min at 50°C prior to loading. Equivalent loading was based on OD_600_ at the time of harvest. Samples were separated on a 12.5% polyacrylamide gel and transferred to a PVDF membrane. Membranes were blocked in 5% non-fat milk with 0.5% Tween-20 for 1 h. Blocked membranes were probed with anti-GFP (1:5000) (BioRad), anti-His (1:4000) (Genscript), anti-SpoIIIAG [34] or anti-FtsZ [38] primary antibodies diluted into PBS with 5% non-fat milk (w/v) with 0.05% Tween-20 at 4°C overnight. Primary antibodies were detected with horseradish-peroxidase conjugated anti-mouse or anti-rabbit antibodies (BioRad) and detected with Western Lightning ECL reagent as described by the manufacturer.

### Protease accessibility assay

Protease susceptibility assays were performed in sporulating cells lacking the SpoIIQ (Q) protein (strain bHC70) to ensure that the membrane proteins present in the inner and outer forespore membranes would not be artificially inaccessible because of protoplast engulfment [65]. Twenty-five milliliters of sporulating cells (induced by resuspension) were harvested by centrifugation at hour 2.5 after the onset of sporulation, washed, and resuspended in 2 mL 1X SMM buffer (0.5 M sucrose, 20 mM MgCl_2_, 20 mM maleic acid, pH 6.5). The cells were protoplasted by lysozyme (5 mg/mL final concentration) for 10 min with slow agitation. The protoplasts were harvested by centrifugation and resuspended in 1 mL of 1X SMM. Protoplasts (100 μL) were incubated with trypsin (30 μg/mL final concentration) (Worthington), trypsin and Triton X-100 (2% final concentration), or 1X SMM for 2 h at 30°C. Reactions were terminated by the addition of 100 μL of 2X SDS-sample buffer and incubation for 5 min at 95°C. Five microliters from each reaction were analyzed by immunoblot.

### Fluorescence microscopy

Live-cell fluorescence imaging was performed by placing cells on a 2% (w/v) agarose pad prepared in resuspension medium and set using a gene frame (Bio-Rad). When sporulating cells reached the desired time-point, 200 μL of the culture was pelleted by centrifugation, and then resuspended in 10 μL of resuspension medium containing the the membrane dye TMA-DPH (1-(4-trimethylammoniumphenyl)-6-phenyl-1,3,5-hexatriene *p*-toluenesulfonate) (0.05 mM) or FM 4–64 (*N*-(3-Triethylammoniumpropyl)-4-(6-(4-(Diethylamino) Phenyl) Hexatrienyl) Pyridinium Dibromide) (0.67 μg/μL). After gentle vortexing, 2 μL of the cell suspension was spread on the agarose pad, and a coverslip was placed on top of the gene frame. Cells were imaged by standard epifluorescence using a Zeiss Axioplan 2 Microscope equipped with 100x objective N/A 1.4. A DAPI filter was used to excite the TMA-DPH membrane dye with an exposure time of 400 ms. CFP, GFP, and YFP filters were used with exposure times of 600 ms, 800 ms, and 1000 ms, respectively. 3D-structured illumination microscopy was performed using the DeltaVision OMX SR microscope equipped with Olympus PlanApo N 60x objective lens N/A 1.42. The 1.515 immersion oil was selected after calculating the refractive index using softWoRx software. The mCherry/A568 filter was used with an exposure time of 15–20 ms and 10–15% intensity (%T) to excite the FM 4–64 dye. A GFP/A488 filter was also used with an exposure time of 15–20 ms and 10–15% intensity (%T).

### Image analysis and statistics

Microscopy images were processed by adjusting the brightness and contrast using the Fiji software. 3D images were viewed and processed using Imaris software. The MicrobeJ plugin [33] designed for the Fiji software was also used to analyse forespore shape and detect the subcellular localization of CFP-SsdC foci.

To perform quantitative analysis of forespore shape using MicrobeJ, image background was first subtracted (Process > Subtract Background) to avoid false-positive detection of the fluorescent signal. Next, the “Bacteria” tab on MicrobeJ was set to “Smoothed” to detect the outline of CFP signal from the forespores. Three parameters: “Exclude on Edges”, “Shape descriptors”, and “Segmentation” were checked. The generated CFP outlines were further refined by setting the shape descriptors (area, length, width) for each time-point corresponding to the outlines of individual forespores. The manual editing tool was also used to resolve unprocessed forespores. Results of the analyses, such as length, width, and aspect ratio, were exported to Microsoft Excel to generate the figures. A non-parametric Kolmogorov-Smirnov test was used to compare the aspect ratio distributions between populations of wild-type and mutant sporulating cells.

To detect the subcellular localization of CFP-SsdC foci using MicrobeJ, the brightfield image was set to Channel 1 and the image with the CFP signal to Channel 2. These channels were then merged into one image. For Channel 1 of the image with “Bright” background, the “Bacteria” tab was set to “Fit Shape” and “Rod-Shaped” to detect the bacteria in the brightfield image. Five parameters: “Exclude on Edges”, “Shape descriptors”, “Segmentation”, “Intensity”, and “Feature” were checked. To refine the generated bacteria outlines, the shape descriptors (area, length, and width) were set differently for each time-point corresponding to the contours of individual cells. To resolve unprocessed cells, the manual editing tool was also used. For Channel 2 of the image with “Dark” background, the “Maxima” tab was set to “Point” and “Basic”. The tolerance was set to 3, and the intensity was set from 10-max to ensure detection of CFP foci with a minimum signal intensity of 10. Three parameters: “Exclude on Edges”, “Shape descriptors”, and “Associations” were checked. Detected foci were associated with outlines of the bacteria. Raw data were displayed in a MicrobeJ results table. The “Maxima1” section of the MicrobeJ results was selected, and the icon “Subcellular Localization” was clicked. “Density Map” and “CellHistogram” were selected to display the density and proportion, respectively, of foci localising at certain positions in the cell.

### Spore germination and outgrowth

Spores from 25 mL of sporulating wild-type (bAT87) or Δ*ssdC* (bJL56) cells (induced by resuspension) were harvested by centrifugation 27 hours after the onset of sporulation. Spores were washed with 20 mL of MilliQ H_2_O and treated with lysozyme (1.2 mg/mL) at 37°C for 1 hour. SDS was added to a final concentration of 2% and spores were incubated for a further 20 min at 37°C. Spores were harvested by centrifugation and then washed seven times with 20 mL of MilliQ H_2_O.

Spore germination and outgrowth time courses were performed by inoculating 25 mL of LB media with purified spores to an initial OD_600_ of approximately 0.1. Spore suspensions were either heat-treated (80°C, 20 min) or untreated prior to incubation at 37°C with aeration. Spore germination and outgrowth were monitored over time for two biological replicates per strain by measuring optical density at 600 nm (OD_600_) and by brightfield microscopy as described above.

### Transmission electron microscopy of mature spores

Spores were pelleted in 1.5-mL Eppendorf tubes at 10,000 rpm for 3 min and placed into primary fixative, consisting of 2.5% glutaraldehyde in 0.1 M sodium cacodylate buffer, for 2 h at room temperature. The spores were rinsed in fresh sodium cacodylate buffer three times for 15 min each. Secondary fixation was performed using 1% osmium tetroxide and 1.5% potassium ferricyanide in cacodylate buffer for 1 h at room temperature. The tissues were rinsed in three washes of Milli-Q water for 15 min each. The fixed spore pellets were dehydrated by incubating in increasing concentrations of ethanol for 15 min, consisting of 30, 50, 70, 90 and 100% ethanol. Dehydrated spore pellets were incubated in a mixture of LR White resin and ethanol at a ratio of 1:1 for 6 h at room temperature, followed by a 2:1 LR White/ethanol mixture overnight. Spore pellets were incubated in 100% LR White resin for 6 hours, followed by another 100% resin change overnight. The spore pellets were then placed into gelatin capsules in 100% resin and the resin polymerised for 24 h in an oven at 60°C. Resin embedded tissue was sectioned with a Diatome diamond knife using a Leica UCS ultramicrotome. Sections of thickness 70–90 nm were collected onto formvar-coated 100 mesh copper grids and stained sequentially with 1% uranyl acetate for 10 min and lead citrate for 5 min. The sections were imaged in a JEOL 1400+ transmission electron microscope at 80kV, and images captured with a digital camera at a resolution of 2K x 2K.

### Phylogenetic analyses

We assembled a local databank of Firmicutes by selecting one proteome per genus. Proteome selection was realized considering genomes characteristics such as assembly level and category. The assembled databank contains 497 genomes of Firmicutes, including: 4 Acidaminococcales; 120 Bacillales; 193 Clostridiales; 26 Erysipelotrichales; 11 Halanaerobiales; 40 Lactobacillales; 31 Limnochordales; 6 NA; 1 Natranaerobiales; 18 Selenomonadales; 21 Thermoanaerobacterales; 16 Tissierellales; 10 Veillonellales. In order to build a reference phylogeny, exhaustive HMM-based homology searches (with the option—cut_ga) were carried out by using HMM profiles of bacterial ribosomal proteins from the Pfam 29.0 database [66] as queries on the Firmicutes databank using the HMMER-3.1b2 package [67]. The conserved ribosomal proteins were aligned with MAFFT-v7.407 [68] with the auto option and trimmed using BMGE-1.1 [69]. The resulting trimmed alignments were concatenated into a supermatrix (497 taxa and 3,776 amino acid positions). A maximum likelihood tree was generated using IQTREE-1.6.3 [70], under the TEST option with 1000 ultrafast bootstrap replicates. Homology searches were performed using HMMSEARCH, from the HMMER-3.1b2 package to screen all the proteomes in the databank for the presence of Spo0A and SsdC. For Spo0A we used the pfam domain PF08769.11 (Spo0A_C) and the—cut_ga option in the HMMER package. For SsdC, we used the sequence identified in *Bacillus subtilis* as seed to query the Firmicutes databank using BLASTP [71]. Then we used the hits retrieved by BLASTP to build a HMM profile using hmmbuild from HMMER package. This profile was used to screen the Firmicutes databank for the presence of SsdC homologs. All hits were kept and manually curated using phylogeny, domains and synteny in order to discard false positives. All SsdC were then aligned using MAFFT, trimmed with BMGE using the BLOSUM30 substitution matrix to select unambiguously aligned positions. A maximum likelihood tree was then generated using IQTREEunder the TEST option with 1000 ultrafast bootstrap replicates. All trees were annotated using IToL [72].

## Supporting information

S1 Fig Subcellular distribution of CFP-SsdC fluorescence and validation of SsdC fluorescent-fusions. **(A)** Density maps of CFP-SsdC subcellular fluorescence localization in wild-type (bBK20) cells during a sporulation time-course (*n* > 400 per time-point). Warmer colours indicate higher density. Although sporulation by resuspension is not perfectly synchronous, many cells are at a similar stage of sporulation at each time-point. Thus, the position of foci relative to the sporangium at a population level at the different time-points provides an overview of how CFP-SsdC foci are changing position at different stages of development. Although this does not take the location of the forespore into account, since it resides at either pole of the sporangium, we can estimate its position within the sporangium relative to the CFP-SsdC foci. **(B)** Average sporulation efficiency (±STDEV, *n* = 3) of Δ*ssdC* (bBK3) in the presence and absence of CFP-SsdC (bBK20) and GFP-SsdC (bBK21), relative to wild-type (bDR2413). CFP-SsdC and GFP-SsdC complemented the Δ*ssdC* phenotype and restored sporulation efficiency to 87% and 83% of wild-type, respectively. **(C)** Average sporulation efficiency (±STDEV of three biological replicates) of Δ*ssdC* (bBK3) in the presence and absence SsdC-His6 (bHC45) relative to wild-type (bDR2413). SsdC-His6 complemented the Δ*ssdC* phenotype and restored sporulation efficiency to 81% of wild-type. **(D)** Close-up of CFP-SsdC localization in wild-type cells (bBK20) around the spore at T5. CFP signal is false-coloured cyan in merged images. Cell membranes were visualised with TMA-DPH fluorescent membrane dye and are false-coloured red in merged images. Scale bar = 1 μm. **(E)** Immunoblot analysis of CFP-SsdC in otherwise wild-type (bBK20, WT) or mutant strains. CFP-SsdC was immunodetected using anti-GFP antibodies. Bands present in strains containing CFP-SsdC but not present in Δ*ssdC* (bBK3) correspond to CFP-SsdC and a CFP degradation product. The positions of CFP-SsdC and CFP are indicated by the arrow. From left to right, immunoblots are presented in Figs 2D, 5B, 6C and S7C, respectively. **(F)** Fluorescence localization of GFP-SsdC (bBK21) during a sporulation time-course. GFP signal is false-coloured green in merged images. Cell membranes were visualised with TMA-DPH fluorescent membrane dye and are false-coloured red in merged images. Scale bar = 2 μm.
(JPG)

S2 Fig SsdC is not required for σ^G^ activity. Fluorescence microscopy of σ^G^ activity in wild-type (bKH21, WT), Δ*ssdC* (bJL66), Δ*spoIIIAH* (bKH23), Δ*ssdC* Δ*spoIIIAH* (bJL178) and Δ*gerM* Δ*spoIIIAH* (bJL179). σ^G^ activity was visualised using a σ^G^ -dependent fluorescent transcriptional reporter (P_*sspB*_-*cfp*, false-coloured cyan in merged images) at T4.5 of sporulation. σ^G^-activity is not reduced in Δ*ssdC* or Δ*ssdC* Δ*spoIIIAH* mutant strains, relative to the wild-type or Δ*spoIIIAH* mutant, respectively. As a control, and consistent with previous work [36], the Δ*gerM* Δ*spoIIIAH* mutant strain has reduced σ^G^-activity relative to the Δ*spoIIIAH* mutant. Cell membranes were visualised with TMA-DPH fluorescent membrane dye and are false-coloured red in merged images. Scale bar = 2 μm. Sporulation efficiency of mutant strains relative to WT are shown on the right (n = 2).
(JPG)

S3 Fig Time-lapse microscopy of GFP-SsdC suggests SsdC is dynamic. **(A)** 3D-Structured Illumination Microscopy (3D-SIM) time-lapse of GFP-SsdC localization in wild-type (bBK21). Three examples are shown (a, b & c). GFP signal is false-coloured green in merged images. Yellow arrowheads point to regions of the cell where GFP-SsdC localization changes over the course of the time-lapse. Scale bar = 1 μm. **(B)** Fluorescence intensity plots of GFP-SsdC rings captured from the same cells above, highlighting changes in GFP-SsdC localization within the ring-like structure over the course of the time-lapse. Scale bar = 1 μm.
(JPG)

S4 Fig Irregular forespore morphology in the absence of both SsdC and SpoVID and spore shape measurements in the absence of SafA. **(A)** Forespore morphology of Δ*safA* (bBK66) and Δ*safA* Δ*ssdC* (bBK62) strains at 4.5 h after onset of sporulation (T4.5). Forespore cytoplasm was visualised using a forespore reporter (P_*spoIIQ*_-*cfp*, false-coloured cyan in merged images). Cell membranes were visualised with TMA-DPH fluorescent membrane dye and are false-coloured red in merged images. Scale bar = 2 μm. **(B)** Average forespore aspect ratio (± STDEVP) of wild-type (WT, bBK17, blue), Δ*spoVID* (bBK18, red), Δ*safA* (bBK66, yellow) and Δ*ssdC* Δ*safA* (bBK62, green) mutant strains during a sporulation time-course. *n* > 500 per time-point, per strain. **(C)** Histogram showing proportion of sporulating cells (% ± STDEV of three biological replicates) of irregularly-shaped forespores shape in wild-type (WT, bBK17), Δ*ssdC* (bBK18), Δ*safA* (bBK66) and Δ*ssdC* Δ*safA* (bBK62) mutant strains at 3.5 (T3.5, green), 4.5 (T4.5, blue) and 5 h (T5, red) after the onset of sporulation. Irregular forespores were defined as elongated and distorted in shape. *n* > 250 per replicate, per time-point, per strain. **(D)** Forespore morphology of wild-type (WT, bJL78), Δ*ssdC* (bJL79), Δ*spoVID* (bJL193), Δ*safA* (bJL199), Δ*ssdC* Δ*spoVID* (bJL80) and Δ*safA* Δ*ssdC* (bJL81) strains harbouring a MalF^Tms^-GFP inner spore membrane reporter at 3.5 (T3.5) and 4.5 h (T4.5) after onset of sporulation. GFP signal is false-coloured green in merged images. Cell membranes were visualised with TMA-DPH fluorescent membrane dye and are false-coloured red in merged images. Scale bars = 2 μm.
(JPG)

S5 Fig CFP-SsdC subcellular fluorescence localization in different mutants. Density maps and histogram plots of CFP-SsdC subcellular fluorescence localization (*n* > 400 per strain) at 3.5 h after the onset of sporulation (T3.5) in wild-type (bBK20), Δ*spoVID* Δ*safA* (bJL190), Δ*spoIIIAH* (bBK52), Δ*spoIIQ* (bJL175), Δ*spoVM* (bJL33) and Δ*spoVD* Δ*spoVE* (bHC99). In the density maps warmer colours indicate higher density. In the histogram plots, the width of each bar indicates the frequency of localization at that specific subcellular location.
(JPG)

S6 Fig Localization of SpoVID-GFP, SafA-mYPET, SpoVM-GFP and mYPET-SpoIVA in the absence of SsdC. **(A)** Fluorescence localization of SpoVID-GFP in wild-type (bJL12), Δ*ssdC* (bJL10), Δ*safA* (bJL158) and Δ*ssdC* Δ*safA* (bJL162) mutant strains at 3.5 h after onset of sporulation (T3.5). **(B)** Fluorescence localization of SafA-mYPET in wild-type (bJL13, WT), Δ*ssdC* (bJL35), Δ*spoVID* (bJL159) and Δ*ssdC* Δ*spoVID* (bJL160) mutant strains at 3.5 h after onset of sporulation (T3.5). mYPET and GFP signals are false-coloured green in merged images. Cell membranes were visualised with TMA-DPH fluorescent membrane dye and are false-coloured red in merged images. Scale bars = 2 μm. **(C)** Histogram showing proportion of sporulating cells (% ± STDEV, 3 biological replicates) with two MCP CFP-SsdC foci in wild-type (bBK20, WT, blue) and Δ*spoVM* (bJL33, red) cells at T3.5 and T4.5 of sporulation. *n* > 400 per replicate, per time-point, per strain. **(D)** Fluorescence localization of SpoVM-GFP in wild-type (bJL133) and Δ*ssdC* (bJL135) mutant strains at 3.5 h after onset of sporulation (T3.5). **(E)** Average sporulation efficiency (±STDEV, n = 3) of mYPET-SpoIVA strains in the absence (Δ*spoIVA*, bJL136) or presence of *spoIVA* (WT, bJL129), relative to wild-type (bDR2413). The Δ*spoIVA* strain (bJL59) does not produce heat-resistant spores. mYPET-SpoIVA does not complement the Δ*spoIVA* phenotype (2.4% sporulation efficiency) and was thus used in a merodiploid background (70% sporulation efficiency). **(F)** Fluorescence localization of mYPET-SpoIVA in wild-type (bJL129, WT), and Δ*ssdC* (bJL140) mutant strains at 3.5 h after onset of sporulation (T3.5). mYPET and GFP signals are false-coloured green in merged images. Cell membranes were visualised with TMA-DPH fluorescent membrane dye and are false-coloured red in merged images. Scale bars = 2 μm.
(JPG)

S7 Fig CFP-SsdC localization in the absence of the SpoIIIAH-SpoIIQ interaction and in engulfment mutants. **(A)** Fluorescence localization of CFP-SsdC in wild-type (bBK20, WT), Δ*spoIIIAH* (bBK52), Δ*spoIIQ* (bJL175), Δ*spoIIP* (bJL176), Δ*spoIID* (bBK57) and Δ*spoIID* Δ*spoIIP* (bJL177) mutant strains at 3.5 h after onset of sporulation (T3.5). CFP signal is false-coloured cyan in merged images. Cell membranes were visualised with TMA-DPH fluorescent membrane dye and are false-coloured red in merged images. Scale bar = 2 μm. **(B)** Close-up of representative cells in (A), showing CFP-SsdC foci at either side of the membrane bulge in Δ*spoIIP* (bJL176) and Δ*spoIID* (bBK57) mutants. Fluorescence signals are false-coloured as in (A). Scale bar = 1 μm. **(C)** Immunoblot analysis of CFP-SsdC in cell lysates from wild-type (bBK20, WT), Δ*spoIIIAH* (bBK52), Δ*spoIIQ* (bJL175), Δ*spoIIP* (bJL176), Δ*spoIID* (bBK57) and Δ*spoIID* Δ*spoIIP* (bJL177) mutant strains collected at T3.5. CFP-SsdC was immunodetected using anti-GFP antibodies. The positions of CFP-SsdC and CFP are indicated (see also S1E Fig). **(D)** Histogram showing proportion of cells (% ± STDEV, 3 biological replicates) with two CFP-SsdC mother-cell proximal (MCP) foci in wild-type (bBK20, WT, blue), Δ*spoIIIAH* (bBK52, green) and Δ*spoIIQ* (bJL175, red) strains. *n* > 400 per biological replicate, per time point, per strain.
(JPG)

S8 Fig SpoVM-GFP and mYPET-SpoIVA localization in the absence of SpoIIQ and in the absence of both SpoIID and SpoIIP. **(A)** Fluorescence localization of mYPET-SpoIVA in wild-type (bJL129, WT), Δ*spoIIQ* (bJL185) and Δ*spoIID* Δ*spoIIP* (bJL189) mutant strains at 3 h after onset of sporulation (T3). **(B)** Fluorescence localization of SpoVM-GFP in wild-type (bJL133, WT), Δ*spoIIQ* (bJL187) and Δ*spoIID* Δ*spoIIP* mutant strains (bJL188) at 3 h after onset of sporulation (T3.5). mYPET and GFP signals are false-coloured green in merged images. Cell membranes were visualised with TMA-DPH fluorescent membrane dye and are false-coloured red in merged images. Scale bar = 2 μm.
(JPG)

S9 Fig SpoVD and SpoVE are not required for SsdC localization. Fluorescence localization of CFP-SsdC in wild-type (bBK20, WT) and Δ*spoVD* Δ*spoVE* (bHC99) mutant strain at 3.5 h after onset of sporulation (T3.5). CFP signal is false-coloured cyan in merged images. Cell membranes were visualised with TMA-DPH fluorescent membrane dye and are false-coloured red in merged images. Scale bar = 2 μm.
(JPG)

S10 Fig SsdC is not required for spore refractivity. Phase-contrast images of wild-type (bAT87, WT) and Δ*ssdC* (bJL56) strains during a sporulation time-course until spore maturation and release from the mother cell. Arrowheads indicate Δ*ssdC* mutant mature spores that appear rounder than WT. Scale bar = 2 μm.
(JPG)

S11 Fig Mature spores lacking SsdC display an abnormal cortex. Transmission electron microscopy images of **(A)** wild-type (bAT87, WT) and **(B)** Δ*ssdC* (bJL56) mature spores, with respective zoomed-in areas of the spore envelope. Scale bar is 200 nm. The blue bracket indicates the approximate location of the spore coat and crust, whereas the yellow bracket indicates the approximate location of the cortex.
(JPG)

S12 Fig The SsdC mutant is defective in both germination and heat-resistance. **(A)** Phase-contrast micrographs of heat-treated (80°C, 20 min) wild-type (bAT87, WT) and Δ*ssdC* (bJL56) spores during a germination and outgrowth time-course in nutrient-rich media (LB). Scale bar = 5 μm. **(B)** Optical density at 600 nm (OD_600_, ±STDEV, *n* = 2) of heat-treated (+HK) wild-type (bAT87, WT, light green) and Δ*ssdC* (bJL56, yellow) spores, and untreated (-HK) wild-type (bAT87, WT, dark green) and Δ*ssdC* (bJL56, red) spores, during a germination and outgrowth time-course in nutrient-rich media (LB). Optical density decreases as spores become phase dark and germination begins. Optical density then increases as outgrowth and vegetative cell division occur. **(C)** Average germination efficiency (*n* = 3, ± STDEV) of Δ*ssdC* mutant spores that were plated on LB-agar without heat-treatment (-HK, 7%) and with heat-treatment (+HK) (3.7%) relative to wild-type.
(JPG)

S13 Fig The mapping of SsdC and Spo0A_C on a reference phylogeny of Firmicutes and the phylogenetic tree of SsdC homologs. Among the 497 proteomes of Firmicutes, 358 have Spo0A_C homologs and probably sporulate. Within these sporulating taxa, we retrieved SsdC homologs in 114 taxa: 11 Erysipelotrichales, 4 Thermoanaerbacterales, 97 bacillales, 1 Natranaerobiales (*Natranaerobius thermophilus JW*) and 1 Clostridiales (*Dethiobacter alkaliphilus AHT 1*). No homologs were identified in Lactobacillales. The gene tree of SsdC homologs follows the species tree with a wide distribution of ssdC in Bacillales. Moreover, the presence of ssdC in *N*. *thermophilus JW* and *D*.*alkaliphilus AHT 1*, which branch at the base of the Bacilli in both SsdC gene tree and the reference tree of the Firmicutes suggests the presence of SsdC in the ancestor of the bacilli followed by its loss in Lactobacillales. **(A)** Reference phylogeny of the Firmicutes. Maximum likelihood tree based on concatenation of 29 ribosomal proteins (497 taxa, 3,776 amino acid characters). The tree was inferred with IQ-TREE 1.6.3 using the LG+I+G4 model selected under the BIC criterion. Grey dots correspond to supports higher than 80%. The scale bar corresponds to the average number of substitutions per site. The presence of Spo0A_C and SsdC is indicated in front of each tip in red and black respectively. **(B)** Phylogenetic tree of SsdC homologs retrieved in the Firmicutes databank. Maximum likelihood tree based on an alignment of 114 taxa and 244 amino acid positions. The tree was inferred with IQ-TREE using the LG+F+I+G4 model selected under BIC criterion. Node supports higher than 80% are displayed. The scale bar corresponds to the average number of substitutions per site.
(PDF)

S14 Fig Conserved residues in the SsdC RseB-like domain are not required for SsdC function. **(A)** Average sporulation efficiency (±STDEV, *n* = 2) of Δ*ssdC* (bBK3) and CFP-SsdC mutants S118A (bHC144), P174A (bHC25), P238A (bHC27), Y261A (bHC29), F267A (bHC31) and E272A (bHC33), relative to CFP-SsdC wild-type (bBK20). Mutation of either of these residues did not negatively affect sporulation efficiency. **(B)** Fluorescence localization of CFP-SsdC wild-type (bBK20, WT), S118A (bHC144), P174A (bHC25), P238A (bHC27), Y261A (bHC29), F267A (bHC31) and E272A (bHC33) strains at 3.5 h after onset of sporulation (T3.5). CFP signal is false-coloured cyan in merged images. Cell membranes were visualised with TMA-DPH fluorescent membrane dye and are false-coloured red in merged images. Scale bar = 2 μm.
(JPG)

S1 Table Independently validated sporulation genes identified by Tn-seq. ^**a**^ Fold-difference in the number of transposon insertions: e.g. the Δ*ssdC* mutant had 333.3-fold less transposon insertions than the WT. ^**b**^ Sporulation efficiency, relative to WT, of Δ*ssdC* double mutants with the listed genes: e.g. the Δ*ssdC* Δ*cotE* double mutant had a sporulation efficiency of 0.16%. The data is average of two biological replicates. ^**c**^ Fold-change in sporulation efficiency of the Δ*ssdC* double mutants with the listed genes, relative to the Δ*ssdC* single mutant: the Δ*ssdC* Δ*cotE* mutant produced 20.1-fold less spores that the Δ*ssdC* single mutant.
(PDF)

S2 Table Spore coat and spore crust genes identified by Tn-seq. ^a^ Fold-difference in the number of transposon insertions: e.g. the Δ*ssdC* mutant had 333.3-fold less transposon insertions than the WT.
(PDF)

S3 Table *Bacillus subtilis* strains used in this study. All *Bacillus subtilis* strains used in this study.
(PDF)

S4 Table Plasmids used in this study. All plasmids used in this study.
(PDF)

S5 Table Oligonucleotide primers used in this study. *capital letters indicate restriction sites.
(PDF)

S1 Methods Details for plasmid and strain constructions in this study. All construction details for plasmids used in this study.
(PDF)
