# Supplementary figures and images for: A dynamic, ring-forming MucB / RseB-like protein influences spore shape in Bacillus subtilis
Source: PLoS Genet. 2020 Dec 14;16(12):e1009246. doi: 10.1371/journal.pgen.1009246 (PMC7769602; doi:10.1371/journal.pgen.1009246)

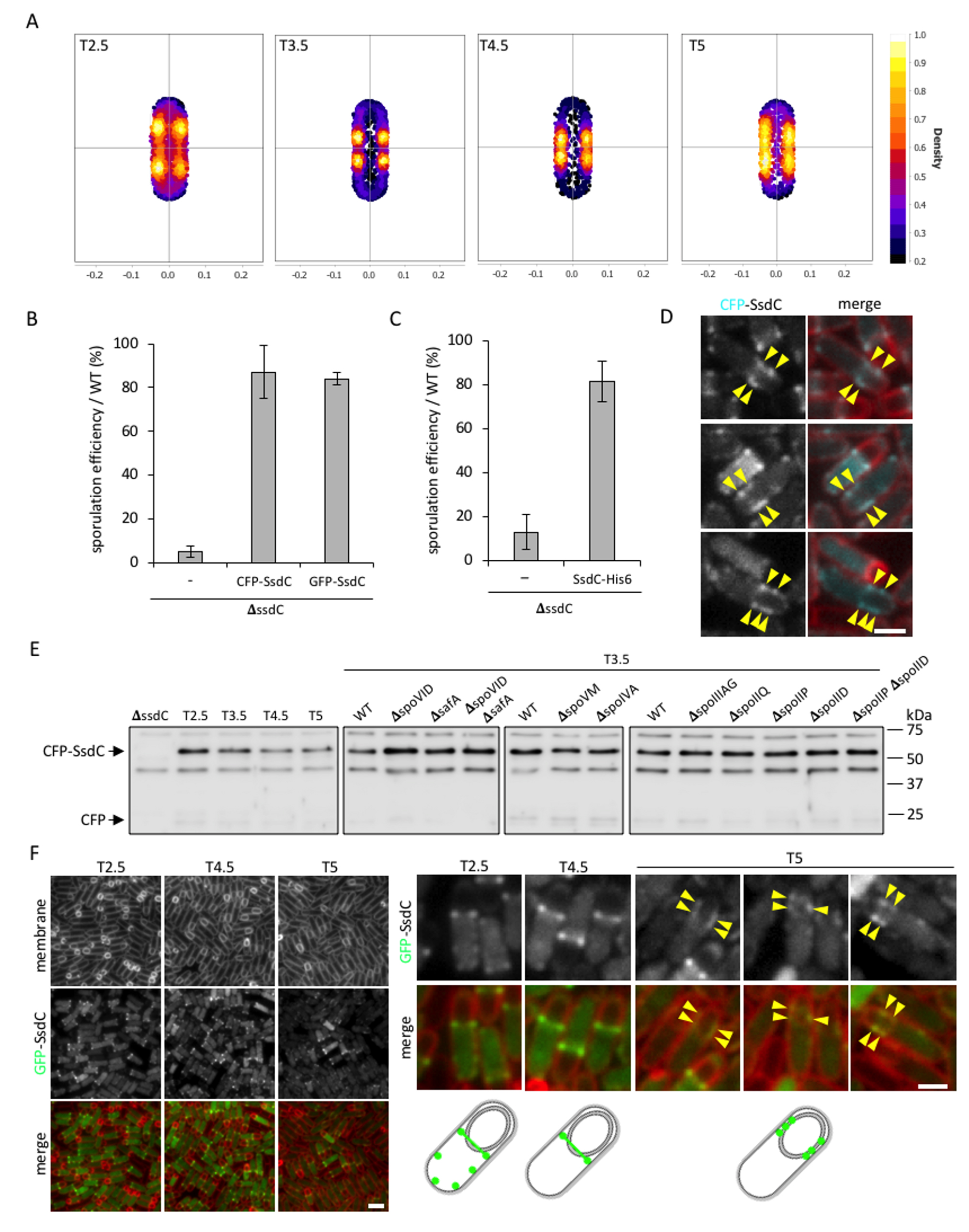

Supplement: S1 Fig — (A) Density maps of CFP-SsdC subcellular fluorescence localization in wild-type (bBK20) cells during a sporulation time-course (n > 400 per time-point). Warmer colours indicate higher density. Although sporulation by resuspension is not perfectly synchronous, many cells are at a similar stage of sporulation at each time-point. Thus, the position of foci relative to the sporangium at a population level at the different time-points provides an overview of how CFP-SsdC foci are changing position at different stages of development. Although this does not take the location of the forespore into account, since it resides at either pole of the sporangium, we can estimate its position within the sporangium relative to the CFP-SsdC foci. (B) Average sporulation efficiency (±STDEV, n = 3) of ΔssdC (bBK3) in the presence and absence of CFP-SsdC (bBK20) and GFP-SsdC (bBK21), relative to wild-type (bDR2413). CFP-SsdC and GFP-SsdC complemented the ΔssdC phenotype and restored sporulation efficiency to 87% and 83% of wild-type, respectively. (C) Average sporulation efficiency (±STDEV of three biological replicates) of ΔssdC (bBK3) in the presence and absence SsdC-His6 (bHC45) relative to wild-type (bDR2413). SsdC-His6 complemented the ΔssdC phenotype and restored sporulation efficiency to 81% of wild-type. (D) Close-up of CFP-SsdC localization in wild-type cells (bBK20) around the spore at T5. CFP signal is false-coloured cyan in merged images. Cell membranes were visualised with TMA-DPH fluorescent membrane dye and are false-coloured red in merged images. Scale bar = 1 μm. (E) Immunoblot analysis of CFP-SsdC in otherwise wild-type (bBK20, WT) or mutant strains. CFP-SsdC was immunodetected using anti-GFP antibodies. Bands present in strains containing CFP-SsdC but not present in ΔssdC (bBK3) correspond to CFP-SsdC and a CFP degradation product. The positions of CFP-SsdC and CFP are indicated by the arrow. From left to right, immunoblots are presented in Figs 2D, 5B, 6C and S7C, re [file pgen.1009246.s001.jpg]

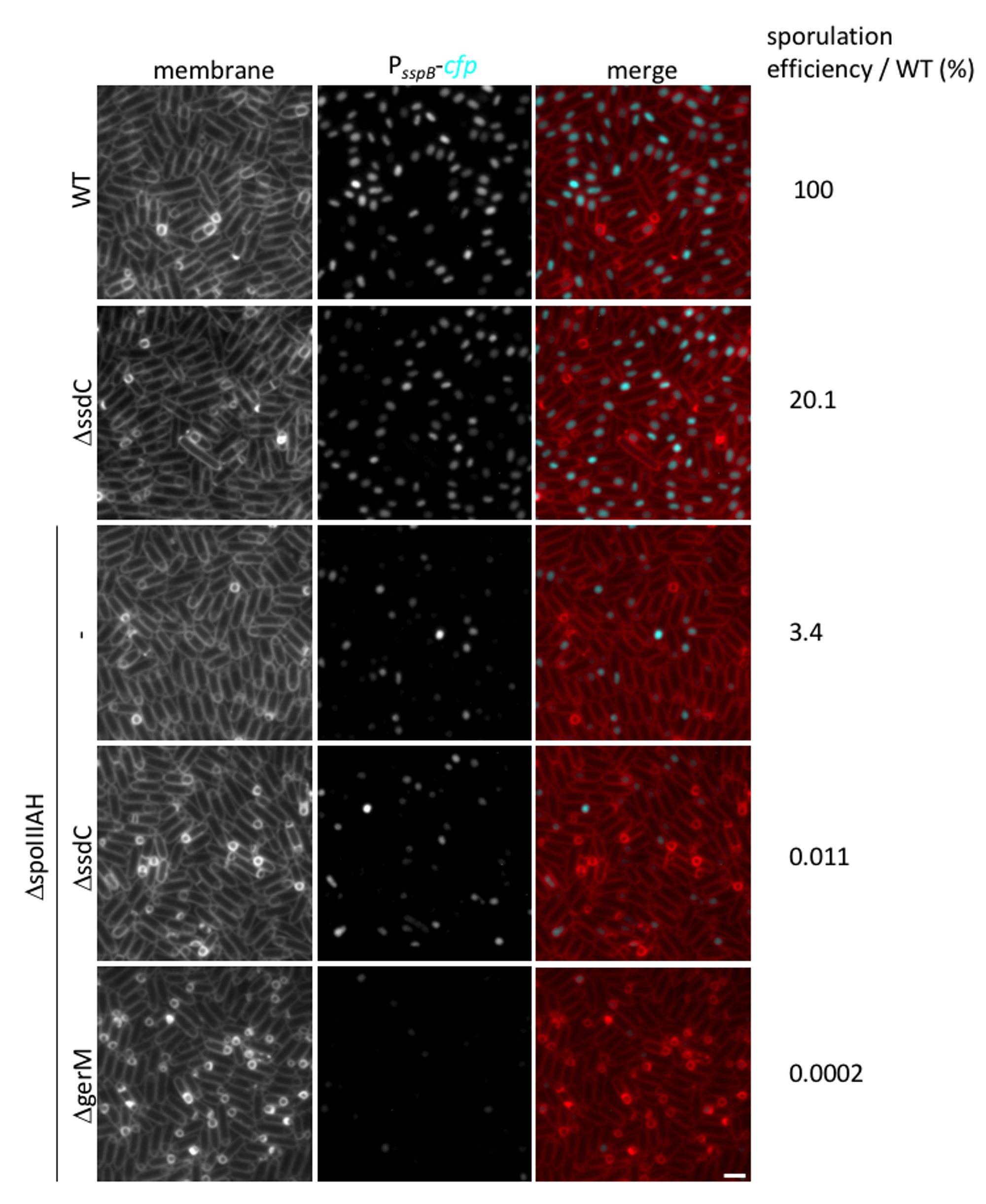

Supplement: S2 Fig — Fluorescence microscopy of σG activity in wild-type (bKH21, WT), ΔssdC (bJL66), ΔspoIIIAH (bKH23), ΔssdC ΔspoIIIAH (bJL178) and ΔgerM ΔspoIIIAH (bJL179). σG activity was visualised using a σG -dependent fluorescent transcriptional reporter (PsspB-cfp, false-coloured cyan in merged images) at T4.5 of sporulation. σG-activity is not reduced in ΔssdC or ΔssdC ΔspoIIIAH mutant strains, relative to the wild-type or ΔspoIIIAH mutant, respectively. As a control, and consistent with previous work [36], the ΔgerM ΔspoIIIAH mutant strain has reduced σG-activity relative to the ΔspoIIIAH mutant. Cell membranes were visualised with TMA-DPH fluorescent membrane dye and are false-coloured red in merged images. Scale bar = 2 μm. Sporulation efficiency of mutant strains relative to WT are shown on the right (n = 2). (JPG) [file pgen.1009246.s002.jpg]

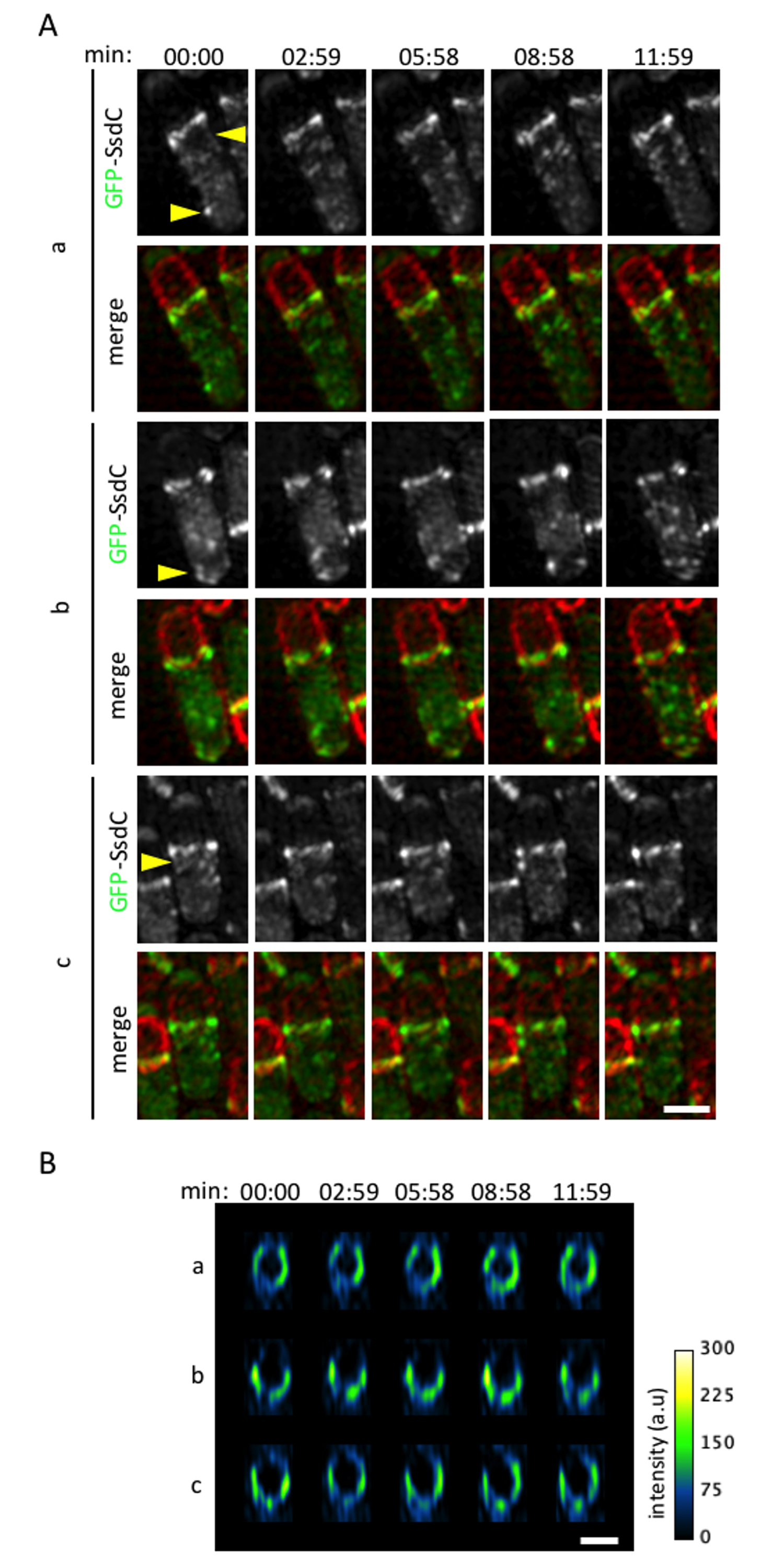

Supplement: S3 Fig — (A) 3D-Structured Illumination Microscopy (3D-SIM) time-lapse of GFP-SsdC localization in wild-type (bBK21). Three examples are shown (a, b & c). GFP signal is false-coloured green in merged images. Yellow arrowheads point to regions of the cell where GFP-SsdC localization changes over the course of the time-lapse. Scale bar = 1 μm. (B) Fluorescence intensity plots of GFP-SsdC rings captured from the same cells above, highlighting changes in GFP-SsdC localization within the ring-like structure over the course of the time-lapse. Scale bar = 1 μm. (JPG) [file pgen.1009246.s003.jpg]

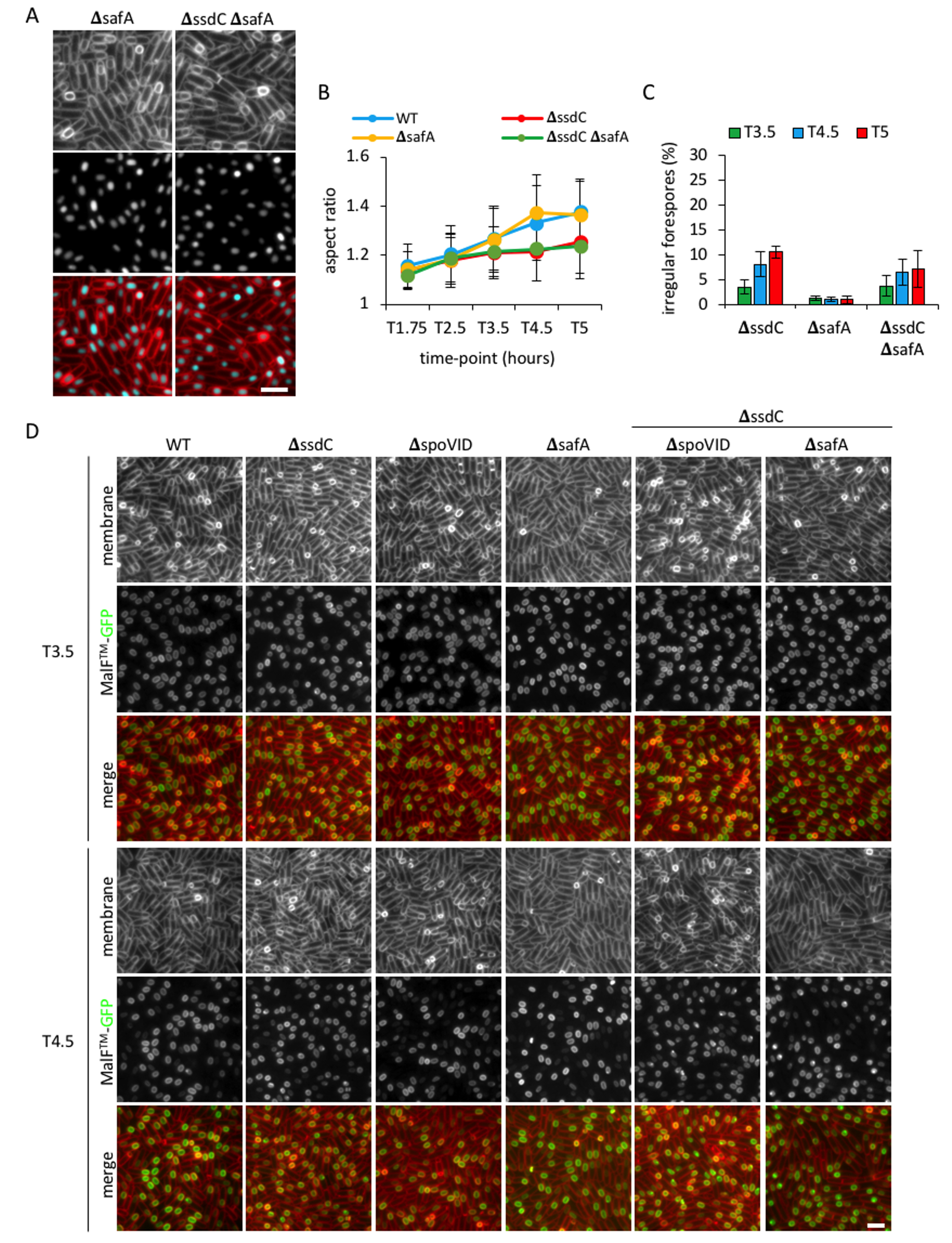

Supplement: S4 Fig — (A) Forespore morphology of ΔsafA (bBK66) and ΔsafA ΔssdC (bBK62) strains at 4.5 h after onset of sporulation (T4.5). Forespore cytoplasm was visualised using a forespore reporter (PspoIIQ-cfp, false-coloured cyan in merged images). Cell membranes were visualised with TMA-DPH fluorescent membrane dye and are false-coloured red in merged images. Scale bar = 2 μm. (B) Average forespore aspect ratio (± STDEVP) of wild-type (WT, bBK17, blue), ΔspoVID (bBK18, red), ΔsafA (bBK66, yellow) and ΔssdC ΔsafA (bBK62, green) mutant strains during a sporulation time-course. n > 500 per time-point, per strain. (C) Histogram showing proportion of sporulating cells (% ± STDEV of three biological replicates) of irregularly-shaped forespores shape in wild-type (WT, bBK17), ΔssdC (bBK18), ΔsafA (bBK66) and ΔssdC ΔsafA (bBK62) mutant strains at 3.5 (T3.5, green), 4.5 (T4.5, blue) and 5 h (T5, red) after the onset of sporulation. Irregular forespores were defined as elongated and distorted in shape. n > 250 per replicate, per time-point, per strain. (D) Forespore morphology of wild-type (WT, bJL78), ΔssdC (bJL79), ΔspoVID (bJL193), ΔsafA (bJL199), ΔssdC ΔspoVID (bJL80) and ΔsafA ΔssdC (bJL81) strains harbouring a MalFTms-GFP inner spore membrane reporter at 3.5 (T3.5) and 4.5 h (T4.5) after onset of sporulation. GFP signal is false-coloured green in merged images. Cell membranes were visualised with TMA-DPH fluorescent membrane dye and are false-coloured red in merged images. Scale bars = 2 μm. (JPG) [file pgen.1009246.s004.jpg]

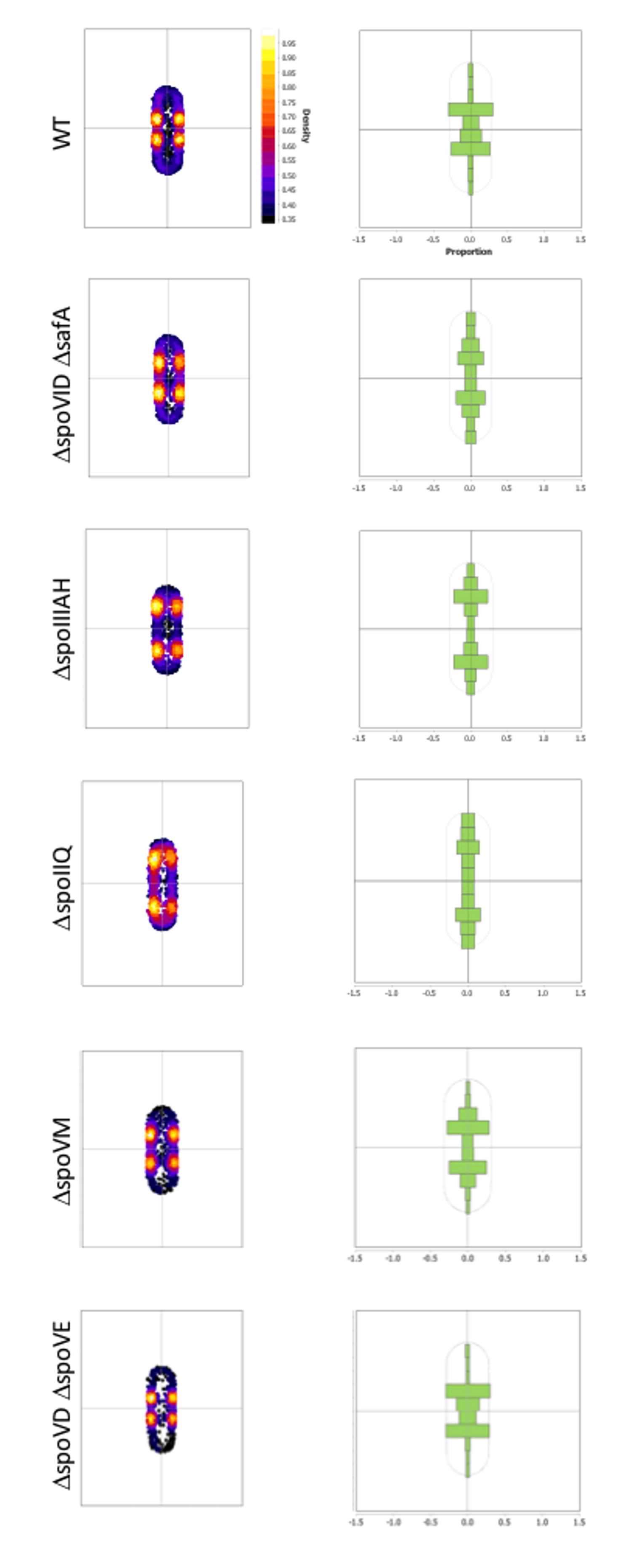

Supplement: S5 Fig — Density maps and histogram plots of CFP-SsdC subcellular fluorescence localization (n > 400 per strain) at 3.5 h after the onset of sporulation (T3.5) in wild-type (bBK20), ΔspoVID ΔsafA (bJL190), ΔspoIIIAH (bBK52), ΔspoIIQ (bJL175), ΔspoVM (bJL33) and ΔspoVD ΔspoVE (bHC99). In the density maps warmer colours indicate higher density. In the histogram plots, the width of each bar indicates the frequency of localization at that specific subcellular location. (JPG) [file pgen.1009246.s005.jpg]

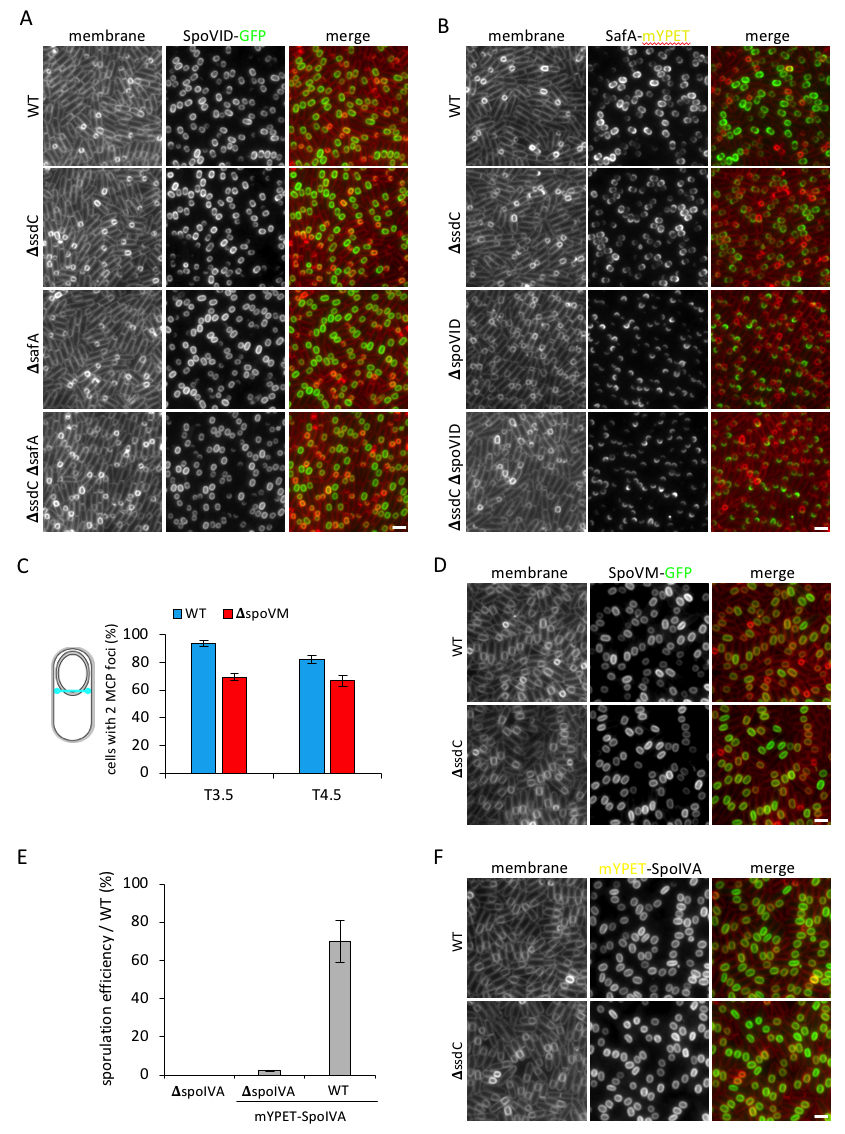

Supplement: S6 Fig — (A) Fluorescence localization of SpoVID-GFP in wild-type (bJL12), ΔssdC (bJL10), ΔsafA (bJL158) and ΔssdC ΔsafA (bJL162) mutant strains at 3.5 h after onset of sporulation (T3.5). (B) Fluorescence localization of SafA-mYPET in wild-type (bJL13, WT), ΔssdC (bJL35), ΔspoVID (bJL159) and ΔssdC ΔspoVID (bJL160) mutant strains at 3.5 h after onset of sporulation (T3.5). mYPET and GFP signals are false-coloured green in merged images. Cell membranes were visualised with TMA-DPH fluorescent membrane dye and are false-coloured red in merged images. Scale bars = 2 μm. (C) Histogram showing proportion of sporulating cells (% ± STDEV, 3 biological replicates) with two MCP CFP-SsdC foci in wild-type (bBK20, WT, blue) and ΔspoVM (bJL33, red) cells at T3.5 and T4.5 of sporulation. n > 400 per replicate, per time-point, per strain. (D) Fluorescence localization of SpoVM-GFP in wild-type (bJL133) and ΔssdC (bJL135) mutant strains at 3.5 h after onset of sporulation (T3.5). (E) Average sporulation efficiency (±STDEV, n = 3) of mYPET-SpoIVA strains in the absence (ΔspoIVA, bJL136) or presence of spoIVA (WT, bJL129), relative to wild-type (bDR2413). The ΔspoIVA strain (bJL59) does not produce heat-resistant spores. mYPET-SpoIVA does not complement the ΔspoIVA phenotype (2.4% sporulation efficiency) and was thus used in a merodiploid background (70% sporulation efficiency). (F) Fluorescence localization of mYPET-SpoIVA in wild-type (bJL129, WT), and ΔssdC (bJL140) mutant strains at 3.5 h after onset of sporulation (T3.5). mYPET and GFP signals are false-coloured green in merged images. Cell membranes were visualised with TMA-DPH fluorescent membrane dye and are false-coloured red in merged images. Scale bars = 2 μm. (JPG) [file pgen.1009246.s006.jpg]

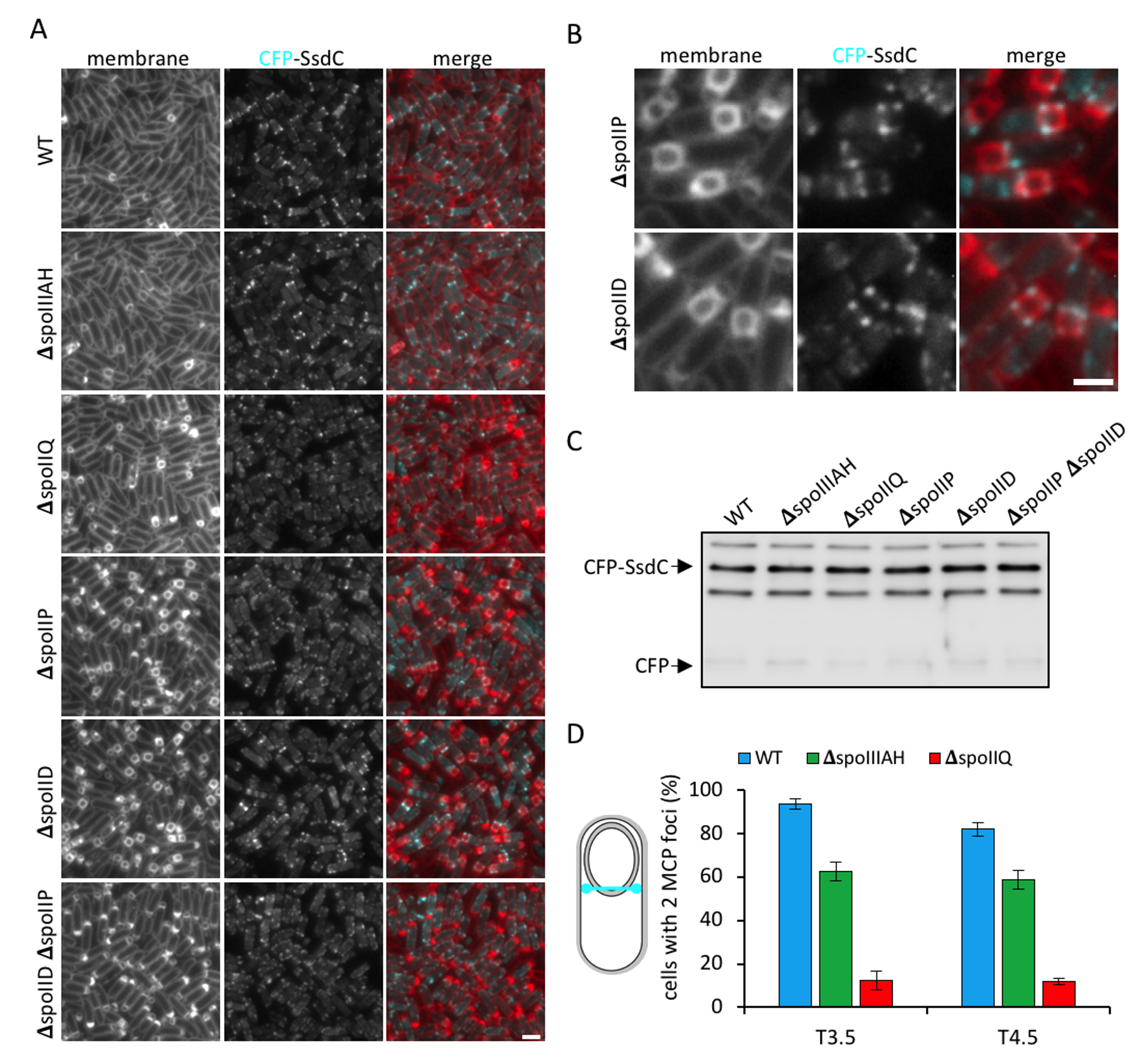

Supplement: S7 Fig — (A) Fluorescence localization of CFP-SsdC in wild-type (bBK20, WT), ΔspoIIIAH (bBK52), ΔspoIIQ (bJL175), ΔspoIIP (bJL176), ΔspoIID (bBK57) and ΔspoIID ΔspoIIP (bJL177) mutant strains at 3.5 h after onset of sporulation (T3.5). CFP signal is false-coloured cyan in merged images. Cell membranes were visualised with TMA-DPH fluorescent membrane dye and are false-coloured red in merged images. Scale bar = 2 μm. (B) Close-up of representative cells in (A), showing CFP-SsdC foci at either side of the membrane bulge in ΔspoIIP (bJL176) and ΔspoIID (bBK57) mutants. Fluorescence signals are false-coloured as in (A). Scale bar = 1 μm. (C) Immunoblot analysis of CFP-SsdC in cell lysates from wild-type (bBK20, WT), ΔspoIIIAH (bBK52), ΔspoIIQ (bJL175), ΔspoIIP (bJL176), ΔspoIID (bBK57) and ΔspoIID ΔspoIIP (bJL177) mutant strains collected at T3.5. CFP-SsdC was immunodetected using anti-GFP antibodies. The positions of CFP-SsdC and CFP are indicated (see also S1E Fig). (D) Histogram showing proportion of cells (% ± STDEV, 3 biological replicates) with two CFP-SsdC mother-cell proximal (MCP) foci in wild-type (bBK20, WT, blue), ΔspoIIIAH (bBK52, green) and ΔspoIIQ (bJL175, red) strains. n > 400 per biological replicate, per time point, per strain. (JPG) [file pgen.1009246.s007.jpg]

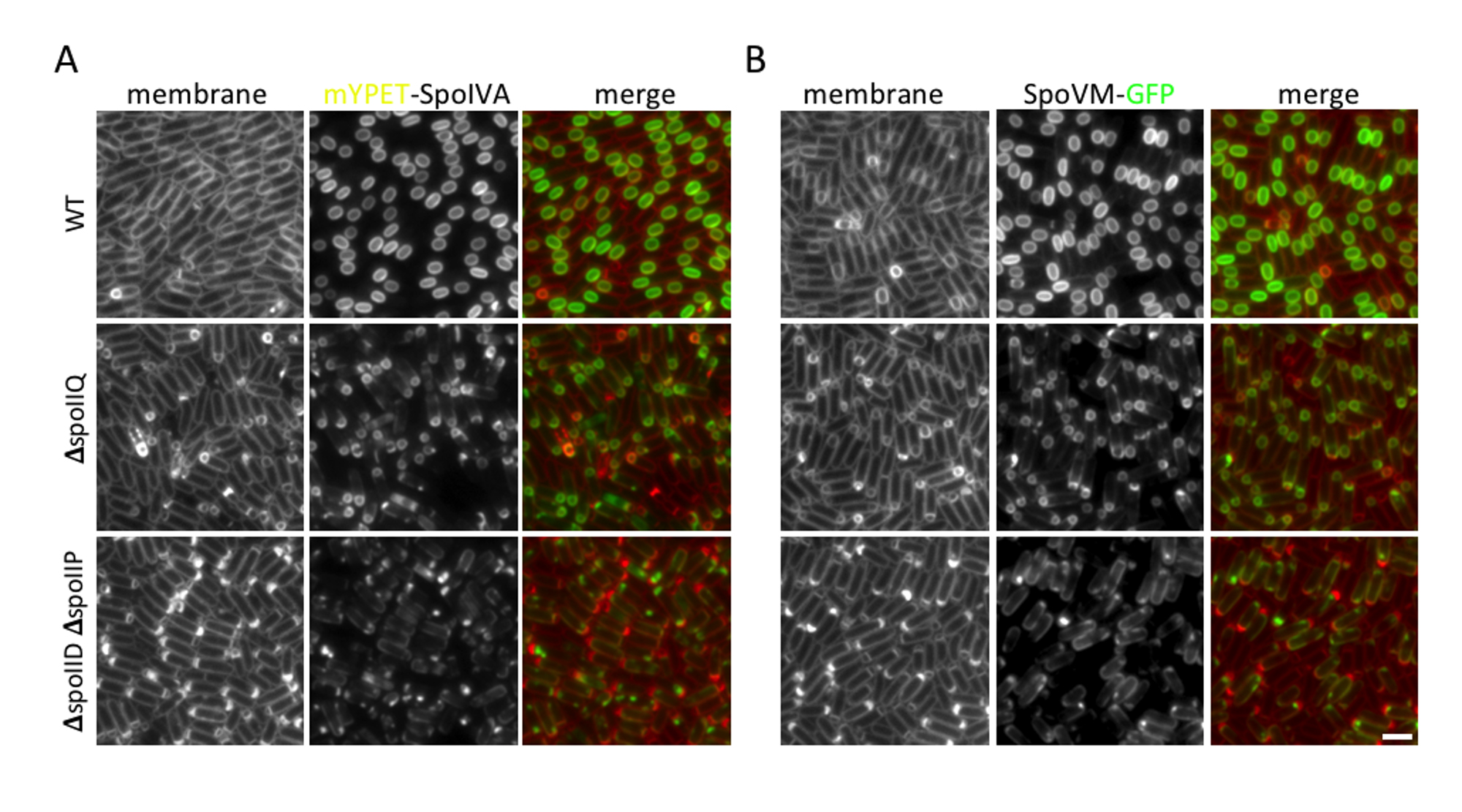

Supplement: S8 Fig — (A) Fluorescence localization of mYPET-SpoIVA in wild-type (bJL129, WT), ΔspoIIQ (bJL185) and ΔspoIID ΔspoIIP (bJL189) mutant strains at 3 h after onset of sporulation (T3). (B) Fluorescence localization of SpoVM-GFP in wild-type (bJL133, WT), ΔspoIIQ (bJL187) and ΔspoIID ΔspoIIP mutant strains (bJL188) at 3 h after onset of sporulation (T3.5). mYPET and GFP signals are false-coloured green in merged images. Cell membranes were visualised with TMA-DPH fluorescent membrane dye and are false-coloured red in merged images. Scale bar = 2 μm. (JPG) [file pgen.1009246.s008.jpg]

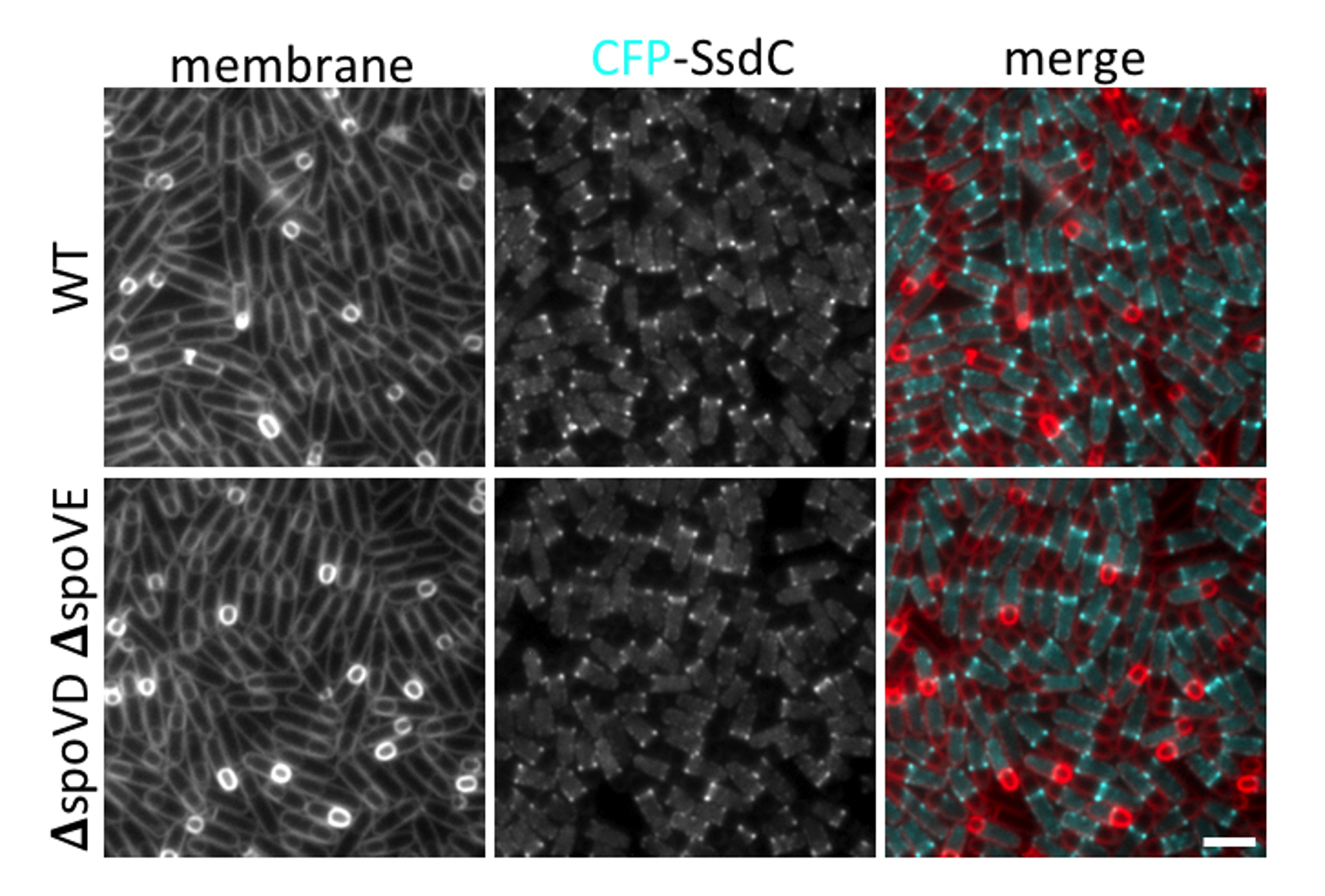

Supplement: S9 Fig — Fluorescence localization of CFP-SsdC in wild-type (bBK20, WT) and ΔspoVD ΔspoVE (bHC99) mutant strain at 3.5 h after onset of sporulation (T3.5). CFP signal is false-coloured cyan in merged images. Cell membranes were visualised with TMA-DPH fluorescent membrane dye and are false-coloured red in merged images. Scale bar = 2 μm. (JPG) [file pgen.1009246.s009.jpg]

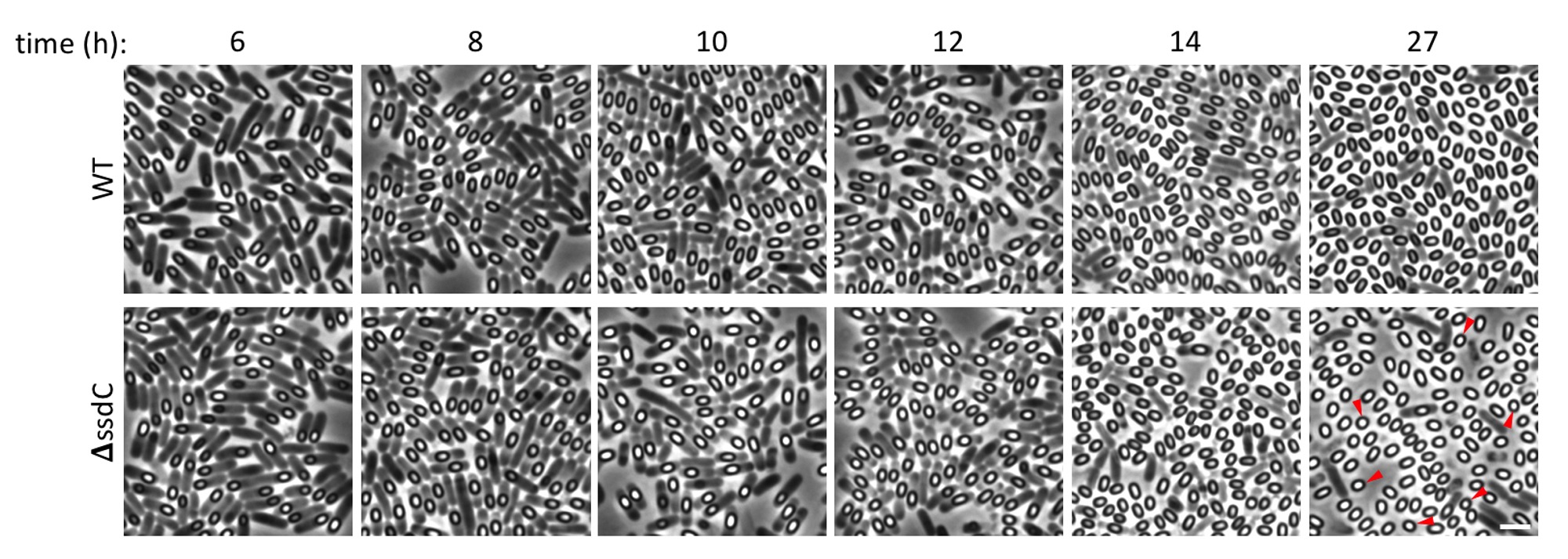

Supplement: S10 Fig — Phase-contrast images of wild-type (bAT87, WT) and ΔssdC (bJL56) strains during a sporulation time-course until spore maturation and release from the mother cell. Arrowheads indicate ΔssdC mutant mature spores that appear rounder than WT. Scale bar = 2 μm. (JPG) [file pgen.1009246.s010.jpg]

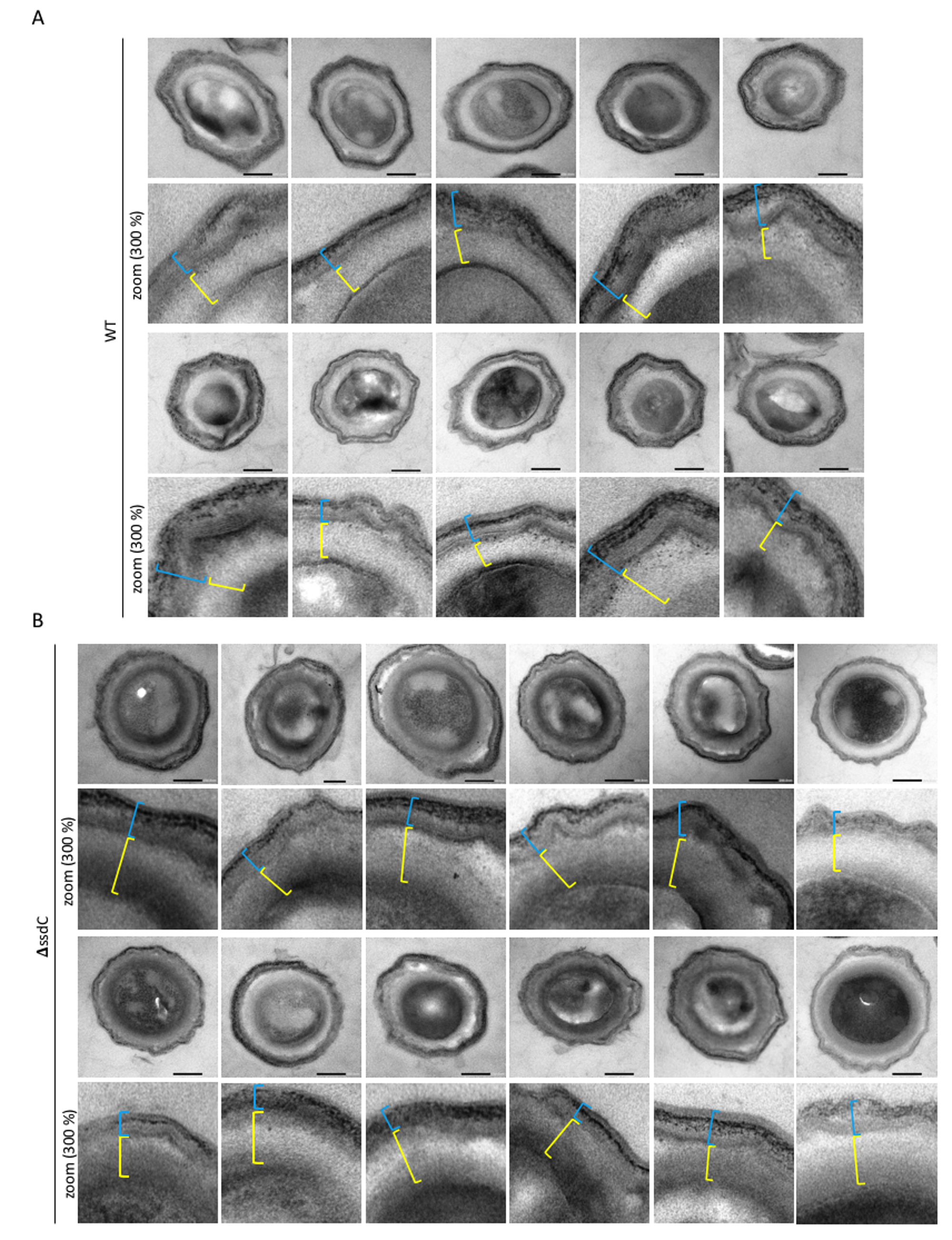

Supplement: S11 Fig — Transmission electron microscopy images of (A) wild-type (bAT87, WT) and (B) ΔssdC (bJL56) mature spores, with respective zoomed-in areas of the spore envelope. Scale bar is 200 nm. The blue bracket indicates the approximate location of the spore coat and crust, whereas the yellow bracket indicates the approximate location of the cortex. (JPG) [file pgen.1009246.s011.jpg]

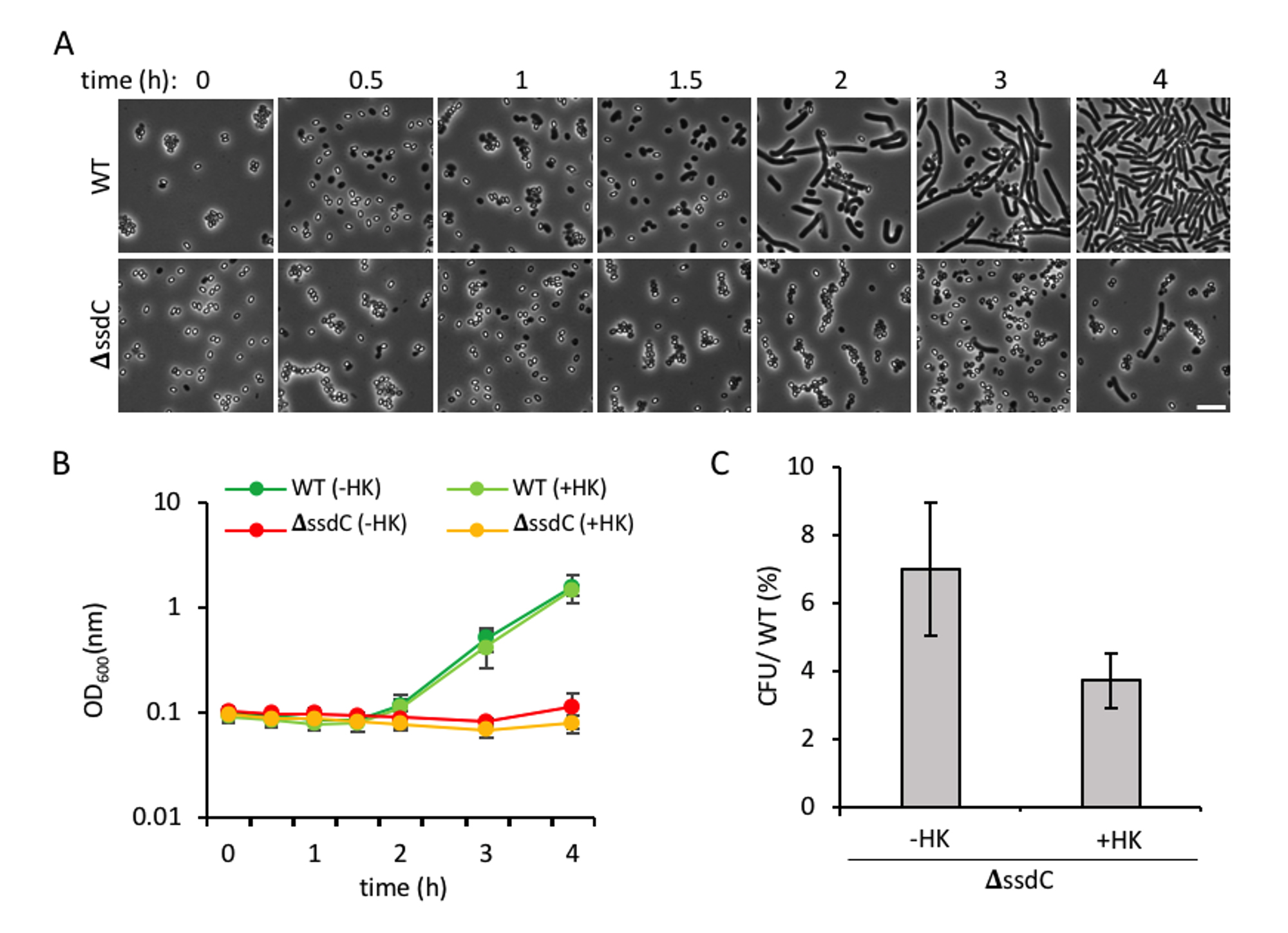

Supplement: S12 Fig — (A) Phase-contrast micrographs of heat-treated (80°C, 20 min) wild-type (bAT87, WT) and ΔssdC (bJL56) spores during a germination and outgrowth time-course in nutrient-rich media (LB). Scale bar = 5 μm. (B) Optical density at 600 nm (OD600, ±STDEV, n = 2) of heat-treated (+HK) wild-type (bAT87, WT, light green) and ΔssdC (bJL56, yellow) spores, and untreated (-HK) wild-type (bAT87, WT, dark green) and ΔssdC (bJL56, red) spores, during a germination and outgrowth time-course in nutrient-rich media (LB). Optical density decreases as spores become phase dark and germination begins. Optical density then increases as outgrowth and vegetative cell division occur. (C) Average germination efficiency (n = 3, ± STDEV) of ΔssdC mutant spores that were plated on LB-agar without heat-treatment (-HK, 7%) and with heat-treatment (+HK) (3.7%) relative to wild-type. (JPG) [file pgen.1009246.s012.jpg]

A

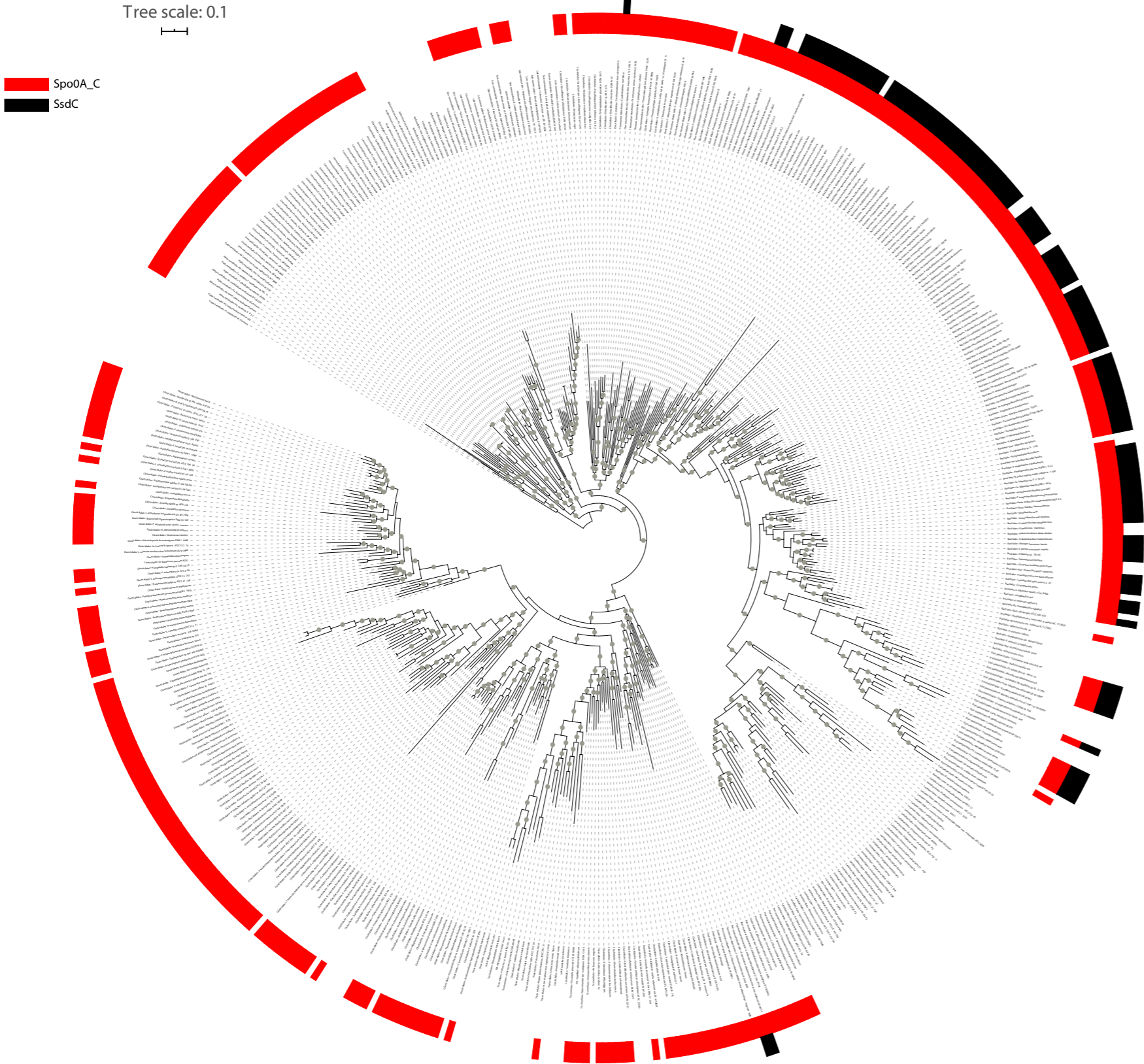

B

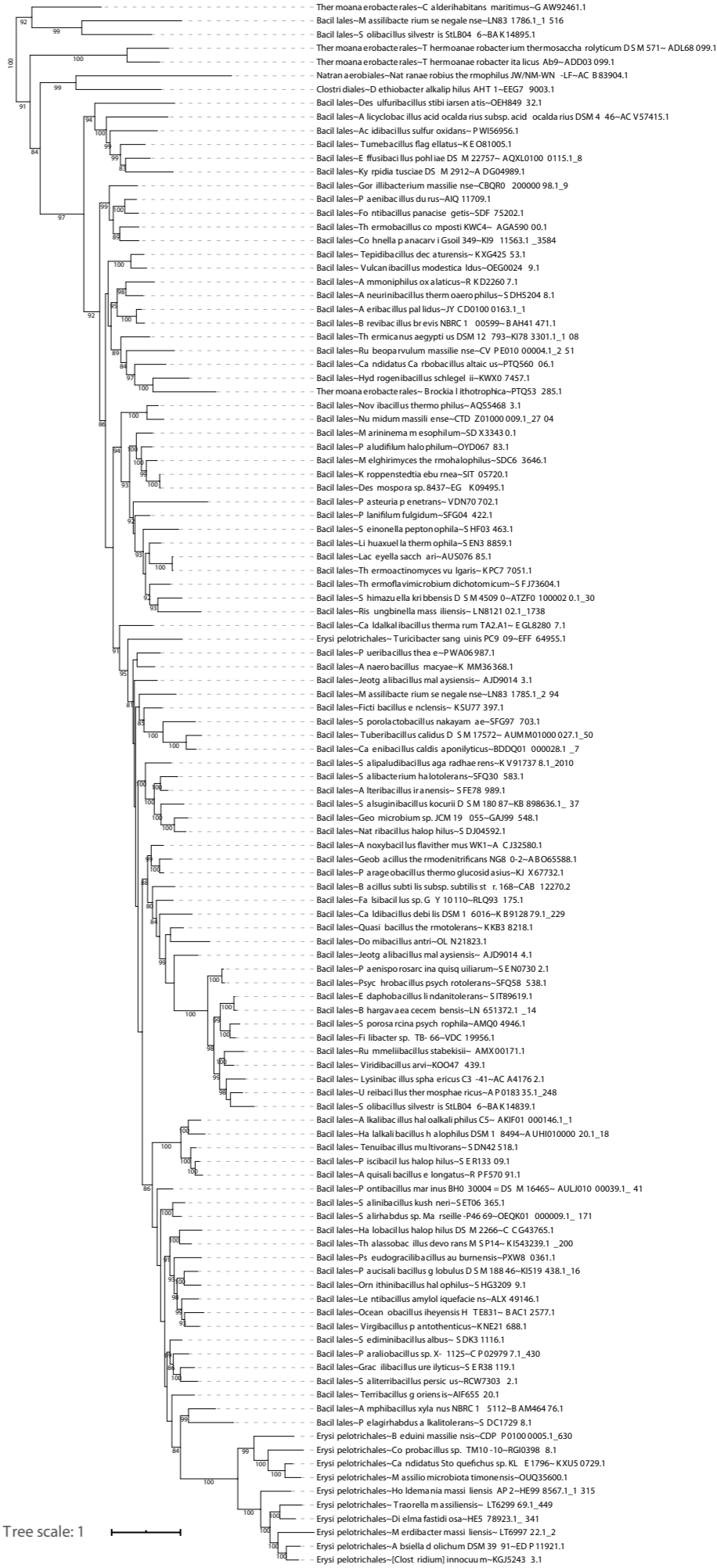

Supplement: S13 Fig — Among the 497 proteomes of Firmicutes, 358 have Spo0A_C homologs and probably sporulate. Within these sporulating taxa, we retrieved SsdC homologs in 114 taxa: 11 Erysipelotrichales, 4 Thermoanaerbacterales, 97 bacillales, 1 Natranaerobiales (Natranaerobius thermophilus JW) and 1 Clostridiales (Dethiobacter alkaliphilus AHT 1). No homologs were identified in Lactobacillales. The gene tree of SsdC homologs follows the species tree with a wide distribution of ssdC in Bacillales. Moreover, the presence of ssdC in N. thermophilus JW and D.alkaliphilus AHT 1, which branch at the base of the Bacilli in both SsdC gene tree and the reference tree of the Firmicutes suggests the presence of SsdC in the ancestor of the bacilli followed by its loss in Lactobacillales. (A) Reference phylogeny of the Firmicutes. Maximum likelihood tree based on concatenation of 29 ribosomal proteins (497 taxa, 3,776 amino acid characters). The tree was inferred with IQ-TREE 1.6.3 using the LG+I+G4 model selected under the BIC criterion. Grey dots correspond to supports higher than 80%. The scale bar corresponds to the average number of substitutions per site. The presence of Spo0A_C and SsdC is indicated in front of each tip in red and black respectively. (B) Phylogenetic tree of SsdC homologs retrieved in the Firmicutes databank. Maximum likelihood tree based on an alignment of 114 taxa and 244 amino acid positions. The tree was inferred with IQ-TREE using the LG+F+I+G4 model selected under BIC criterion. Node supports higher than 80% are displayed. The scale bar corresponds to the average number of substitutions per site. (PDF) [file pgen.1009246.s013.pdf]

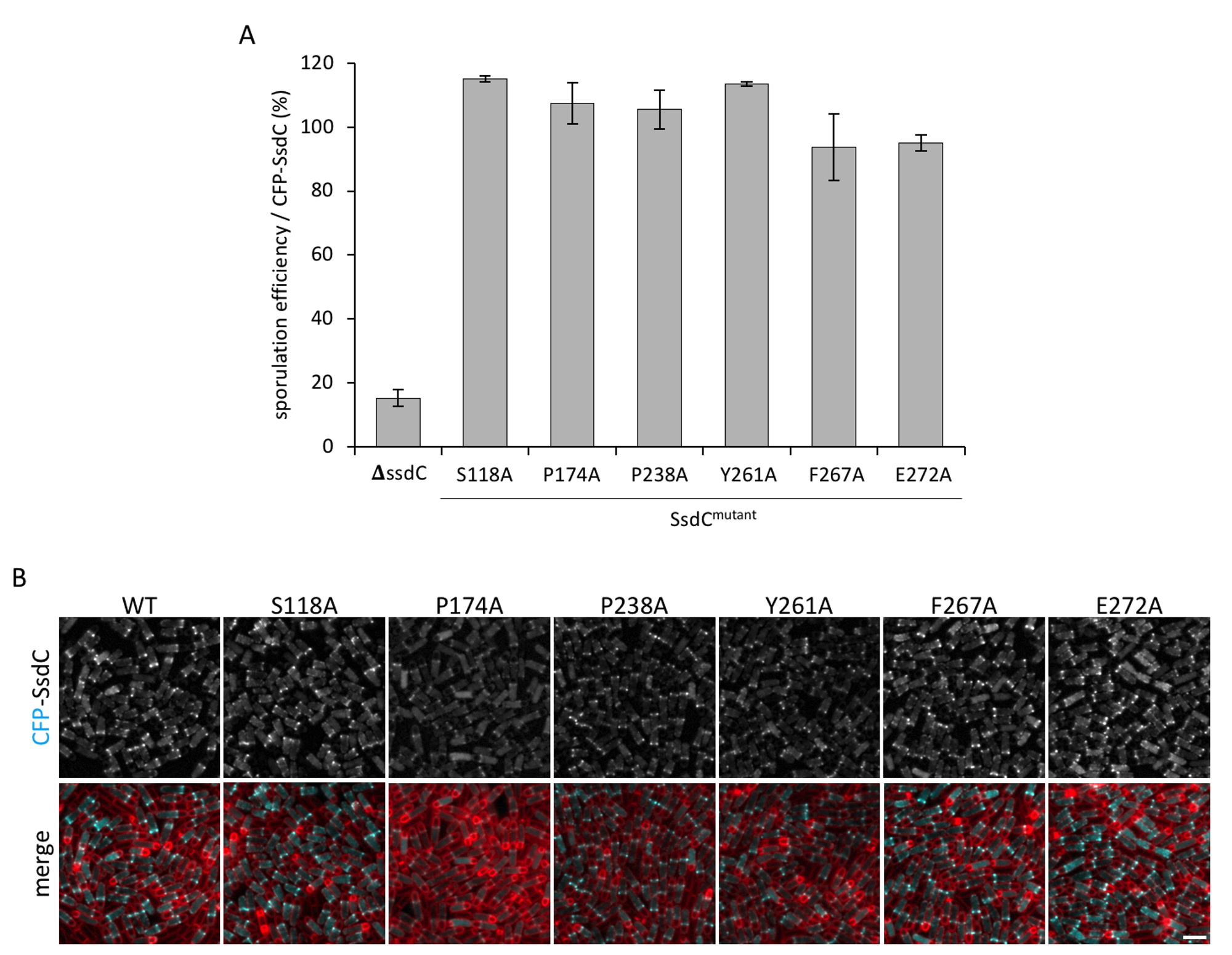

Supplement: S14 Fig — (A) Average sporulation efficiency (±STDEV, n = 2) of ΔssdC (bBK3) and CFP-SsdC mutants S118A (bHC144), P174A (bHC25), P238A (bHC27), Y261A (bHC29), F267A (bHC31) and E272A (bHC33), relative to CFP-SsdC wild-type (bBK20). Mutation of either of these residues did not negatively affect sporulation efficiency. (B) Fluorescence localization of CFP-SsdC wild-type (bBK20, WT), S118A (bHC144), P174A (bHC25), P238A (bHC27), Y261A (bHC29), F267A (bHC31) and E272A (bHC33) strains at 3.5 h after onset of sporulation (T3.5). CFP signal is false-coloured cyan in merged images. Cell membranes were visualised with TMA-DPH fluorescent membrane dye and are false-coloured red in merged images. Scale bar = 2 μm. (JPG) [file pgen.1009246.s014.jpg]
