# Supplementary material for: A dynamic, ring-forming MucB / RseB-like protein influences spore shape in Bacillus subtilis
Source: PLoS Genet. 2020 Dec 14;16(12):e1009246. doi: 10.1371/journal.pgen.1009246 (PMC7769602; doi:10.1371/journal.pgen.1009246)
Supplement: S1 Table — a Fold-difference in the number of transposon insertions: e.g. the ΔssdC mutant had 333.3-fold less transposon insertions than the WT. b Sporulation efficiency, relative to WT, of ΔssdC double mutants with the listed genes: e.g. the ΔssdC ΔcotE double mutant had a sporulation efficiency of 0.16%. The data is average of two biological replicates. c Fold-change in sporulation efficiency of the ΔssdC double mutants with the listed genes, relative to the ΔssdC single mutant: the ΔssdC ΔcotE mutant produced 20.1-fold less spores that the ΔssdC single mutant. (PDF) [file pgen.1009246.s015.pdf]

S1 Table. Independently validated sporulation genes identified by Tn-seq.

Gene	*p*-value	Tn. fold change Δ*ssdC* /WT[a]	Possible product	Spo. in Δ*ssdC* (% ± STDEV)[b]	Spo. fold change / Δ*ssdC*[c]
ssdC	-	-	RseB-like protein required for spore shape	3.21 ± 1.71	-
cotE	3.20×10^{-4}	333.3	Outer spore coat morphogenetic protein	0.16 ± 1.09	20.1
cotZ	4.20×10^{-4}	71.4	Spore crust protein, necessary for the assembly of the spore crust	2.61 ± 1.31	1.23
ctpB	$< 10^{-7}$	37.0	Processing serine protease, cleaves SpoIVFA.	0.90 ± 0.29	3.58
dltB	$< 10^{-7}$	2.3	D-alanine transfer from DltC to undecaprenol-phosphate	1.27 ± 0.63	2.52
dltD	1.66×10^{-4}	4.0	D-alanine transfer from undecaprenol-phosphate to the poly(glycerophosphate) chain	1.80 ± 0.72	1.79
mcsA	1.20×10^{-5}	6.9	Modulator of CtsR dependent repression	1.18 ± 0.50	2.73
rpsT	4.13×10^{-3}	333.3	Ribosomal protein	2.99 ± 1.80	1.07
safA	$< 10^{-7}$	25.6	Spore coat morphogenetic protein and major organizer of the inner spore coat	0.07 ± 0.02	48.3
skfA	$< 10^{-7}$	2.6	Spore killing factor	2.41 ± 0.01	1.33
spoVID	1.50×10^{-4}	250	Spore coat morphogenetic protein, promotes encasement of the spore	0.09 ± 0.03	36.2
yacL	1.40×10^{-4}	3.2	Unknown	0.46 ± 0.20	7.04
yceE	1.90×10^{-2}	4.2	Unknown	1.76 ± 1.08	1.82
yjgD	2.32×10^{-2}	6.8	Unknown	2.55 ± 0.55	1.26
ylmD	2.00×10^{-5}	5.6	Peptidoglycan-editing factor	3.43 ± 0.90	0.94
ymzB	7.55×10^{-3}	2.6	Unknown	2.17 ± 1.41	1.48
yocH	3.00×10^{-6}	3.3	Peptidoglycan hydrolase	1.82 ± 0.23	1.77
yodJ	1.31×10^{-2}	2.3	LD-carboxypeptidase	0.42 ± 0.37	7.59
ypbE	2.33×10^{-3}	2.3	Peptidoglycan-binding protein	3.22 ± 0.81	1.00
yqfT	4.02×10^{-2}	125	Unknown	0.94 ± 0.50	3.43
yqhG	6.00×10^{-5}	333	Unknown	1.19 ± 0.76	2.69
yqhH	1.00×10^{-5}	6.0	Similar to sucrose non-fermenting 2 helicase	1.21 ± 1.07	2.65
yqhQ	4.40×10^{-4}	3.4	Unknown	2.14 ± 0.23	1.50
yrrI	1.21×10^{-3}	4.0	Similar to coordinator of zonal elongation	0.54 ± 0.35	5.92
yrrL	2.00×10^{-6}	1.9	Potential terminase for peptidoglycan polymerization	2.77 ± 0.55	1.16
yteV	8.81×10^{-4}	250	Unknown	2.33 ± 0.76	1.38
yxiS	2.79×10^{-2}	2.1	Unknown	2.51 ± 1.75	1.28

Legend:

a Fold-difference in the number of transposon insertions: e.g. the Δ*ssdC* mutant had 333.3-fold less transposon insertions than the WT.

b Sporulation efficiency, relative to WT, of Δ*ssdC* double mutants with the listed genes: e.g. the Δ*ssdC* Δ*cotE* double mutant had a sporulation efficiency of 0.16 %. The data is average of two biological replicates.

c Fold-change in sporulation efficiency of the Δ*ssdC* double mutants with the listed genes, relative to the Δ*ssdC* single mutant: the Δ*ssdC* Δ*cotE* mutant produced 20.1-fold less spores that the Δ*ssdC* single mutant.
