# Supplementary material for: A dynamic, ring-forming MucB / RseB-like protein influences spore shape in Bacillus subtilis
Source: PLoS Genet. 2020 Dec 14;16(12):e1009246. doi: 10.1371/journal.pgen.1009246 (PMC7769602; doi:10.1371/journal.pgen.1009246)
Supplement: S2 Table — a Fold-difference in the number of transposon insertions: e.g. the ΔssdC mutant had 333.3-fold less transposon insertions than the WT. (PDF) [file pgen.1009246.s016.pdf]

S2 Table. Spore coat and spore crust genes identified by Tn-seq.

Gene	*p*-value	Tn. fold change ∆ssdC /WT[a]	Possible product
cotC	1.95×10^{-2}	1.6	Spore coat protein (outer)
cotE	3.20×10^{-4}	333.3	Outer spore coat morphogenetic protein
cotG	7.91×10^{-3}	1.2	Outer spore coat protein
cotH	$< 10^{-7}$	6.5	Protein kinase, required for the assembly of several outer coat proteins
cotJA	4.35×10^{-2}	3.0	Unknown, polypeptide composition of the spore coat
cotM	1.08×10^{-2}	2.6	Spore coat protein (outer)
cotO	1.43×10^{-2}	1.3	Spore coat morphogenetic protein
cotR	4.26×10^{-2}	1.1	Outer spore coat lipolytic enzyme
cotS	2.27×10^{-2}	1.5	Outer spore coat protein
cotSA	1.78×10^{-2}	1.4	Spore coat protein
cotY	2.11×10^{-3}	500.0	Main structural component of the spore crust
cotZ	4.20×10^{-4}	71.4	Spore crust anchor protein
safA	$< 10^{-7}$	25.6	Spore coat morphogenetic protein and major organizer of the inner spore coat
spoVID	1.50×10^{-4}	250.0	Spore coat morphogenetic protein, promotes encasement of the spore

Legend:

a Fold-difference in the number of transposon insertions: e.g. the ∆*ssdC* mutant had 333.3-fold less transposon insertions than the WT.
