# Supplementary material for: A dynamic, ring-forming MucB / RseB-like protein influences spore shape in Bacillus subtilis
Source: PLoS Genet. 2020 Dec 14;16(12):e1009246. doi: 10.1371/journal.pgen.1009246 (PMC7769602; doi:10.1371/journal.pgen.1009246)
Supplement: S3 Table — All Bacillus subtilis strains used in this study. (PDF) [file pgen.1009246.s017.pdf]

S3 TABLE: *Bacillus subtilis* strains used in this study

Strains	Genotype	Source
**168**	*Prototrophic wild-type*	(Zeigler et al., 2008)
**bAT87**	*amyE::PspoIIQ-cfp(Bs) (cat)*	This work
**bBK3**	*ssdC::erm*	This work
**bBK15**	*ywjI::PspoIIQ-cfp (cat)*	This work
**bBK17**	*ywjI::PspoIIQ-cfp (cat), amyE::PspoIID-yfp (spec)*	This work
**bBK18**	*ssdC::erm, ywjI::PspoIIQ-cfp (cat), amyE::PspoIID-yfp (spec)*	This work
**bBK20**	*ycgO::PssdC-opt$_{RBS}$-cfp-ssdC (spec), ssdC::erm*	This work
**bBK21**	*ycgO::PssdC-opt$_{RBS}$-SFgfp-ssdC (spec), ssdC::erm*	This work
**bBK28**	*spoVID::kan*	This work
**bBK33**	*safA::kan*	This work
**bBK39**	*ylmD::kan, ssdC::erm*	This work
**bBK41**	*yqhH::kan, ssdC::erm*	This work
**bBK42**	*yqhG::kan, ssdC::erm*	This work
**bBK43**	*spoVID::kan, ssdC::erm*	This work
**bBK44**	*rpsT::kan, ssdC::erm*	This work
**bBK45**	*yteV::kan, ssdC::erm*	This work
**bBK46**	*skfA::kan, ssdC::erm*	This work
**bBK47**	*ctpB::kan, ssdC::erm*	This work
**bBK48**	*safA::kan, ssdC::erm*	This work
**bBK49**	*cotZ::kan, ssdC::erm*	This work
**bBK50**	*yqfT::kan, ssdC::erm*	This work
**bBK51**	*mcsA:kan. ssdC::erm*	This work
**bBK52**	*ycgO::PssdC-opt$_{RBS}$-cfp-ssdC (spec), ssdC::erm, spoIIIAH::kan*	This work
**bBK54**	*ycgO::PssdC-opt$_{RBS}$-cfp-ssdC (spec), ssdC::erm, spoVID::kan*	This work
**bBK56**	*ycgO::PssdC-opt$_{RBS}$-cfp-ssdC (spec), ssdC::erm, safA::kan*	This work
**bBK57**	*ycgO::PssdC-opt$_{RBS}$-cfp-ssdC (spec), ssdC::erm, spoIID::kan*	This work
**bBK60**	*ssdC::erm, spoVID::kan, ywjI::PspoIIQ-cfp (cat), amyE::PspoIID-yfp (spec)*	This work
**bBK62**	*ssdC::erm, safA::kan, ywjI::PspoIIQ-cfp (cat), amyE::PspoIID-yfp (spec)*	This work
**bBK64**	*spoVID::kan, ywjI::PspoIIQ-cfp (cat), amyE::PspoIID-yfp (spec)*	This work
**bBK66**	*safA::kan, ywjI::PspoIIQ-cfp (cat), amyE::PspoIID-yfp (spec)*	This work
**bCR1565**	*ssdC::lox72*	This work
**bHC25**	*ycgO::PssdC-opt$_{RBS}$-cfp-ssdC(P174A) (spec), ssdC::erm*	This work
**bHC27**	*ycgO::PssdC-opt$_{RBS}$-cfp-ssdC(P238A) (spec), ssdC::erm*	This work
**bHC29**	*ycgO::PssdC-opt$_{RBS}$-cfp-ssdC(Y261A) (spec), ssdC::erm*	This work
**bHC31**	*ycgO::PssdC-opt$_{RBS}$-cfp-ssdC(F267A) (spec), ssdC::erm*	This work
**bHC33**	*ycgO::PssdC-opt$_{RBS}$-cfp-ssdC(E272A) (spec), ssdC::erm*	This work
**bHC45**	*ycgO::PssdC-ssdC-His6 (spec), ssdC::erm*	This work
**bHC70**	*spoIIQ::cat, ycgO::PssdC-RBS-ssdC-His6 (spec), ssdC::erm*	This work
**bHC78**	*cotE::kan, ssdC::erm*	This work
**bHC99**	*ssdC::erm, ycgO::PssdC-optRBS-CFP(8aa)-ssdC (spec), spoVD::markerless, spoVE::kan*	This work
**bHC144**	*ycgO::PssdC-opt$_{RBS}$-cfp-ssdC (S118A) (spec), ssdC::erm*	This work
**bJL3**	*ywjI::PspoIIQ-cfp (cat), spoVE::kan, spoVD::spec*	This work
**bJL10**	*ssdC::erm, spoVIDΩspoVID-gfp (spec)*	This work
**bJL12**	*spoVIDΩspoVID-gfp (spec)*	This work
**bJL13**	*ycgO::PsafA-safA-mYPET (spec)*	This work
**bJL33**	*spoVM::erm, ycgO::PssdC-opt$_{RBS}$-cfp-ssdC (spec), ssdC::lox72*	This work
**bJL34**	*spoIVA::cat, ycgO::PssdC-opt$_{RBS}$-cfp-ssdC (spec), ssdC::erm*	This work
**bJL35**	*ycgO::PsafA-safA-mYPET (spec), ssdC::erm*	This work
**bJL39**	*spoVM::erm, ywjI::PspoIIQ-cfp (cat)*	This work
**bJL40**	*spoIVA::cat, ycgO::PssdC-optRBS-sGFP(15aa)-ssdC (spec)*	This work

**bJL43**	*lacA::PspoIIQ-cfp(erm), spoIVA::cat*	This work
**bJL44**	*spoVE::kan, spoVD::spec, ycgO::PssdC-opt*$_{RBS}$*-cfp-ssdC (spec), ssdC::erm*	This work
**bJL56**	*amyE::PspoIIQ-cfp(Bs) (cat), ssdC::erm*	This work
**bJL59**	*spoIVA::cat*	This work
**bJL66**	*yycR::PsspB-rbsopt-cfp (phleo), ssdC::erm*	This work
**bJL78**	*amyE::PspoIIQ-malF(2TM)-gfpΩcat*	This work
**bJL79**	*amyE::PspoIIQ-malF(2TM)-gfpΩcat, ssdC::erm*	This work
**bJL80**	*amyE::PspoIIQ-malF(2TM)-gfpΩcat, ssdC::erm, spoVID::kan*	This work
**bJL81**	*amyE::PspoIIQ-malF(2TM)-gfpΩcat, ssdC::erm, safA::kan*	This work
**bJL82**	*ssdC::erm, ywjl::PspoIIQ-cfp (cat), spoVE::kan, spoVD::spec*	This work
**bJL107**	*yocH::kan, ssdC::erm*	This work
**bJL108**	*yodJ::kan, ssdC::erm*	This work
**bJL109**	*yceE::kan, ssdC::erm*	This work
**bJL110**	*yjgD::kan, ssdC::erm*	This work
**bJL111**	*dltD::kan, ssdC::erm*	This work
**bJL112**	*ypbE::kan, ssdC::erm*	This work
**bJL114**	*yqhQ::kan, ssdC::erm*	This work
**bJL115**	*yrrL::kan, ssdC::erm*	This work
**bJL117**	*yxiS::kan, ssdC::erm*	This work
**bJL118**	*ymzB::kan, ssdC::erm*	This work
**bJL119**	*yrrI::kan, ssdC::erm*	This work
**bJL120**	*yacL::kan, ssdC::erm*	This work
**bJL121**	*dltB::kan, ssdC::erm*	This work
**bJL129**	*ycgO::PspoIVA-mYPET-spoIVA (cat)*	This work
**bJL133**	*spoVMΩspoVM-gfp (spec)*	This work
**bJL135**	*spoVMΩspoVM-gfp (spec), ssdC::erm*	This work
**bJL136**	*spoIVA::neo, ycgO::PspoIVA-mYPET-spoIVA (cat)*	This work
**bJL140**	*ssdC::erm, ycgO::PspoIVA-mYPET-spoIVA (cat)*	This work
**bJL158**	*spoVIDΩspoVID-gfp (spec), safA::kan*	This work
**bJL159**	*ycgO::PsafA-safA-mYPET (spec), spoVID::kan*	This work
**bJL160**	*ycgO::PsafA-safA-mYPET (spec), ssdC::erm, spoVID::kan*	This work
**bJL162**	*ssdC::erm, spoVIDΩspoVID-gfp (spec), safA::kan*	This work
**bJL175**	*ycgO::PssdC-opt*$_{RBS}$*-cfp-ssdC (spec), ssdC::erm, spoIIQ::cat*	This work
**bJL176**	*ycgO::PssdC-opt*$_{RBS}$*-cfp-ssdC (spec), ssdC::erm, spoIIP::tet*	This work
**bJL177**	*ycgO::PssdC-opt*$_{RBS}$*-cfp-ssdC (spec), ssdC::erm, spoIID::kan, spoIIP::tet*	This work
**bJL178**	*yycR::PsspB-rbsopt-cfp (phleo), spoIIIAH (spec), ssdC::erm*	This work
**bJL179**	*yycR::PsspB-rbsopt-cfp (phleo), spoIIIAH (spec), gerM::erm*	This work
**bJL185**	*spoIIQ::erm, ycgO::PspoIVA-mYPET-spoIVA (cat)*	This work
**bJL187**	*spoIIQ::erm, spoVMΩspoVM-gfp (spec)*	This work
**bJL188**	*spoVMΩspoVM-gfp (spec), spoIIP::tet, spoIID::kan*	This work
**bJL189**	*ycgO::PspoIVA-mYPET-spoIVA (cat), spoIIP::tet, spoIID::kan*	This work
**bJL190**	*safA::tet, ycgO::PssdC-opt*$_{RBS}$*-cfp-ssdC (spec), ssdC::erm, spoVID::kan*	This work
**bJL193**	*amyE::PspoIIQ-malF(2TM)-gfpΩcat, spoVID::kan*	This work
**bJL196**	*safA::tet, spoVID::kan, ywjI::PspoIIQ-cfp (cat), amyE::PspoIID-yfp(JF)(spec)*	This work
**bJL199**	*amyE::PspoIIQ-malF(2TM)-gfpΩcat, safA::kan*	This work
**bKH21**	*yycR::PsspB-rbsopt-cfp (phleo)*	This work
**bKH23**	*yycR::PsspB-rbsopt-cfp (phleo), spoIIIAH (spec)*	This work
