# Supplementary material for: A dynamic, ring-forming MucB / RseB-like protein influences spore shape in Bacillus subtilis
Source: PLoS Genet. 2020 Dec 14;16(12):e1009246. doi: 10.1371/journal.pgen.1009246 (PMC7769602; doi:10.1371/journal.pgen.1009246)
Supplement: S4 Table — All plasmids used in this study. (PDF) [file pgen.1009246.s018.pdf]

S4 TABLE: Plasmids used in this study

Plasmids	Description	Source
**pBK2**	*ycgO::cfp (spec)*	This work
**pBK7**	*ycgO::SFgfp (spec)*	This work
**pBK9**	*ycgO: PssdC-opt*$_{RBS}$*-SFgfp (spec)*	This work
**pBK10**	*ycgO::PssdC-opt*$_{RBS}$*-SFgfp-ssdC (spec)*	This work
**pBK16**	*ycgO::PssdC-opt*$_{RBS}$*-cfp (spec)*	This work
**pBK17**	*ycgO::PssdC-opt*$_{RBS}$*-cfp-ssdC (spec)*	This work
**pHC7**	*ycgO::PssdC-opt*$_{RBS}$*-cfp-ssdC (P174A) (spec)*	This work
**pHC8**	*ycgO::PssdC-opt*$_{RBS}$*-cfp-ssdC (P238A) (spec)*	This work
**pHC9**	*ycgO::PssdC-opt*$_{RBS}$*-cfp-ssdC (Y261A) (spec)*	This work
**pHC10**	*ycgO::PssdC-opt*$_{RBS}$*-cfp-ssdC (F267A) (spec)*	This work
**pHC11**	*ycgO::PssdC-opt*$_{RBS}$*-cfp-ssdC (E272A) (spec)*	This work
**pHC22**	*ycgO::PssdC-ssdC-His6 (spec)*	This work
**pHC51**	*ycgO::PssdC-opt*$_{RBS}$*-cfp-ssdC (S118A) (spec)*	This work
**pJL1**	*ycgO::PsafA-safA-mYPET (spec)*	This work
**pJL6**	*ycgO::PspoIVA-mYPET-spoIVA (cat)*	This work
