# Supplementary material for: A dynamic, ring-forming MucB / RseB-like protein influences spore shape in Bacillus subtilis
Source: PLoS Genet. 2020 Dec 14;16(12):e1009246. doi: 10.1371/journal.pgen.1009246 (PMC7769602; doi:10.1371/journal.pgen.1009246)
Supplement: S5 Table — *capital letters indicate restriction sites. (PDF) [file pgen.1009246.s019.pdf]

S5 TABLE: Oligonucleotide primers used in this study

Primers	Sequence*
**oAT005**	cgcAAGCTTacataaggaggaactactatgagtaaaggagaagaacttttc
**oAT006**	cgcCTCGAGgccgcttgagcctccagatgatcctttgtatagttcatccatgccatg
**oCR652**	cgcGAATTCgaatttctcgcgggcagattc
**oCR653**	gcgAAGCTTaattgatgtaaaaggaagaaatcgC
**oCR654**	cgcCTCGAGttgaaaaaggtgagaaaaagctttg
**oCR655**	gcgGGATCCggctaaagggaaacgatgtctaac
**oCR660**	cgcAAGCTTacataaggaggaactactatggtttcaaaaggcgaagaactg
**oCR663**	cggCTCGAGgccgcttgagcctccagatgatcccttataaagttcgtccatgcc
**oCR666**	gcgCTCGAGtgagctcccaccgctgctcccaccgcttgagcctccagatgatcc
**oCR735**	cgcAAGCTTcttgccgcttatgattgacgga
**oCR736**	cttgaaaatatcgaccttttccaagtgatcccctcccggacttcctatc
**oCR737**	ttggaaaaggtcgatattttcaagatgtcaaaaggcgaagagctgttt
**oCR738**	gcgCTCGAGgccggacttgtaaagttcattcatcccttctg
**oCR739**	gcgCTCGAGttggaaaaggtcgatattttcaagga
**oCR740**	cgcGGATCCttacaggatgatggcgattaagcc
**oHC007**	aaagcgattggccgaacaacgccagccaggtttatctgtttga
**oHC008**	tcaaacagataaacctggctggcgttgttcggccaatcgcttt
**oHC009**	ttaacaaaaaagatatgagcgcgtcatctgtcaaagtgatgga
**oHC010**	tccatcactttgacagatgacgcgctcatatcttttttgttaa
**oHC011**	tcaaaacgccgctggaactggcgcttggcgtcaagctgcttga
**oHC012**	tcaaaacgccgctggaactggcgcttggcgtcaagctgcttga
**oHC015**	ggaagcgcatcatcatgacggccggaggagaaaaatcatttac
**oHC016**	gtaaatgatttttctcctccggccgtcatgatgatgcgcttcc
**oHC017**	cgtacggaggagaaaaatcagctacgttaattcaggaaaaagc
**oHC018**	gctttttcctgaattaacgtagctgatttttctcctccgtacg
**oHC019**	aatcatttacgttaattcaggcaaaagcccagattgccaaggc
**oHC020**	gccttggcaatctgggcttttgcctgaattaacgtaaatgatt
**oHC037**	gcgCTCGAGgaatttctcgcgggcagattc
**oHC038**	cgcGGATCCttaatggtgatggtgatgatgtttcgatgattgtccctgca
**oJL001**	cgcGAATTCgcactccttgccctgaatcgaat
**oJL004**	cgcGGATCCttacttgtaaagttcattcatcccttctg
**oJL005**	gcgCTCGAGctcattttcttcttccggacgg
**oJL006**	gcgCTCGAGatgtcaaaaggcgaagagctg

Legend:

*****capital letters indicate restriction sites
