# Supplementary material for: A dynamic, ring-forming MucB / RseB-like protein influences spore shape in Bacillus subtilis
Source: PLoS Genet. 2020 Dec 14;16(12):e1009246. doi: 10.1371/journal.pgen.1009246 (PMC7769602; doi:10.1371/journal.pgen.1009246)
Supplement: S1 Methods — All construction details for plasmids used in this study. (PDF) [file pgen.1009246.s020.pdf]

S1 Methods

Plasmid construction

pBK2 [*ycgO::cfp (spec)*] was generated in a two-way ligation with *Hin*dIII-*Xho*I PCR product containing the *cfp* gene [oligonucleotide primers oCR660 & oCR663 and pDT19 (*amyE::PspoIIIA-RBSspoIIIAA-cfp-spoIIIAG (spec)* (Doan et al., 2009) as template] and pKM083 (*ycgO::spec*) cut with *Hin*dIII and *Xho*I. pKM083 is an ectopic integration vector for double crossover integration at the non-essential *ycgO* locus (Rudner, D.Z, unpublished).

pBK7 [*ycgO::SFgfp (spec)*] was generated in a two-way ligation with *Hin*dIII-*Xho*I PCR product containing the super-folder *gfp* gene (*SFgfp*) and pKM083. The PCR product was obtained through separate amplifications: 1) oligonucleotide primers oAT005 & oAT006 and pCR035 (*His-SUMO-sfGFP-spoIIQ^{ECD}* (Rodrigues et al., 2013)) as template, then 2) oAT005 & oCR666 to extend linker to 15 aa using the former PCR product as template. pKM083 (*ycgO::spec*) was cut with *Hin*dIII and *Xho*I. pKM083 is an ectopic integration vector for double crossover integration at the non-essential *ycgO* locus (Rudner, D.Z, unpublished)

pBK9 [*ycgO::PssdC-opt$_{RBS}$-SFgfp (spec)*] was generated in a two-way ligation with *Eco*RI-*Hin*dIII PCR product containing the *ssdC* promoter (oligonucleotide primers oCR652 & oCR653 and 168 genomic DNA as template) and pBK7 cut with *Eco*RI and *Hind*III.

pBK10 [*ycgO::PssdC-opt$_{RBS}$-SFgfp-ssdC (spec)*] was generated in a two-way ligation with *Xho*I-*Bam*HI PCR product containing the *ssdC* gene (oligonucleotide primers oCR654 & oCR655 and 168 genomic DNA as template) and pBK9 cut with *Xho*I and *Bam*HI.

pBK16 [*ycgO::PssdC-opt$_{RBS}$-cfp (spec)*] was generated in a two-way ligation with *Eco*RI-*Hin*dIII PCR product containing the *ssdC* promoter (oligonucleotide primers oCR652 & oCR653 and 168 genomic DNA as template) and pBK2 cut with *Eco*RI and *Hin*dIII.

pBK17 [*ycgO::PssdC-opt$_{RBS}$-cfp-ssdC (spec)*] was generated in a two-way ligation with XhoI-BamHI PCR product containing the *ssdC* gene (oligonucleotide primers oCR654 & oCR655 and 168 genomic DNA as template) and pBK16 cut with *Xho*I and *Bam*HI.

pHC7 [*ycgO::PssdC-opt$_{RBS}$-cfp-ssdC (P174A) (spec)*] was generated by site-directed mutagenesis of pBK17 using oligonucleotide primers oHC009 & oHC010.

pHC8 [*ycgO::PssdC-opt$_{RBS}$-cfp-ssdC (P238A) (spec)*] was generated by site-directed mutagenesis of pBK17 using oligonucleotide primers oHC011 & oHC012.

pHC9 [*ycgO::PssdC-opt$_{RBS}$-cfp-ssdC (Y261A) (spec)*] was generated by site-directed mutagenesis of pBK17 using oligonucleotide primers oHC015 & oHC016.

pHC10 [*ycgO::PssdC-opt$_{RBS}$-cfp-ssdC (F267A) (spec)*] was generated by site-directed mutagenesis of pBK17 using oligonucleotide primers oHC017 & oHC018.

pHC11 [*ycgO::PssdC-opt$_{RBS}$-cfp-ssdC (E272A) (spec)*] was generated by site-directed mutagenesis of pBK17 using oligonucleotide primers oHC019 & oHC020.

pHC22 [*ycgO::PssdC-ssdC-His6 (spec)*] was generated in a two-way ligation with *Xho*I-*Bam*HI PCR product containing the *ssdC* promoter and *ssdC* gene (oligonucleotide primers oHC037 & oHC038 and 168 genomic DNA as template) and pKM83 (*ycgO::spec*) cut with *Xho*I and *Bam*HI. pKM083 is an ectopic integration vector for double crossover integration at the non-essential *ycgO* locus

(Rudner, D.Z, unpublished). A sequence encoding His6 was incorporated into oHC038 to introduce a C-terminal His6 tag in-frame of SsdC.

pHC51 [*ycgO::PssdC-opt*$_{RBS}$*-cfp-ssdC (S118A) (spec)*] was generated by site-directed mutagenesis of pBK17 using oligonucleotide primers oHC007 & oHC008.

pJL1 [*ycgO::PsafA-safA-mYPET (spec)*] was generated in a three-way ligation with three pieces of DNA: 1) *Eco*RI-*Xho*I cut PCR product containing the *safA* gene (oligonucleotide primers oJL001 & oJL005 and 168 genomic DNA as template), 2) *Xho*I-*Bam*HI cut PCR product containing mYPET (oligonucleotide primers oJL004 & oJL006 and pAT29 plasmid DNA [*ycgO::PyqfZ-optRBS-mYPET-yqfZ (spec)*) as template], and pKM83 (*ycgO::spec*) cut with *Eco*RI and *Bam*HI. pKM83 (*ycgO::spec*) is a double crossover vector for ectopic integration at the non-essential locus *ycgO* (Rudner, D.Z, unpublished).

pJL6 [*ycgO::PspoIVA-mYPET-spoIVA (cat)*] was generated in a three-way ligation with three pieces of DNA, as follows: 1) *Hind*III-*Xho*I cut PCR product containing the promoter of *spoIVA* and *mYPET* (oligonucleotide primers oCR735 & oCR738 with the Gibson assembly product as template; the Gibson assembly product contained the promoter of *spoIVA* (oligonucleotide primers oCR735 & oCR736 and genomic DNA of 168 as template) and *mYPET* [oligonucleotide primers oCR737 & oCR738 and plasmid DNA of pAT29 (*ycgO::PyqfZ-optRBS-mYPET-yqfZ (spec)*) as template]; 2) *Xho*I-*Bam*HI cut PCR product containing the *spoIVA* open-reading frame (oligonucleotide primers oCR739 & oCR740 and 168 genomic DNA as template) and 3) plasmid pKM77 (*ycgO::cat*) cut with *Hind*III and *Bam*HI. pKM77 is a double crossover vector for ectopic integration at the non-essential locus *ycgO* (Rudner, D.Z, unpublished).

References

DOAN, T., MORLOT, C., MEISNER, J., SERRANO, M., HENRIQUES, A. O., MORAN, C. P., JR. & RUDNER, D. Z. 2009. Novel secretion apparatus maintains spore integrity and developmental gene expression in *Bacillus subtilis*. *PLoS Genet,* 5**,** e1000566.

RODRIGUES, C. D., MARQUIS, K. A., MEISNER, J. & RUDNER, D. Z. 2013. Peptidoglycan hydrolysis is required for assembly and activity of the transenvelope secretion complex during sporulation in *Bacillus subtilis*. *Mol Microbiol,* 89**,** 1039-52.

ZEIGLER, D. R., PRÁGAI, Z., RODRIGUEZ, S., CHEVREUX, B., MUFFLER, A., ALBERT, T., BAI, R., WYSS, M. & PERKINS, J. B. 2008. The origins of 168, W23, and other *Bacillus subtilis* legacy strains. *Journal of Bacteriology,* 190**,** 6983-6995.
